# Supplementary material for: A concise and scalable chemoenzymatic synthesis of prostaglandins
Source: Nat Commun. 2024 Mar 21;15:2523. doi: 10.1038/s41467-024-46960-y (PMC10957970; doi:10.1038/s41467-024-46960-y)
Supplement: Supplementary file 1 — Supplementary Information [file 41467_2024_46960_MOESM1_ESM.pdf]

# Supplementary Information

# A Concise and Scalable Chemoenzymatic Synthesis of Prostaglandins

Yunpeng Yin[1,3], Jinxin Wang[2,3], and Jian Li[1*]

[1]Frontiers Science Center for Transformative Molecules, School of Chemistry and Chemical Engineering, Shanghai Key Laboratory for Molecular Engineering of Chiral Drugs and Zhangjiang Institute for Advanced Study, Shanghai Jiao Tong University, Shanghai 200240, China

[2]Department of Phytochemistry, School of Pharmacy, Second Military Medical University, Shanghai 200433, China

[3] These authors contributed equally: Yunpeng Yin, Jinxin Wang.

[*]e-mail: jianlizcz@sjtu.edu.cn

**Contents**

**I Supplementary Notes 2**

**II Supplementary Methods 6**

**III Supplementary References 92**

# I Supplementary Notes

## General information

All reactions were carried out under an argon atmosphere with dry solvents under anhydrous conditions, unless otherwise noted. Pancreatin (from porcine pancreas, Aladdin), DMA (N,N-dimethylacetamide, extra dry, J&K), $NiBr_2$·glyme (98%, Bide), Raney Ni (≤150 μm, Aladdin) and zinc powder (600 mesh, Aladdin) was purchased and used directly. All other reagents were purchased at the highest commercial quality and used without further purification, unless otherwise stated. Solvents for chromatography were used as supplied by *GENERAL-REAGENT*®. Reactions were monitored by thin layer chromatography (TLC). TLC was performed with 0.25 mm Merck glass plates (silica gel 60 $F_{254}$) using shortwave UV light as the visualizing agent, $KMnO_4$, cerium ammonium molybdate (Hanessian's stain), phosphomolybdic acid and heat as developing agents. SiliaFlash® P60 silica gel (particle size: 40–63 μm, 230–400 mesh) was used for flash column chromatography. NMR spectra were recorded on a Bruker AVANCE NEO 400 MHz, Bruker AVANCE NEO 500 MHz and Bruker AVANCE NEO 700 MHz NMR spectrometer. The spectra were calibrated by using residual undeuterated solvents (for $^1$H NMR) and deuterated solvents (for $^{13}$C NMR) as internal references: undeuterated chloroform ($\delta_H$ = 7.26 ppm) and $CDCl_3$ ($\delta_C$ = 77.16 ppm). Melting points (m.p.) were recorded on an SGW X-4B apparatus. Specific rotations were recorded on Anton Paar MCP 5500. High-resolution mass spectra (HRMS) were recorded on a Waters Premier GC-TOF mass spectrometer (compound **7**) and Waters G2-XS/APGC(all other compounds). Expression vectors were obtained via DNA synthesis from Universe Gene Technology (Tianjin) Co., Ltd. and were used directly to competent *E. coli* BL21(DE3). Competent *E. coli* BL21(DE3) strains were purchased from Sangon Biotech (Shanghai) Co., Ltd. All *E. coli* strains generated in this work are stored as glycerol stocks at −80 ºC.

**Protein and DNA Sequences**

Protein sequence of $CHMO_{rhodo1}$:

MTAQISPTVVDAVVIGAGFGGIYAVHKLHNEQGLTVVGFDKADGPGGTWYWNRYPGALSDTESHLYRFSFDRDLLQ
DGTWKTTYITQPEILEYLESVVDRFDLRRHFRFGTEVTSAIYLEDENLWEVSTDKGEVYRAKYVVNAVGLLSAINF
PDLPGLDTFEGETIHTAAWPEGKNLAGKRVGVIGTGSTGQQVITALAPEVEHLTVFVRTPQYSVPVGNRPVTKEQI
DAIKADYDGIWDSVKKSAVAFGFEESTLPAMSVSEEERNRIFQEAWDHGGGFRFMFGTFGDIATDEAANEAAASFI
RSKIAEIIEDPETARKLMPTGLYAKRPLCDNGYYEVYNRPNVEAVAIKENPIREVTAKGVVTEDGVLHELDVLVFA
TGFDAVDGNYRRIEIRGRNGLHINDHWDGQPTSYLGVTTANFPNWFMVLGPNGPFTNLPPSIETQVEWISDTVAYA
ERNEIRAIEPTPEAEEEWTQTCTDIANATLFTRGDSWIFGANVPGKKPSVLFYLGGLGNYRNVLAGVVADSYRGFE
LKSAVPVTA

DNA sequence of $CHMO_{rhodo1}$:

ATGACCGCACAGATTAGCCCGACCGTTGTGGATGCAGTTGTGATTGGTGCAGGTTTTGGCGGTATTTATGCCGTTC
ATAAACTGCATAATGAACAGGGTCTGACCGTGGTGGGCTTTGATAAAGCCGATGGTCCGGGCGGTACCTGGTATTG
GAATCGTTATCCGGGTGCCCTGAGTGATACCGAAAGCCATCTGTATCGTTTTAGTTTTGATCGCGATCTGCTGCAG
GATGGTACCTGGAAAACCACCTATATTACCCAGCCGGAAATTCTGGAATATCTGGAAAGCGTGGTGGATCGTTTTG
ATCTGCGCCGTCATTTTCGCTTTGGTACCGAAGTTACCAGTGCCATTTATCTGGAAGATGAAAATCTGTGGGAAGT
GAGTACCGATAAAGGCGAAGTGTATCGCGCAAAATATGTTGTGAATGCAGTTGGTCTGCTGAGCGCCATTAATTTT
CCGGATCTGCCGGGTCTGGATACCTTTGAAGGCGAAACCATTCATACCGCAGCATGGCCGGAAGGCAAAAATCTGG
CCGGCAAACGTGTTGGCGTGATTGGTACCGGCAGCACCGGCCAGCAGGTTATTACCGCACTGGCACCGGAAGTTGA
ACATCTGACCGTTTTTGTGCGCACCCCGCAGTATAGCGTTCCGGTGGGTAATCGCCCGGTTACCAAAGAACAGATT
GATGCAATTAAAGCCGATTATGATGGTATTTGGGATAGCGTTAAAAAAAGTGCCGTTGCATTTGGTTTTGAAGAAA
GTACCCTGCCGGCCATGAGTGTGAGCGAAGAAGAACGCAATCGCATTTTTCAGGAAGCCTGGGATCATGGCGGTGG
TTTTCGCTTTATGTTTGGTACCTTTGGCGATATTGCCACCGATGAAGCAGCAAATGAAGCCGCAGCAAGTTTTATT
CGCAGCAAAATTGCAGAAATTATCGAAGATCCGGAAACCGCCCGCAAACTGATGCCGACCGGTCTGTATGCCAAAC
GCCCGCTGTGTGATAATGGTTATTATGAAGTTTACAACCGTCCGAATGTTGAAGCAGTGGCAATTAAAGAAAATCC
GATTCGTGAAGTTACCGCCAAAGGCGTGGTGACCGAAGATGGTGTTCTGCATGAACTGGATGTGCTGGTTTTTGCC
ACCGGCTTTGATGCCGTGGATGGTAATTATCGTCGCATTGAAATTCGCGGCCGCAATGGCCTGCATATTAATGATC
ATTGGGATGGTCAGCCGACCAGCTATCTGGGCGTTACCACCGCAAATTTTCCGAATTGGTTTATGGTTCTGGGTCC
GAATGGCCCGTTTACCAATCTGCCGCCGAGCATTGAAACCCAGGTGGAATGGATTAGCGATACCGTGGCCTATGCA
GAACGCAATGAAATTCGTGCAATTGAACCGACCCCGGAAGCCGAAGAAGAATGGACCCAGACCTGTACCGATATTG
CCAATGCCACCCTGTTTACCCGTGGTGATAGTTGGATTTTTGGCGCAAATGTTCCGGGTAAAAAACCGAGCGTTCT
GTTTTATCTGGGCGGCCTGGGCAATTATCGTAATGTTCTGGCAGGCGTGGTTGCCGATAGCTATCGCGGCTTTGAA
CTGAAAAGCGCCGTTCCGGTTACCGCA

Protein sequence of GDH$_{P.\ megaterium}$:

MYTDLKDKVVVITGGSTGLGRAMAVRFGQEEAKVVINYYNNEEEALDAKKEVEEAGGQAIIVQGDVTKEEDVVNLV
QTAIKEFGTLDVMINNAGVENPVPSHELSLDNWNKVIDTNLTGAFLGSREAIKYFVENDIKGNVINMSSVHEMIPW
PLFVHYAASKGGMKLMTETLALEYAPKGIRVNNIGPGAMNTPINAEKFADPVQRADVESMIPMGYIGKPEEVAAVA
AFLASSQASYVTGITLFADGGMTKYPSFQAGRG

DNA sequence of GDH$_{P.\ megaterium}$:

ATGTACACCGATCTGAAAGATAAAGTGGTGGTTATTACCGGTGGTAGCACCGGCCTGGGTCGTGCAATGGCCGTTC
GCTTTGGCCAGGAAGAAGCAAAAGTTGTTATTAATTACTACAACAACGAGGAAGAAGCCCTGGATGCCAAAAAAGA
AGTGGAAGAAGCAGGTGGCCAGGCAATTATTGTTCAGGGCGATGTTACCAAAGAAGAAGATGTGGTGAATCTGGTG
CAGACCGCCATTAAAGAATTTGGTACCCTGGATGTTATGATTAATAATGCCGGTGTGGAAAATCCGGTTCCGAGCC
ATGAACTGAGCCTGGATAATTGGAATAAAGTGATTGATACCAACCTGACCGGTGCATTTCTGGGTAGTCGCGAAGC
AATTAAATATTTTGTTGAAAACGACATCAAGGGTAATGTGATTAATATGAGCAGTGTGCATGAAATGATTCCGTGG
CCGCTGTTTGTTCATTATGCCGCCAGCAAAGGCGGCATGAAACTGATGACCGAAACCCTGGCCCTGGAATATGCAC
CGAAAGGCATTCGTGTTAATAATATTGGCCCGGGCGCAATGAATACCCCGATTAATGCCGAAAAATTTGCAGATCC
GGTGCAGCGCGCAGATGTTGAAAGCATGATTCCGATGGGCTATATTGGTAAACCGGAAGAAGTTGCAGCCGTTGCC
GCCTTTCTGGCCAGTAGCCAGGCAAGTTATGTTACCGGTATTACCCTGTTTGCAGATGGTGGTATGACCAAATATC
CGAGTTTTCAGGCCGGCCGTGGT

Protein sequence of Opt-13:

MLPKLVITHRVHEEILQLLAPHCELITNQTDSTLTREEILRRCRDAQAMMAFMPDRVDADFLQACPELRVIGCALK
GFDNFDVDACTARGVWLTFVPDLLTVPTAELAIGLAVGLGRHLRAADAFVRSGKFKGWQPHFYGTGLDNSTVGFLG
MGAIGLAMADRLQGWGATLQYHAAKALDTQTEQRLGLRQVACSELFASSDFILLALPLNADTLHLVNAELLALVRP
GALLVNPCRGSVVDEAAVLAALERGQLGGYAADVFEMEDWARADRPLCIDPALLAHPNTLFTPHIGSAVRAVRLEI
ERCAAQNILQALAGERPINAVNRLPKANPAAD

DNA sequence of Opt-13:

GTCTGCGGCAGGATTGGCCTTGGGCAGACGGTTCACAGCGTTGATTGGGCGCTCACCTGCCAATGCCTGGAGGATG
TTCTGCGCTGCACAACGTTCAATCTCCAGGCGCACCGCGCGCACTGCCGACCCTATGTGCGGAGTGAACAGCGTAT
TCGGATGCGCGAGCAGCGCAGGATCGATGCACAGCGGCCGGTCCGCGCGAGCCCAGTCTTCCATTTCGAATACATC
CGCCGCATACCCGCCGAGCTGGCCTCGCTCAAGCGCCGCGAGCACGGCGGCTTCATCCACTACCGAGCCACGACAG
GGGTTTACAAGCAGAGCGCCCGGCCGTACGAGGGCAAGCAGCTCGGCGTTGACCAGATGCAGGGTATCGGCATTCA
AGGGAAGCGCCAGCAGGATGAAGTCCGAGCTGGCGAAGAGTTCGCTGCACGCCACCTGGCGCAGGCCGAGCCGTTG

CTCGGTTTGTGTATCCAGAGCCTTCGCCGCGTGGTACTGCAGGGTCGCGCCCCATCCCTGCAAGCGATCAGCCATG
GCCAGTCCGATGGCGCCCATGCCAAGGAAGCCGACCGTAGAGTTATCCAGCCCCGTGCCGTAGAAATGTGGTTGCC
AGCCCTTGAACTTGCCAGAGCGGACGAACGCATCTGCTGCCCGCAGATGCCGCCCCAGCCCCACCGCCAGTCCGAT
CGCCAGCTCGGCAGTCGGGACCGTCAACAGATCAGGCACGAAGGTCAGCCAGACCCCGCGGGCAGTACAGGCGTCC
ACATCGAAATTGTCGAAGCCCTTGAGCGCGCAGCCGATTACACGCAGCTCAGGGCAGGCTTGAAGAAAGTCTGCAT
CGACCCGATCGGGCATGAACGCCATCATCGCCTGAGCATCGCGACAGCGGCGCAGAATTTCCTCGCGCGTCAGCGT
GCTGTCGGTCTGGTTGGTTATCAGCTCGCAATGTGGCGCCAGCAGTTGCAGGATCTCTTCGTGTACTCGGTGAGTT
ATAACGAGTTTCGGCAGCAT

**Generation of Expression Vectors**

The respective gene fragment of CHMO, GDH and Opt-13 was obtained via DNA synthesis from Universe Gene Technology (Tianjin) Co., Ltd. $CHMO_{rhodo1}$ gene was inserted between NdeI and XhoI restriction sites of pET-28a to yield vector pET-28a-$CHMO_{rhodo1}$. GDH and Opt-13 genes were inserted between NcoI and XhoI restriction sites of pRSF-1b to yield vector pRSF-GDH and pRSF-Opt13. The vector was used directly to transform competent *E. coli* strain BL21(DE3).

# II Supplementary Methods

## Overall Synthetic Routes:

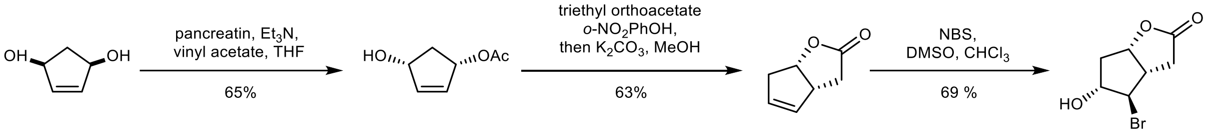


**(±)-12** **11** **9** **8**

**Supplementary Figure 1.** Synthesis route 1 for common intermediate **8**.

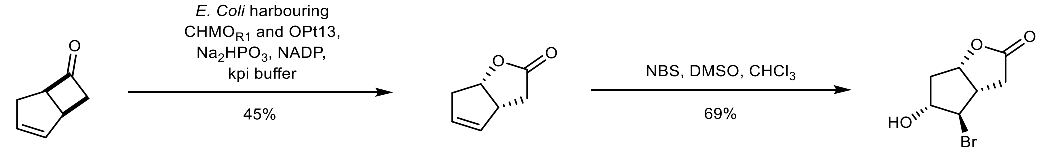


(±)–**10** **9** **8**

**Supplementary Figure 2.** Synthesis route 2 for common intermediate **8**.

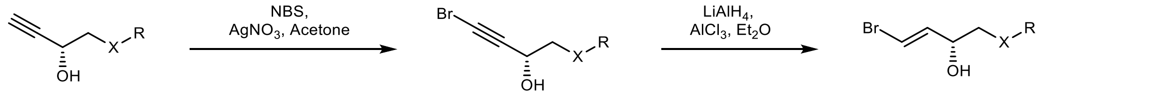


**26**: X = $CH_2$, R = *n*-Pr
**26a**: X = $CH_2$, R = Ph
**26b**: X = O, R = 3-Chlorophenyl
**26c**: X = O, R = 3-(Trifluoromethyl)phenyl

**25**: X = $CH_2$, R = *n*-Pr
**25a**: X = $CH_2$, R = Ph
**25b**: X = O, R = 3-Chlorophenyl
**25c**: X = O, R = 3-(Trifluoromethyl)phenyl

**16**: X = $CH_2$, R = *n*-Pr
**16a**: X = $CH_2$, R = Ph
**16b**: X = O, R = 3-Chlorophenyl
**16c**: X = O, R = 3-(Trifluoromethyl)phenyl

**Supplementary Figure 3.** Synthesis of side chains **16, 16a, 16b and 16c**.

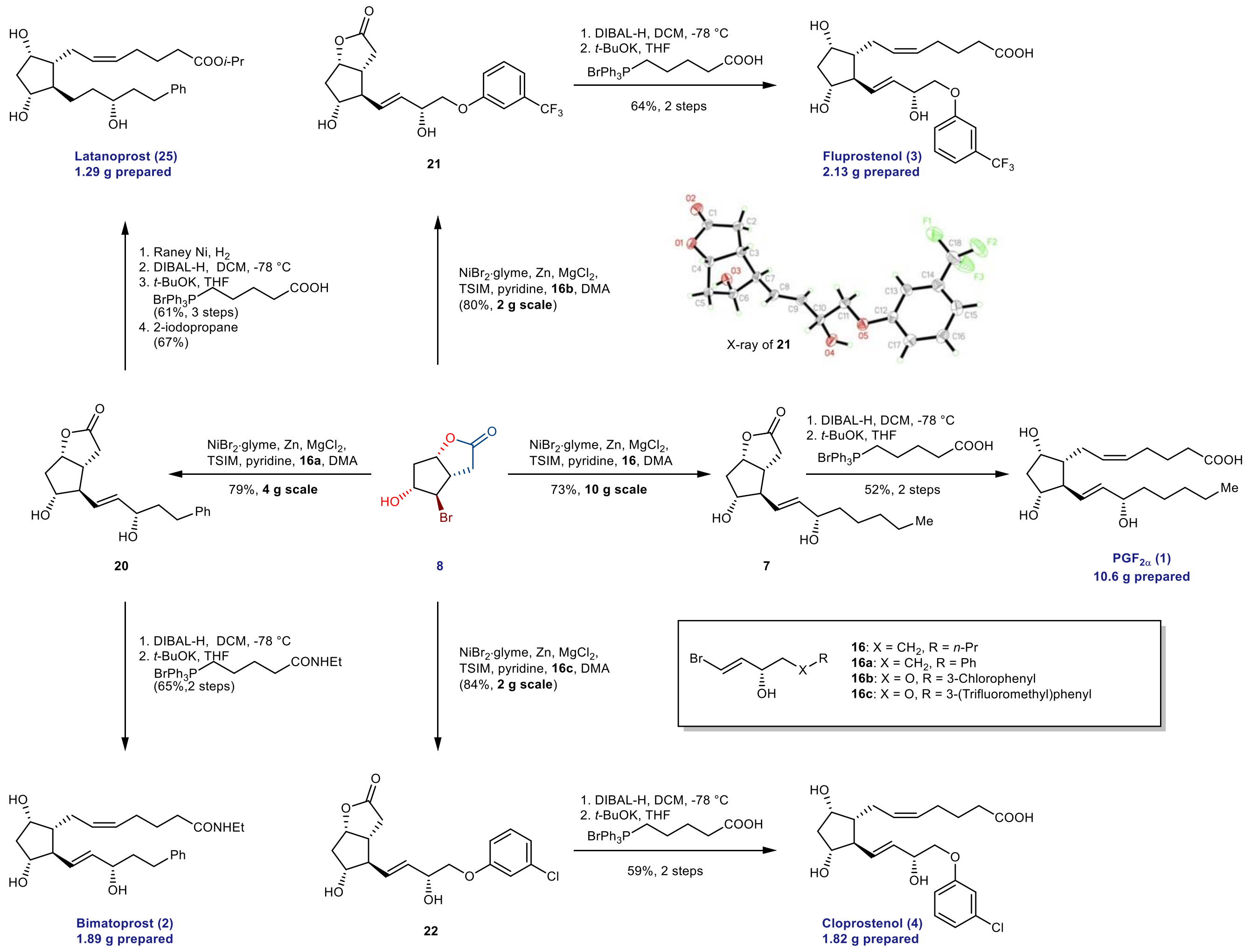


**Supplementary Figure 4.** Synthesis of **PGF$_{2\alpha}$ (1)**, **Bimatoprost (2)**, **Fluprostenol (3)**, **Cloprostenol (4) and Latanoprost (25)** from common intermediate **8**.

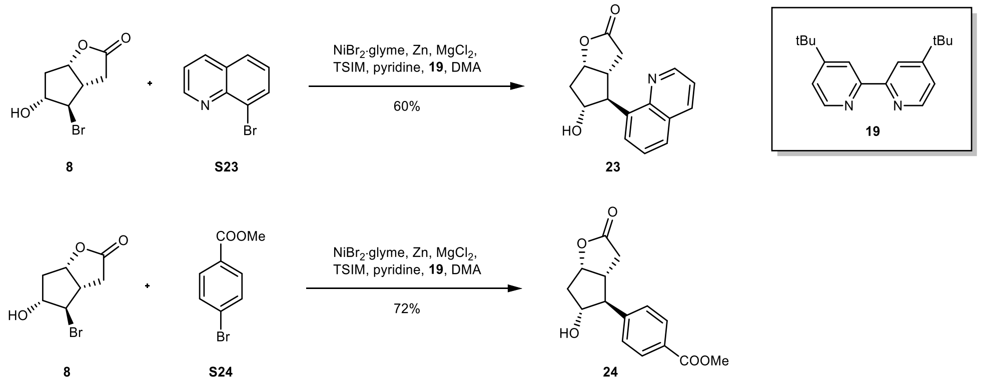


**Supplementary Figure 5.** Synthesis of compound **23** and **24** from common intermediate **8**.

**Experimental Procedures and Spectroscopic Data of Compounds:**

Synthesis of compound **11**:

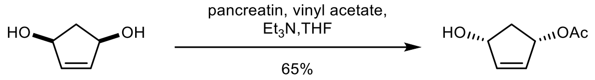


**(±)-12** **11**

Synthesis of compound **11**:

To a stirred solution of **(±)-12** (30.0 g, 300 mmol, 1.0 equiv.) in THF (600 mL) was sequentially added pancreatin (113 g), vinyl acetate (194 mL, 2.10 mol, 7.0 equiv.), $Et_3N$ (29.2 mL, 210 mmol, 0.7 equiv.). The reaction mixture was shaken (150 rpm) at 23 °C until the starting material was completely consumed. The mixture was centrifuged (4 °C, 15 min, 4200 rpm), filtered, washed with EtOAc (3 × 200 mL) and concentrated in *vacuo*. The crude material was purified by flash column chromatography (3:1 petroleum ether:EtOAc) to yield compound **11** (27.7 g, 65% yield, 94.5% ee) as a white solid.

**$^1$H NMR (500 MHz, $CDCl_3$):** δ 6.15 – 6.09 (m, 1H), 6.02 – 5.96 (m, 1H), 5.53 – 5.47 (m, 1H), 4.75 – 4.70 (m, 1H), 2.80 (ddd, *J* = 14.7, 7.4, 7.4 Hz, 1H), 2.05 (s, 3H), 1.66 (ddd, *J* = 14.7, 3.8, 3.8 Hz, 1H).

**$^{13}$C NMR (126 MHz, $CDCl_3$):** δ 171.0, 138.6, 132.6, 77.2, 74.9, 40.6, 21.3.

**HRMS (*m/z*)**: $[M+Na]^+$ calcd for $C_7H_{10}O_3Na^+$ 165.0522, found 165.0527.

**$[\alpha]_D^{20}$** = −59.5 (*c* = 1.0 in $CHCl_3$, 94.5% ee).

Synthesis of compound **9**:

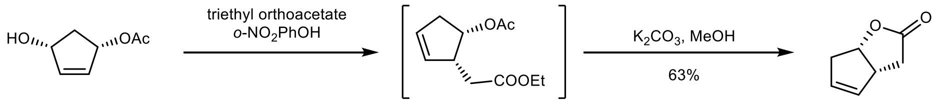


**11** **13** **9**

To a stirred solution of compound **11** (24.0 g, 169 mmol, 1.0 equiv.) in triethyl orthoacetate (155 mL) was added *o*-$NO_2$PhOH (2.35 g, 16.9 mmol, 0.1 equiv.) at room temperature. The reaction mixture was allowed to stir at 180 °C for 24 hours until the starting material was completely consumed. Sequentially

the mixture was added MeOH (169 mL) and $K_2CO_3$ (46.7 g, 338 mmol, 2.0 equiv.) at 0 °C and stirred at room temperature for 1 hour. The reaction was quenched with water (250 mL) and extracted with DCM (3 × 300 mL). The combined organic extracts were washed with brine, dried over anhydrous $Na_2SO_4$, filtered, and concentrated in *vacuo*. The crude material was purified by flash column chromatography (5:1 petroleum ether:EtOAc) to yield compound **9** (13.2 g, 63% yield, 94.5% ee) as a white solid.

**$^1$H NMR (500 MHz, CDCl$_3$):** δ 5.82 – 5.76 (m, 1H), 5.61 – 5.55 (m, 1H), 5.16 – 5.10 (m, 1H), 3.55 – 3.47 (m, 1H), 2.77 (dd, *J* = 18.0, 9.7 Hz, 1H), 2.73 – 2.68 (m, 2H), 2.44 (dd, *J* = 18.0, 1.6 Hz, 1H).

**$^{13}$C NMR (126 MHz, CDCl$_3$):** δ 176.9, 131.4, 129.9, 83.2, 45.7, 39.7, 33.4.

**HRMS (*m/z*)**: $[M]^+$ calcd for $C_7H_8O_2^+$ 124.0519, found 124.0521.

$[\alpha]_D^{20}$ = −91.2 (*c* = 1.0 in $CHCl_3$, 95.0% ee).

Synthesis of compound **8:**

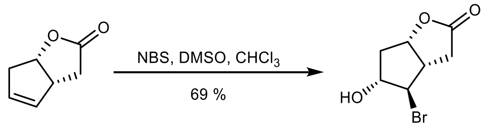


9 8

To a stirred solution of compound **7** (12.4 g, 100 mmol, 1.0 equiv.) in $CHCl_3$/DMSO (1000 mL, 9:1) was added NBS (26.7 g, 150 mmol, 1.5 equiv.) and $H_2O$ (3.60 g, 200 mmol, 2.0 equiv.) at 10 °C. After stirring at 10 °C for 30 minutes, the reaction mixture was stirred at room temperature for 2 hours. Then another portion NBS (8.90 g, 50 mmol, 0.5 equiv.) was added and the mixture was continued to stir at room temperature for 1 hour. The reaction was quenched with saturated aq. $NH_4Cl$ (200 mL) and saturated aq. $Na_2S_2O_3$ (200 mL), extracted with DCM (3 × 500 mL). The combined organic extracts were washed with brine (500 mL), dried over anhydrous $Na_2SO_4$, filtered, and concentrated in *vacuo*. The crude material was purified by flash column chromatography (1:1 petroleum ether:EtOAc) to yield compound **8** (15.3 g, 69% yield, 95.0% ee) as a white solid. This sample was further recrystallized from EtOAc. For recrystallized **8**, ee = 99.7%. Crystallographic data for the structures reported in this Article have been

deposited at the Cambridge Crystallographic Data Centre, under deposition numbers CCDC 2312333 (**8**). Copies of the data can be obtained free of charge via https://www.ccdc.cam.ac.uk/structures/.

**$^1$H NMR (500 MHz, $CDCl_3$):** δ 5.19 – 5.13 (m, 1H), 4.49 (ddd, *J* = 5.3, 3.1, 3.1 Hz, 1H), 4.11 – 4.03 (m, 1H), 3.40 – 3.29 (m, 1H), 2.87 (dd, *J* = 18.6, 11.5 Hz, 1H), 2.71 – 2.56 (m, 2H), 2.24 – 2.15 (m, 1H).

**$^{13}$C NMR (126 MHz, $CDCl_3$):** δ 176.8, 84.0, 79.6, 58.2, 48.2, 38.6, 35.7.

**HRMS (*m/z*):** $[M+H]^+$ calcd for $C_7H_{10}O_3Br^+$ 220.9808, found 220.9809.

**$[\alpha]_D^{20}$** = −15.3 (*c* = 1.0 in $CHCl_3$, 99.7% ee).

**[m.p.]** = 124–126 °C

Synthesis of compound **9**:

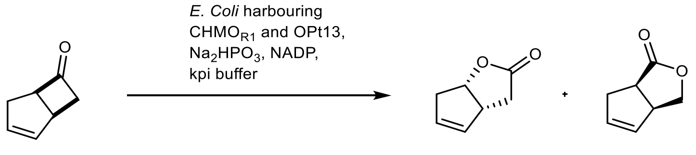


(±)-10 9 14

An overnight culture of *E. coli* BL21(DE3) cells harboring pET-22b(+)-based vector for expressing the appropriate CHMO$_{rhodo1}$ and pRSF-Opt13 plasmid was used to inoculate 500 mL TB media (in 2 L Erlenmeyer flask) containing 50 µg/mL kanamycin, 50 µg/mL ampicillin. The cultures were shaken at 250 rpm at 37 ˚C until an optical density of OD600 = 0.7 – 1.0 was reached. The cultures were cooled on ice for 20 minutes and then induced with riboflavin and IPTG to final concentrations of 1.0 µM and 0.5 mM, respectively. The cultures were shaken at 150 rpm at 20 ˚C for a further 20 hours. Cells were harvested by centrifugation (4 ˚C, 15 min, 4,121 g), resuspended in 500 mL kPi buffer (50 mM, pH = 8.00) to an OD600 = 10 into a 2 L Erlenmeyer flask. To the mixture was sequentially added a pre-dissolved solution of 4.5 g ketone **10** in 25 mL DMSO, $Na_2NADP\cdot4H_2O$ (654 mg, 0.83 mmol), $Na_2HPO_3\cdot5H_2O$ (9.3 g, 43.0 mmol). The Erlenmeyer flask was shaken at 150 rpm at 25 ˚C for 20 hours. The mixture was extracted with EtOAc (300 mL × 3), and the combined organic extracts were

concentrated in vacuo gives 1:1 mixture of lactone **9** and **14**. The crude material was purified by flash column chromatography (5:1 petroleum ether:EtOAc) to yield compound **9** (2.32 g, 45% yield, 95.0% ee) as a white solid.

The large-scale fermentation operations were carried out in parallel with 24 × 2L Erlenmeyer flasks. According above procedure, 108 g ketone **10** could be taken in one batch. The crude product (>100g) was purified by flash column chromatography [Dimensions of column: 120 mm × 420 mm (D×L); Silica gel: SiliaFlash® P60, 40–63 µm, 230–400 mesh; Flow rate: ca. 100 mL min$^{-1}$; Eluent: 10:1 (ca. 10 L) to 8:1 (ca. 10 L) to 6:1 (ca. 20 L) to 5:1 (ca. 30 L) petroleum ether:EtOAc] to yield pure compound **9** (**TLCs 5, 6, and 7**). The overlapped fractions (**TLCs 3 to 4**) are collected and re-separated using a chromatographic column that is proportionally reduced in size. After purified the compound **9** was yield as a white solid (55.8 g, 45% yield, 95.0% ee). The spectra of this sample are identical to the sample prepared through the route 1 (the compound **9** on page S8).

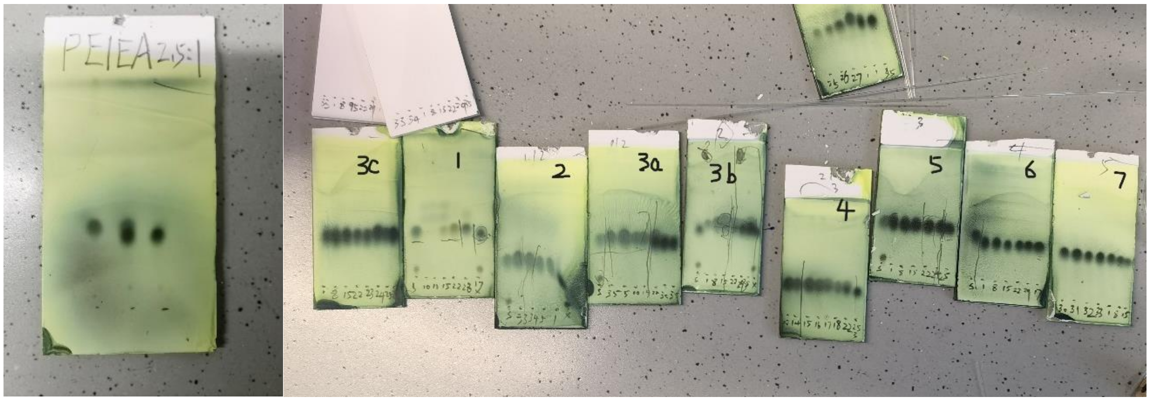


**Supplementary Figure 6. The TLCs of flash column chromatography**

Synthesis of compound **8**:

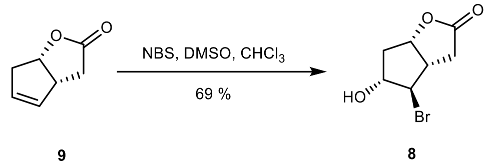

The compound **8** (15.2 g, 69% yield, 95.0% ee) was obtained as a white solid from compound **9** (12.4 g, 100 mmol) with the same procedure as route 1 mentioned. The spectra of this sample are identical to the sample prepared through the route 1 (the compound **8** on page S9).

Synthesis of side chains **16, 16a, 16b, 16c**:

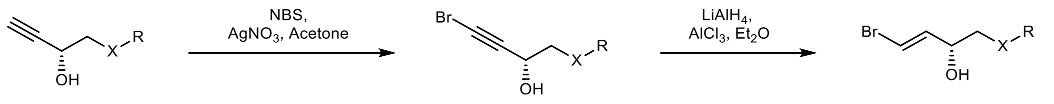


**26**: X = $CH_2$, R = *n*-Pr
**26a**: X = $CH_2$, R = Ph
**26b**: X = O, R = 3-Chlorophenyl
**26c**: X = O, R = 3-(Trifluoromethyl)phenyl

**25**: X = $CH_2$, R = *n*-Pr
**25a**: X = $CH_2$, R = Ph
**25b**: X = O, R = 3-Chlorophenyl
**25c**: X = O, R = 3-(Trifluoromethyl)phenyl

**16**: X = $CH_2$, R = *n*-Pr
**16a**: X = $CH_2$, R = Ph
**16b**: X = O, R = 3-Chlorophenyl
**16c**: X = O, R = 3-(Trifluoromethyl)phenyl

General procedure following the literature reports[1]:

To a stirred solution of compound **26** (1.0 equiv.) in acetone (0.2 M) was added NBS (1.2 equiv.) and $AgNO_3$ (0.1 equiv.). The reaction mixture was stirred at room temperature for 1.5 hours then poured to $H_2O$ and extracted with EtOAc. The combined organic extracts were washed with brine, dried over anhydrous $Na_2SO_4$, filtered, and concentrated in *vacuo*. The crude material was purified by flash column chromatography (10:1 petroleum ether:EtOAc) to yield compound **25**.

To a stirred solution of $LiAlH_4$ (2.0 equiv.) in dry $Et_2O$ (0.5 M) was added $AlCl_3$ (1.5 equiv.) carefully at 0 °C. A solution of compound **25** (1.0 equiv.) in dry $Et_2O$ (1.5 M) was added dropwise by syringe and the reaction mixture stirred at room temperature for 1 hour. After cooling to 0 °C, $H_2O$ (0.75 mL/10 mmol compound **25**) was carefully added dropwise followed by 0.5 N aq. NaOH (0.75 mL/10 mmol compound **25**) solution and then a further portion of $H_2O$ (2.25 mL/10 mmol compound **25**). The mixture was stirred at room temperature for 30 minutes, $MgSO_4$ was added and the mixture was stirred for a further 15 minutes to prior to sinter filtration, wash with $Et_2O$. After concentrating in *vacuo*, the crude material was purified by flash column chromatography (10:1 petroleum ether:EtOAc) to yield compound **16**.

Synthesis of compound **16**:

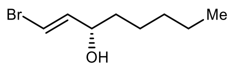

**16**

The compound **16** (48.3 g, 233 mmol, 81% yield over 2 steps) was obtained as a colorless oil from compound **26** (36.5 g, 289 mmol) via General Procedure.

**$^{1}$H NMR (500 MHz, CDCl$_3$):** δ 6.33 (dd, *J* = 13.6, 1.0 Hz, 1H), 6.23 (dd, *J* = 13.6, 6.6 Hz, 1H), 4.16 – 4.08 (m, 1H), 1.61 – 1.47 (m, 3H), 1.44 – 1.23 (m, 6H), 0.89 (t, *J* = 6.9 Hz, 3H).

**$^{13}$C NMR (126 MHz, CDCl$_3$):** δ 140.6, 107.1, 72.8, 37.0, 31.8, 25.0, 22.7, 14.1.

**$[\alpha]_D^{20}$** = −4.7 (*c* = 1.0 in CHCl$_3$).

Synthesis of compound **16a**:

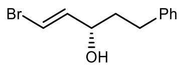

**16a**

The compound **16a** (11.6 g, 48.1 mmol, 80% yield over 2 steps) was obtained as a colorless oil from compound **26a** (9.62 g, 60.0 mmol) via General Procedure.

**$^{1}$H NMR (500 MHz, CDCl$_3$):** δ 7.32 – 7.27 (m, 2H), 7.23 – 7.17 (m, 3H), 6.35 (dd, *J* = 13.6, 0.8 Hz, 1H), 6.27 (dd, *J* = 13.6, 6.5 Hz, 1H), 4.17 – 4.11 (m, 1H), 2.82 – 2.66 (m, 2H), 1.96 – 1.81 (m, 2H).

**$^{13}$C NMR (126 MHz, CDCl$_3$):** δ 141.4, 140.3, 128.6, 128.6, 126.2, 107.6, 72.0, 38.3, 31.5.

**HRMS (*m/z*):** $[M–H]^-$ calcd for $C_{11}H_{12}OBr^-$ 239.0077, found 239.0077.

**$[\alpha]_D^{20}$** = +20.7 (*c* = 1.0 in CHCl$_3$).

Synthesis of compound **16b**:

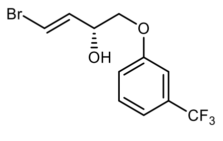

**16b**

The compound **16b** (7.96 g, 25.6 mmol, 84% yield over 2 steps) was obtained as a colorless oil from compound **26b** (7.00 g, 30.4 mmol) via General Procedure.

**$^{1}$H NMR (500 MHz, CDCl$_3$):** δ 7.41 (dd, *J* = 8.0, 8.0 Hz, 1H), 7.28 – 7.23 (m, 1H), 7.14 (s, 1H), 7.11 – 7.06 (m, 1H), 6.58 (dd, *J* = 13.6, 1.3 Hz, 1H), 6.34 (dd, *J* = 13.6, 6.0 Hz, 1H), 4.63 – 4.52 (m, 1H), 4.06 (dd, *J* = 9.3, 3.5 Hz, 1H), 3.95 (dd, *J* = 9.2, 7.3 Hz, 1H).

**$^{13}$C NMR (126 MHz, CDCl$_3$):** δ 158.4, 135.2, 132.1 (q, *J* = 32.5 Hz), 130.3, 123.9 (q, *J* = 272.4 Hz), 118.3 (q, *J* = 3.8 Hz), 118.2 – 118.1 (m), 111.6 (q, *J* = 3.9 Hz), 110.0, 71.3, 70.9.

**$^{19}$F NMR (471 MHz, CDCl$_3$):** δ -62.7.

**HRMS (*m/z*):** [M–H]$^-$ calcd for $C_{11}H_9O_2F_3Br^-$ 308.9744, found 308.9734.

**$[\alpha]_D^{20}$** = −6.3 (*c* = 1.0 in $CHCl_3$).

Synthesis of compound **16c**:

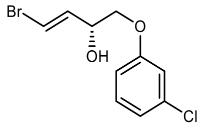


**16c**

The compound **16c** (9.09 g, 32.8 mmol, 81% yield over 2 steps) was obtained as a colorless oil from compound **26c** (8.01 g, 40.7 mmol) via General Procedure.

**$^{1}$H NMR (500 MHz, CDCl$_3$):** δ 7.21 (dd, *J* = 8.1, 8.1 Hz, 1H), 6.97 (d, *J* = 8.0, Hz, 1H), 6.91 (s, 1H), 6.80 (d, *J* = 8.3 Hz, 1H), 6.56 (d, *J* = 13.6 Hz, 1H), 6.32 (dd, *J* = 13.6, 5.9 Hz, 1H), 4.59 – 4.50 (m, 1H), 4.01 (dd, *J* = 9.4, 3.4 Hz, 1H), 3.93 – 3.85 (m, 1H).

**$^{13}$C NMR (126 MHz, CDCl$_3$):** δ 159.0, 135.2, 135.1, 130.5, 121.8, 115.2, 113.1, 109.9, 71.2, 70.8.

**HRMS (*m/z*):** [M–H]$^-$ calcd for $C_{10}H_9O_2ClBr^-$ 274.9480, found 274.9472.

**$[\alpha]_D^{20}$** = −5.6 (*c* = 1.0 in $CHCl_3$).

Synthesis of compound **7**:

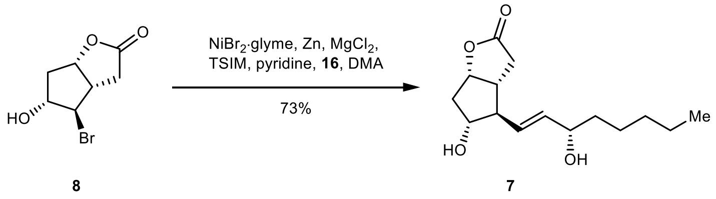


To a stirred solution of compound **8** (13.6 g, 61.5 mmol, 1.0 equiv.) in DMA (120 mL) was added TSIM (9.9 mL, 67.7 mmol, 1.1 equiv.). After stirring at room temperature for 1 hour, the mixture was added to another flask charged with $NiBr_2$·glyme (2.85 g, 9.23 mmol, 0.15 equiv.), $MgCl_2$ (5.86 g, 61.5 mmol, 1.0 equiv.), zinc powder (8.04 g, 123 mmol, 2.0 equiv.) and compound **16** (25.5 g, 123 mmol, 2.0 equiv.) via syringe. Followed by adding DMA (285 mL) and pyridine (5.0 mL, 61.5 mmol, 1.0 equiv.), the mixture was stirred at room temperature for 20 hours. After cooling to 0 °C, the mixture was added 1 N aq. HCl (360 mL) and stirred at room temperature for 15 minutes. Then the mixture was extracted with EtOAc (5 × 600 mL), and the combined organic extracts were dried over anhydrous $Na_2SO_4$, filtered, and concentrated in *vacuo* (*vacuubrand*® RZ 6). After removing most of DMA, the concentrated mixture was diluted with EtOAc (1000 mL) and washed with 10% aq. NaCl (3 × 200 mL), the organic phase was dried over anhydrous $Na_2SO_4$, filtered, and concentrated in *vacuo*. The crude material was purified by flash column chromatography (1:1 petroleum ether:acetone) to yield compound **7** (12.1 g, 73% yield) as a pale yellow oil.

**$^1$H NMR (500 MHz, $CDCl_3$):** δ 5.58 (dd, *J* = 15.3, 7.2 Hz, 1H), 5.43 (dd, *J* = 15.3, 8.5 Hz, 1H), 4.88 (ddd, *J* = 7.0, 7.0, 2.9 Hz, 1H), 4.03 (ddd, *J* = 6.7, 6.7, 6.7 Hz, 1H), 3.92 (ddd, *J* = 7.6, 7.6, 7.6 Hz, 1H), 2.71 (dd, *J* = 18.1, 9.5 Hz, 1H), 2.60 – 2.47 (m, 2H), 2.40 (d, *J* = 18.1 Hz, 1H), 2.24 (ddd, *J* = 8.5, 8.5, 8.5 Hz, 1H), 1.91 (ddd, *J* = 14.5, 8.0, 2.7 Hz, 1H), 1.60 – 1.50 (m, 1H), 1.50 – 1.41 (m, 1H), 1.39 – 1.20 (m, 6H), 0.88 (t, *J* = 6.4 Hz, 3H).

**$^{13}$C NMR (126 MHz, $CDCl_3$):** δ 177.0, 136.9, 130.2, 82.6, 76.6, 72.9, 56.3, 42.6, 39.9, 37.3, 34.2, 31.8, 25.2, 22.7, 14.1.

**HRMS (*m/z*):** $[M+Na]^+$ calcd for $C_{15}H_{24}O_4Na^+$ 291.1567, found 291.1571.

$[\alpha]_D^{20}$ = −6.6 ($c$ = 1.0 in $CHCl_3$)

Synthesis of **$PGF_{2\alpha}$ (1)**:

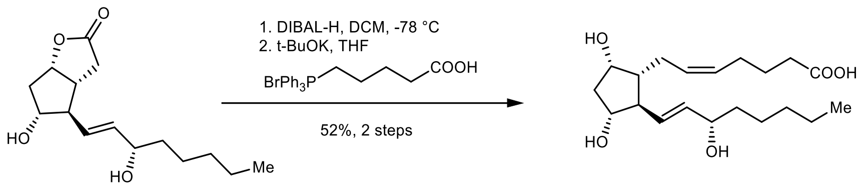


To a stirred solution of compound **7** (15.5 g, 57.8 mmol, 1.0 equiv.) in DCM (1400 mL) was added DIBAL-H (1.0 M in hexane, 231 mL, 231 mmol, 4.0 equiv.) dropwise via syringe at −78 °C. After stirring at the same temperature for 2 hours, the reaction was quenched with MeOH (100 mL) at −78 °C. The mixture was warmed to room temperature and saturated aq. $NH_4Cl$ (60 mL) was added. After stirring at the room temperature for 15 minutes, the mixture was diluted with DCM (500 mL) and then added anhydrous $Na_2SO_4$ (240 g). Followed by stirring at the room temperature for 15 minutes, the mixture was filtered and washed by DCM (5 × 300 mL). The combined organic phases were concentrated in *vacuo* to give colorless oil as crude product, which was directly used in next step without further purification.

To a stirred solution of (4-carboxybutyl)(triphenyl)phosphonium bromide (154 g, 347 mmol, 6.0 equiv.) in THF (1000 mL) was added *t*-BuOK (77.9 g, 694 mmol, 12.0 equiv.) at 0 °C. After stirring at the same temperature for 40 minutes, a solution of above crude hemiacetal in THF (150 mL) was added dropwise via syringe. The reaction mixture was stirred at room temperature for 2 hours, then quenched with $H_2O$ (500 mL) at 0 °C and washed with $Et_2O$ (500 mL). The aqueous phase was acidic with 2 N aq. HCl (380 mL) and extracted with DCM (5 × 1200 mL). The combined organic extracts were dried over anhydrous $Na_2SO_4$, filtered, and concentrated in *vacuo*. The crude material was soaked with EtOAc (1000 mL) and stayed in a sonic bath (3 × 10 minutes) until the solid dispersed, then stayed overnight. The mixture was filtered and washed with EtOAc (6 × 500 mL). The combined organic phase was concentrated in *vacuo* and the crude material was purified by flash column chromatography (60:35:5 EtOAc:petroleum

ether:HOAc) to yield **PGF$_{2\alpha}$ (1)** (10.6 g, 52% yield) as a yellow oil.

**$^1$H NMR (700 MHz, CDCl$_3$):** δ 5.55 (dd, *J* = 15.3, 6.9 Hz, 1H), 5.49 (dd, *J* = 15.2, 8.7 Hz, 1H), 5.46 – 5.39 (m, 1H), 5.38 – 5.32 (m, 1H), 4.21 – 4.13 (m, 1H), 4.08 (ddd, *J* = 6.6, 6.6, 6.6 Hz, 1H), 4.02 – 3.91 (m, 1H), 2.48 – 2.28 (m, 3H), 2.27 – 1.95 (m, 5H), 1.83 – 1.73 (m, 1H), 1.72 – 1.62 (m, 2H), 1.60 – 1.54 (m, 1H), 1.53 – 1.42 (m, 2H), 1.39 – 1.21 (m, 6H), 0.88 (t, *J* = 6.8 Hz, 3H).

**$^{13}$C NMR (176 MHz, CDCl$_3$):** δ 177.9, 135.2, 133.0, 129.7, 129.3, 77.7, 73.3, 72.6, 55.5, 50.3, 42.8, 37.0, 33.3, 31.9, 26.5, 25.4, 24.7, 22.8, 14.2.

**HRMS (*m/z*):** [M–H]$^-$ calcd for $C_{20}H_{33}O_5^-$ 353.2333, found 353.2333.

**$[\alpha]_D^{25}$** = +23.8 (*c* = 1.0 in THF)

Synthesis of compound **20**:

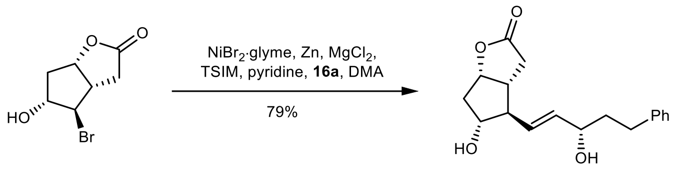


**8** **20**

To a stirred solution of compound **8** (4.00 g, 18.1 mmol, 1.0 equiv.) in DMA (36 mL) was added TSIM (2.9 mL, 19.9 mmol, 1.1 equiv.). After stirring at room temperature for 1 hour, the mixture was added to another flask charged with NiBr$_2$·glyme (838 mg, 2.72 mmol, 0.15 equiv.), MgCl$_2$ (1.72 g, 18.1 mmol, 1.0 equiv.), zinc powder (2.37 g, 36.2 mmol, 2.0 equiv.) and compound **16a** (8.73 g, 36.2 mmol, 2.0 equiv.) via syringe. Followed by adding DMA (84 mL) and pyridine (1.5 mL, 18.1 mmol, 1.0 equiv.), the mixture stirred at room temperature for 20 hours. After cooling to 0 °C, the mixture was added 1 N aq. HCl (120 mL) and stir at room temperature for 15 minutes. Then the mixture was extracted with EtOAc (5 × 240 mL), and the combined organic extracts were dried over anhydrous Na$_2$SO$_4$, filtered, and concentrated in *vacuo* (*vacuubrand*® RZ 6). After removing most of DMA, the concentrated mixture was diluted with EtOAc (400 mL) and washed with 10% aq. NaCl (3 × 80 mL), the organic phase was dried over anhydrous

$Na_2SO_4$, filtered, and concentrated in *vacuo*. The crude material was purified by flash column chromatography (1:1 petroleum ether:acetone) to yield compound **20** (4.32 g, 79% yield) as a pale yellow oil.

**$^1$H NMR (500 MHz, $CDCl_3$):** δ 7.31 – 7.26 (m, 2H), 7.22 – 7.16 (m, 3H), 5.65 (dd, *J* = 15.4, 6.6 Hz, 1H), 5.48 (dd, *J* = 15.4, 8.3 Hz, 1H), 4.90 (ddd, *J* = 7.1, 7.1, 3.0 Hz, 1H), 4.11 (ddd, *J* = 6.6, 6.5, 6.5 Hz, 1H), 3.96 (ddd, *J* = 7.3, 7.3, 7.3 Hz, 1H), 2.78 – 2.62 (m, 3H), 2.61 – 2.47 (m, 2H), 2.43 (dd, *J* = 18.1, 1.6 Hz, 1H), 2.34 – 2.26 (m, 1H), 1.95 (ddd, *J* = 14.8, 7.6, 3.0 Hz, 1H), 1.92 – 1.78 (m, 2H).

**$^{13}$C NMR (126 MHz, $CDCl_3$):** δ 177.0, 141.7, 136.6, 130.4, 128.6, 128.6, 126.1, 82.6, 76.6, 72.1, 56.3, 42.6, 39.9, 38.8, 34.3, 31.9.

**HRMS (*m/z*):** $[M+Na]^+$ calcd for $C_{18}H_{22}O_4Na^+$ 325.1410, found 325.1414.

$[\alpha]_D^{20}$ = −5.6 (*c* = 1.0 in $CHCl_3$)

Synthesis of **Bimatoprost (2)**:

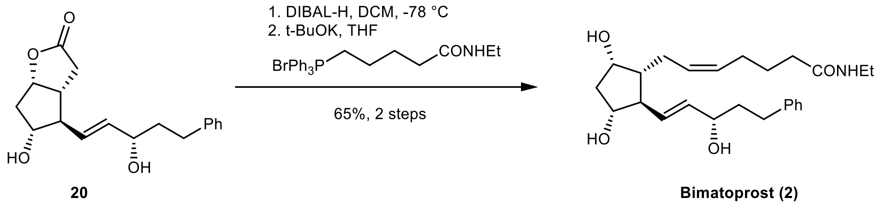


To a stirred solution of compound **20** (2.10 g, 6.95 mmol, 1.0 equiv.) in DCM (174 mL) was added DIBAL-H (1.0 M in hexane, 27.8 mL, 27.8 mmol, 4.0 equiv.) dropwise via syringe at −78 °C. After stirring at the same temperature for 2 hours, the reaction was quenched with MeOH (20 ml) at −78 °C. The mixture was warmed to room temperature and saturated aq. $NH_4Cl$ (10 mL) was added. After stirring at the room temperature for 15 minutes, the mixture was diluted with DCM (100 mL) and then added anhydrous $Na_2SO_4$ (40 g). Followed by stirring at the room temperature for 15 minutes, the mixture was filtered and washed by DCM (5 × 50 mL). The combined organic phase was concentrated in *vacuo* to give colorless oil as crude product, which was directly used in next step without further purification.

To a stirred solution of (5-(ethylamino)-5-oxopentyl)triphenylphosphonium bromide[2] (19.6 g, 41.7 mmol, 6.0 equiv.) in THF (120 mL) was added *t*-BuOK (9.36 g, 83.4 mmol, 12.0 equiv.) at 0 °C. After stirring at the same temperature for 40 minutes, a solution of above crude hemiacetal in THF (20 mL) was added dropwise via syringe. The reaction mixture was stirred at room temperature for 2 hours, then quenched with saturated aq. $NH_4Cl$ (80 mL) and extracted with EtOAc (5 × 100 mL). The combined organic extracts were dried over anhydrous $Na_2SO_4$, filtered, and concentrated in *vacuo*. The crude material was soaked with EtOAc (200 mL) and stayed in a sonic bath (3 × 5 minutes) until the solid dispersed, then stayed overnight. The mixture was filtered and washed with EtOAc (6 × 100 mL). The combined organic phases were concentrated in *vacuo* and the crude material was purified by flash column chromatography (20:1 EtOAc:MeOH) to yield **Bimatoprost (2)** (1.89 g, 65% yield) as a yellow oil.

**$^1$H NMR (500 MHz, $CDCl_3$):** δ 7.30 – 7.23 (m, 2H), 7.21 – 7.14 (m, 3H), 5.96 – 5.79 (m, 1H), 5.59 (dd, *J* = 15.3, 7.1 Hz, 1H), 5.49 (dd, *J* = 15.2, 8.8 Hz, 1H), 5.44 – 5.29 (m, 2H), 4.16 – 4.11 (m, 1H), 4.11 – 4.04 (m, 1H), 3.97 – 3.88 (m, 1H), 3.50 – 3.30 (m, 3H), 3.28 – 3.18 (m, 2H), 2.75 – 2.60 (m, 2H), 2.39 – 2.25 (m, 2H), 2.23 – 1.96 (m, 6H), 1.95 – 1.85 (m, 1H), 1.84 – 1.73 (m, 2H), 1.72 – 1.58 (m, 2H), 1.52 – 1.41 (m, 1H), 1.10 (t, *J* = 7.3 Hz, 3H).

**$^{13}$C NMR (126 MHz, $CDCl_3$):** δ 173.5, 142.2, 135.1, 133.2, 129.9, 129.3, 128.6, 128.5, 125.9, 78.1, 72.8, 72.3, 55.8, 50.6, 43.0, 38.9, 36.0, 34.5, 32.0, 26.8, 25.8, 25.6, 14.9.

**HRMS (*m/z*):** $[M+Na]^+$ calcd for $C_{25}H_{37}NO_4Na^+$ 438.2615, found 438.2615.

$[\alpha]_D^{22}$ = +32.0 (*c* = 1.0 in MeOH).

Synthesis of compound **S25**:

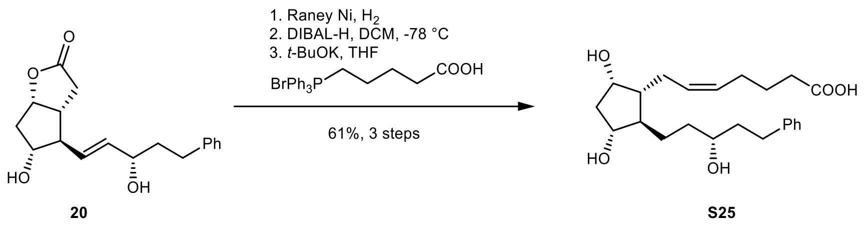


To a stirred solution of compound **20** (2.21 g, 7.31 mmol, 1.0 equiv.) in THF (100 mL) was added Raney Ni (washed by THF, 2.20 g). The reaction mixture was degassed and refilled three times with $H_2$, then the mixture was stirred under $H_2$ (1 atm) at room temperature for 8 hours. The mixture was filtered and washed by DCM (3 ×50 mL). The combined organic phase was concentrated in *vacuo* to give colorless oil as product, which was directly used in next step without further purification.

To a stirred solution of above product in DCM (183 mL) was added DIBAL-H (1.0 M in hexane, 29.2 mL, 29.2 mmol, 4.0 equiv.) dropwise via syringe at −78 °C. After stirring at the same temperature for 2 hours, the reaction was quenched with MeOH (20 mL) at −78 °C. The mixture was warmed to room temperature and saturated aq. $NH_4Cl$ (10 mL) was added. After stirring at the room temperature for 15 minutes, the mixture was diluted with DCM (100 mL) and then added anhydrous $Na_2SO_4$ (40 g). Followed by stirring at the room temperature for 15 minutes, the mixture was filtered and washed by DCM (5 × 50 mL). The combined organic phases were concentrated in *vacuo* to give colorless oil as crude product, which was directly used in next step without further purification.

To a stirred solution of (4-carboxybutyl)(triphenyl)phosphonium bromide (19.5 g, 43.9 mmol, 6.0 equiv.) in THF (120 mL) was added *t*-BuOK (9.84 g, 87.7 mmol, 12.0 equiv.) at 0 °C. After stirring at the same temperature for 40 minutes, a solution of above crude hemiacetal in THF (26 mL) was added dropwise via syringe. The reaction mixture was stirred at room temperature for 2 hours, then then quenched with $H_2O$ (100 mL) at 0 °C and washed with $Et_2O$ (100 mL). The aqueous phase was acidic with 2 N aq. HCl (50 mL) and extracted with DCM (5 × 150 mL). The combined organic extracts were dried over anhydrous $Na_2SO_4$, filtered, and concentrated in *vacuo*. The crude material was soaked with EtOAc (200 mL) and hexane (100 mL) stayed in a sonic bath (3 × 5 minutes) until the solid dispersed, then stayed overnight.

The mixture was filtered and washed with EtOAc (6 × 50 mL). The combined organic phases were concentrated in *vacuo* and the crude material was purified by flash column chromatography (60:35:5 EtOAc:petroleum ether:HOAc) to yield compound **S25** (1.74 g, 61% yield) as a pale yellow oil.

**$^{1}$H NMR (500 MHz, $CDCl_3$):** δ 7.30 – 7.24 (m, 2H), 7.22 – 7.13 (m, 3H), 5.52 – 5.31 (m, 2H), 4.23 – 4.08 (m, 1H), 3.99 – 3.90 (m, 1H), 3.73 – 3.61 (m, 1H), 2.79 (dt, *J* = 14.5, 7.6 Hz, 1H), 2.65 (dt, *J* = 14.0, 8.2 Hz, 1H), 2.39 – 2.03 (m, 6H), 1.93 – 1.45 (m, 10H), 1.43 – 1.28 (m, 2H).

**$^{13}$C NMR (126 MHz, $CDCl_3$):** δ 177.6, 142.2, 129.6, 129.6, 128.6, 126.0, 78.7, 74.5, 71.7, 52.4, 51.8, 42.6, 38.8, 35.3, 33.2, 32.2, 29.1, 26.7, 26.5, 24.7.

**HRMS (*m/z*):** $[M+Na]^+$ calcd for $C_{23}H_{34}O_5Na^+$ 413.2298, found 413.2297.

**$[\alpha]_D^{20}$** = +30.3 (*c* = 1.0 in MeOH).

Synthesis of **Latanoprost 25**:

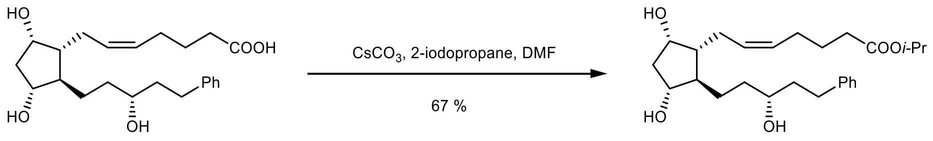


**S25** **Latanoprost (25)**

To a stirred solution of compound **S25** (1.74 g, 4.46 mmol, 1.0 equiv.) in DMF (30 mL) was added $Cs_2CO_3$ (2.18 g, 6.69 mmol, 1.5 equiv.) and 2-iodopropane (890 μl, 8.92 mmol, 2.0 equiv.) at room temperature. After stirring at the same temperature for 20 hours, the reaction mixture was poured into 3% aq. citric acid (150 mL) and extracted with $Et_2O$ (5 × 150 mL). The combined organic phases were washed with 10% aq. $NaHCO_3$ (100 mL) and brine (2 × 100 mL). Followed by drying over anhydrous $Na_2SO_4$, filtering, and concentrating in *vacuo*, the combined organic phases were concentrated in *vacuo*. The crude material was purified by flash column chromatography (2:1 EtOAc:petroleum ether) to yield **Latanoprost 25** (1.29 g, 67% yield) as a colorless oil.

**$^1$H NMR (500 MHz, CDCl$_3$):** δ 7.31 – 7.25 (m, 2H), 7.22 – 7.16 (m, 3H), 5.52 – 5.34 (m, 2H), 5.06 – 4.95 (m, 1H), 4.24 – 4.11 (m, 1H), 3.99 – 3.90 (m, 1H), 3.71 – 3.63 (m, 1H), 2.85 – 2.75 (m, 1H), 2.72 – 2.62 (m, 1H), 2.37 – 2.18 (m, 7H), 2.17 – 2.07 (m, 2H), 1.90 – 1.85 (m, 2H), 1.83 – 1.74 (m, 2H), 1.73 – 1.65 (m, 3H), 1.65 – 1.58 (m, 2H), 1.56 – 1.48 (m, 1H), 1.44 – 1.30 (m, 2H), 1.22 (d, $J$ = 6.3 Hz, 6H).

**$^{13}$C NMR (126 MHz, CDCl$_3$):** δ 173.6, 142.2, 129.7, 129.5, 128.5, 128.5, 125.9, 78.9, 74.7, 71.4, 67.8, 52.9, 52.0, 42.6, 39.2, 35.9, 34.2, 32.2, 29.7, 27.0, 26.7, 25.1, 22.0.

**HRMS (*m/z*):** [M+Na]$^+$ calcd for $C_{26}H_{40}O_5Na^+$ 455.2768, found 455.2766.

**$[\alpha]_D^{20}$** =+33.8 ($c$ = 1.0 in $CH_3CN$).

Synthesis of compound **21**:

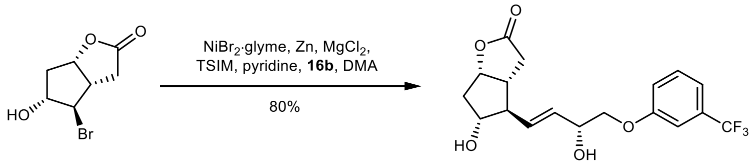


8 21

To a stirred solution of compound **8** (2.00 g, 9.05 mmol, 1.0 equiv.) in DMA (18 mL) was added TSIM (1.5 mL, 9.96 mmol, 1.1 equiv.). After stirring at room temperature for 1 hour, the mixture was added to another flask charged with $NiBr_2$·glyme (419 mg, 1.36 mmol, 0.15 equiv.), $MgCl_2$ (862 mg, 9.05 mmol, 1.0 equiv.), zinc powder (1.18 g, 18.1 mmol, 2.0 equiv.) and compound **16b** (5.63 g, 18.1 mmol, 2.0 equiv.) via syringe. Followed by adding DMA (42 mL) and pyridine (732 μL, 9.05 mmol, 1.0 equiv.), the mixture stirred at room temperature for 20 hours. After cooling to 0 °C, the mixture was added 1 N aq. HCl (50 mL) and stir at room temperature for 15 minutes. Then the mixture was extracted with EtOAc (5 × 100 mL), and the combined organic extracts were dried over anhydrous $Na_2SO_4$, filtered, and concentrated in *vacuo* (*vacuubrand*® RZ 6). After removing most of DMA, the concentrated mixture was diluted with EtOAc (200 mL) and washed with 10% aq. NaCl (3 × 40 mL), the organic phase was dried over anhydrous $Na_2SO_4$, filtered, and concentrated in *vacuo* again. The crude material was purified by flash column

chromatography (1:1 petroleum ether:acetone) to yield compound **21** (2.71 g, 80% yield) as a white solid. CCDC 2319091 contains the supplementary crystallographic data of compound **21**. Crystallographic data for the structures reported in this Article have been deposited at the Cambridge Crystallographic Data Centre, under deposition numbers CCDC 2319091 (**21**). Copies of the data can be obtained free of charge via https://www.ccdc.cam.ac.uk/structures/.

**$^{1}$H NMR (500 MHz, $CDCl_3$):** δ 7.42 – 7.36 (m, 1H), 7.25 – 7.21 (m, 1H), 7.15 – 7.11 (m, 1H), 7.09 – 7.05 (m, 1H), 5.75 – 5.64 (m, 2H), 4.89 (ddd, *J* = 7.0, 7.0, 3.0 Hz, 1H), 4.55 – 4.47 (m, 1H), 4.05 – 3.89 (m, 3H), 3.27 – 3.19 (m, 2H), 2.72 (dd, *J* = 18.1, 9.6 Hz, 1H), 2.64 – 2.56 (m, 1H), 2.51 (dt, *J* = 14.4, 6.9 Hz, 1H), 2.42 (d, *J* = 18.1 Hz, 1H), 2.35 – 2.29 (m, 1H), 1.94 (ddd, *J* = 14.6, 7.7, 2.7 Hz, 1H).

**$^{13}$C NMR (126 MHz, $CDCl_3$):** δ 177.3, 158.6, 133.2, 131.9 (q, *J* = 32.3 Hz), 131.3, 130.3, 124.0 (q, *J* = 272.5 Hz), 118.1, 118.0 (q, *J* = 3.9 Hz), 111.5 (q, *J* = 3.8 Hz), 82.7, 76.4, 71.8, 70.8, 56.3, 42.4, 39.7, 34.3.

**$^{19}$F NMR (471 MHz, $CDCl_3$):** δ -62.6.

**HRMS (*m/z*):** $[M+Na]^+$ calcd for $C_{18}H_{19}O_5F_3Na^+$ 395.1077, found 395.1075.

**$[\alpha]_D^{20}$** = −4.3 (*c* = 1.0 in THF).

**[m.p.]** = 112–114 °C

Synthesis of **Fluprostenol (3)**:

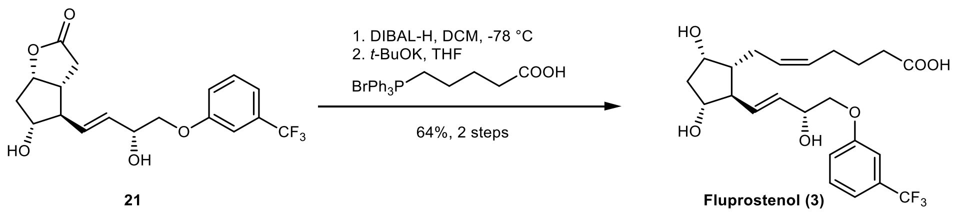


To a stirred solution of compound **21** (2.71 g, 7.28 mmol, 1.0 equiv.) in DCM (182 mL) was added DIBAL-H (1.0 M in hexane, 29.1 mL, 29.1 mmol, 4.0 equiv.) dropwise via syringe at −78 °C. After stirring at the same temperature for 2 hours, the reaction was quenched with MeOH (20 mL) at −78 °C.

The mixture was warmed to room temperature and saturated aq. $NH_4Cl$ (10 mL) was added. After stirring at the room temperature for 15 minutes, the mixture was diluted with DCM (100 mL) and then added anhydrous $Na_2SO_4$ (40 g). Followed by stirring at the room temperature for 15 minutes, the mixture was filtered and washed by DCM (5 × 50 mL). The combined organic phases were concentrated in *vacuo* to give colorless oil as crude product, which was directly used in next step without further purification.

To a stirred solution of (4-carboxybutyl)(triphenyl)phosphonium bromide (19.4 g, 43.7 mmol, 6.0 equiv.) in THF (120 mL) was added *t*-BuOK (9.81 g, 87.4 mmol, 12.0 equiv.) at 0 °C. After stirring at the same temperature for 40 minutes, a solution of above crude hemiacetal in THF (25 mL) was added dropwise via syringe. The reaction mixture was stirred at room temperature for 2 hours, then then quenched with $H_2O$ (100 mL) at 0 °C and washed with $Et_2O$ (100 mL). The aqueous phase was acidic with 2 N aq. HCl (50 mL) and extracted with DCM (5 × 150 mL). The combined organic extracts were dried over anhydrous $Na_2SO_4$, filtered, and concentrated in *vacuo*. The crude material was soaked with EtOAc (200 mL) and stayed in a sonic bath (3 × 10 minutes) until the solid dispersed, then stayed overnight. The mixture was filtered and washed with EtOAc (6 × 50 mL). The combined organic phases were concentrated in *vacuo* and the crude material was purified by flash column chromatography (60:35:5 EtOAc:petroleum ether:HOAc) to yield **Fluprostenol (3)** (2.13g, 64% yield) as a yellow oil.

**$^1$H NMR (500 MHz, $CDCl_3$):** δ 7.41 – 7.34 (m, 1H), 7.24 – 7.19 (m, 1H), 7.16 – 7.12 (m, 1H), 7.10 – 7.05 (m, 1H), 5.77 – 5.63 (m, 2H), 5.49 – 5.40 (m, 1H), 5.38 – 5.29 (m, 1H), 4.61 – 4.53 (m, 1H), 4.23 – 4.15 (m, 1H), 4.05 – 3.91 (m, 3H), 2.45 – 2.35 (m, 1H), 2.34 – 1.99 (m, 7H), 1.83 – 1.73 (m, 1H), 1.72 – 1.59 (m, 2H), 1.56 – 1.46 (m, 1H).

**$^{13}$C NMR (126 MHz, $CDCl_3$):** δ 178.0, 158.8, 135.5, 132.0 (q, $J$ = 32.4 Hz), 130.2, 129.8, 129.8, 129.1, 124.0 (q, $J$ = 272.4 Hz), 118.2, 118.0 (q, $J$ = 3.6 Hz), 111.6 (q, $J$ = 3.8 Hz), 77.7, 72.8, 71.9, 71.0, 55.7, 50.4, 42.9, 33.1, 26.4, 25.4, 24.6.

**$^{19}$F NMR (471 MHz, $CDCl_3$):** δ -62.7.

**HRMS (*m/z*):** $[M+Na]^+$ calcd for $C_{23}H_{29}O_6F_3Na^+$ 481.1808, found 481.1809.

$[\alpha]_D^{20}$ = +20.8 ($c$ = 1.0 in $CHCl_3$).

Synthesis of compound **22**:

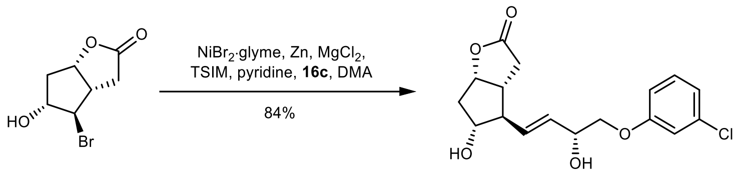

**8** **22**

To a stirred solution of compound **8** (2.00 g, 9.05 mmol, 1.0 equiv.) in DMA (18 mL) was added TSIM (1.5 mL, 9.96 mmol, 1.1 equiv.). After stirring at room temperature for 1 hour, the mixture was added to another flask charged with $NiBr_2$·glyme (419 mg, 1.36 mmol, 0.15 equiv.), $MgCl_2$ (862 mg, 9.05 mmol, 1.0 equiv.), zinc powder (1.18 g, 18.1 mmol, 2.0 equiv.) and compound **16c** (5.02 g, 18.1 mmol, 2.0 equiv.) via syringe. Followed by adding DMA (42 mL) and pyridine (732 μL, 9.05 mmol, 1.0 equiv.), the mixture stirred at room temperature for 20 hours. After cooling to 0 °C, the mixture was added 1N aq. HCl (50 mL) and stir at room temperature for 15 minutes. Then the mixture was extracted with EtOAc (5 × 100 mL), and the combined organic extracts were dried over anhydrous $Na_2SO_4$, filtered, and concentrated in *vacuo* (*vacuubrand*® RZ 6). After removing most of DMA, the concentrated mixture was diluted with EtOAc (200 mL) and washed with 10% aq. NaCl (3 × 40 mL), the organic phase was dried over anhydrous $Na_2SO_4$, filtered, and concentrated in *vacuo*. The crude material was purified by flash column chromatography (1:1 petroleum ether:acetone) to yield compound **22** (2.57 g, 84% yield) as a white solid.

**$^1$H NMR (500 MHz, $CDCl_3$):** δ 7.24 – 7.19 (m, 1H), 6.99 – 6.95 (m, 1H), 6.93 – 6.90 (m, 1H), 6.82 – 6.78 (m, 1H), 5.77 – 5.67 (m, 2H), 4.93 (ddd, *J* = 7.0, 7.0, 3.0 Hz, 1H), 4.57 – 4.50 (m, 1H), 4.06 – 3.97 (m, 2H), 3.87 (dd, *J* = 9.3, 7.6 Hz, 1H), 2.75 (dd, *J* = 18.1, 9.7 Hz, 1H), 2.68 – 2.60 (m, 1H), 2.57 – 2.49 (m, 1H), 2.46 (dd, *J* = 18.1, 1.8 Hz, 1H), 2.40 – 2.34 (m, 1H), 1.99 (ddd, *J* = 14.8, 7.4, 3.0 Hz, 1H).

**$^{13}$C NMR (126 MHz, $CDCl_3$):** δ 176.7, 159.1, 135.2, 132.3, 131.1, 130.5, 121.8, 115.2, 113.2, 82.5, 76.8, 72.0, 70.5, 56.5, 42.7, 40.1, 34.4.

**HRMS (*m/z*):** $[M–H]^-$ calcd for $C_{17}H_{18}O_5Cl^-$ 337.0848, found 337.0842.

$[\alpha]_D^{20}$ = −4.2 ($c$ = 1.0 in THF).

Synthesis of **Cloprostenol (4)**:

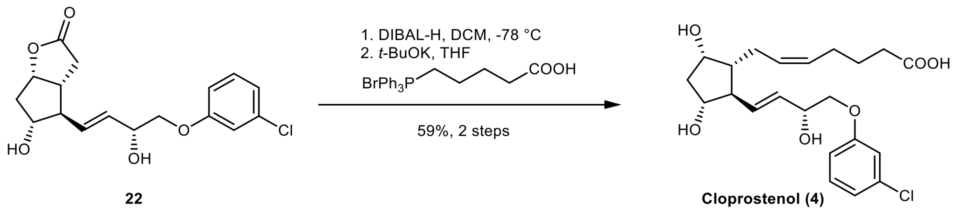


**22**

**Cloprostenol (4)**

To a stirred solution of compound **22** (2.44 g, 7.22 mmol, 1.0 equiv.) in DCM (182 mL) was added DIBAL-H (1.0 M in hexane, 28.8 mL, 28.8 mmol, 4.0 equiv.) dropwise via syringe at −78 °C. After stirring at the same temperature for 2 hours, the reaction was quenched with MeOH (10 mL) at −78 °C. The mixture was warmed to room temperature and saturated aq. $NH_4Cl$ (10 mL) was added. After stirring at the room temperature for 15 minutes, the mixture was diluted with DCM (100 mL) and then added anhydrous $Na_2SO_4$ (40 g). Followed by stirring at the room temperature for 15 minutes, the mixture was filtered and washed by DCM (5 × 50 mL). The combined organic phases were concentrated in *vacuo* to give colorless oil as crude product, which was directly used in next step without further purification.

To a stirred solution of (4-carboxybutyl)(triphenyl)phosphonium bromide (19.2 g, 43.3 mmol, 6.0 equiv.) in THF (120 mL) was added *t*-BuOK (9.72 g, 86.6 mmol, 12.0 equiv.) at 0 °C. After stirring at the same temperature for 40 minutes, a solution of above crude hemiacetal in THF (25 mL) was added dropwise via syringe. The reaction mixture was stirred at room temperature for 2 hours, then then quenched with $H_2O$ (100 mL) at 0 °C and washed with $Et_2O$ (100 mL). The aqueous phase was acidic with 2 N aq. HCl (50 mL) and extracted with DCM (5 × 150 mL). The combined organic extracts were dried over anhydrous $Na_2SO_4$, filtered, and concentrated in *vacuo*. The crude material was soaked with EtOAc (200 mL) and stayed in a sonic bath (3 × 10 minutes) until the solid dispersed, then stayed overnight. The mixture was filtered and washed with EtOAc (6 × 50 mL). The combined organic phases were concentrated in *vacuo* and the crude material was purified by flash column chromatography (60:35:5 EtOAc:petroleum

ether:HOAc) to yield **Cloprostenol (4)** (1.82 g, 59% yield) as a yellow oil.

**$^1$H NMR (500 MHz, $CDCl_3$):** δ 7.21 – 7.15 (m, 1H), 6.95 – 6.92 (m, 1H), 6.92 – 6.88 (m, 1H), 6.82 – 6.77 (m, 1H), 5.71 (dd, *J* = 15.4, 8.3 Hz, 1H), 5.65 (dd, *J* = 15.4, 6.0 Hz, 1H), 5.49 – 5.40 (m, 1H), 5.38 – 5.29 (m, 1H), 4.58 – 4.51 (m, 1H), 4.23 – 4.13 (m, 1H), 4.03 – 3.85 (m, 3H), 2.42 – 2.34 (m, 1H), 2.32 (t, *J* = 6.6 Hz, 2H), 2.26 – 2.05 (m, 5H), 1.83 – 1.73 (m, 1H), 1.72 – 1.58 (m, 2H), 1.55 – 1.45 (m, 1H).

**$^{13}$C NMR (126 MHz, $CDCl_3$):** δ 177.7, 159.3, 135.2, 135.0, 130.5, 129.8, 129.6, 129.2, 121.5, 115.2, 113.2, 77.8, 72.8, 71.9, 71.0, 55.8, 50.5, 42.9, 33.0, 26.4, 25.4, 24.5.

**HRMS (*m/z*)**: $[M–H]^-$ calcd for $C_{22}H_{28}O_6Cl^-$ 423.1580, found 423.1572.

$[\alpha]_D^{20}$ = +23.7 (c = 1.0 in CHCl3).

Synthesis of compound **23**:

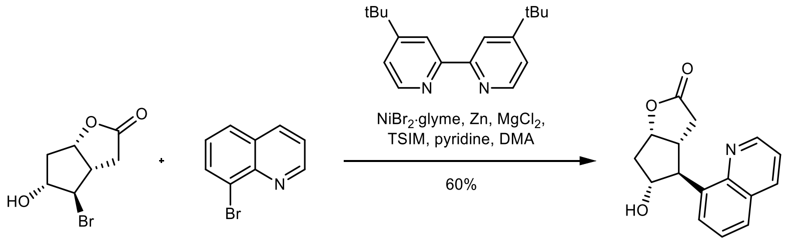


**8** **S23** **23**

To a stirred solution of compound **8** (66.3 mg, 0.3 mmol, 1.0 equiv.) in DMA (0.6 mL) was added TSIM (48 μL, 0.33 mmol, 1.1 equiv.). After stirring at room temperature for 1 hour, the mixture was added to another flask charged with $NiBr_2$·glyme (13.9 mg, 0.045 mmol, 0.15 equiv.), $MgCl_2$ (28.6 mg, 0.3 mmol, 1.0 equiv.), zinc powder (39.5 mg, 0.6 mmol, 2.0 equiv.), 4,4'-di-*t*BuBIPY[3] (12.1 mg, 0.045 mmol, 0.15 equiv.) and compound **S23** (124 mg, 0.6 mmol, 2.0 equiv.) via syringe. Followed by adding DMA (1.4 mL) and pyridine (24 μL, 0.3 mmol, 1.0 equiv.), the mixture stirred at room temperature for 20 hours. After cooling to 0 °C, the mixture was added 1N aq. HCl (2 mL) and stir at room temperature for 15 minutes. Then the mixture was added 25% aq. $NH_3$ (1 mL), saturated aq. NaCl (1 mL) and extracted with EtOAc (5 × 10 mL), and the combined organic extracts were dried over anhydrous $Na_2SO_4$, filtered, and

concentrated in *vacuo* (*vacuubrand*® RZ 6). After removing most of DMA, the concentrated mixture was diluted with EtOAc (25 mL) and washed with 10% aq. NaCl (3 × 5 mL), the organic phase was dried over anhydrous $Na_2SO_4$, filtered, and concentrated in *vacuo*. The crude material was purified by flash column chromatography (1:1 petroleum ether:acetone) to yield compound **23** (48.6 mg, 60% yield) as a white solid.

**$^1$H NMR (400 MHz, $CDCl_3$):** δ 8.96 – 8.85 (m, 1H), 8.31 – 8.21 (m, 1H), 7.85 – 7.75 (m, 1H), 7.62 – 7.45 (m, 3H), 5.09 (ddd, *J* = 7.1, 7.1, 3.3 Hz, 1H), 4.35 (ddd, *J* = 7.6, 7.6, 7.6 Hz, 1H), 4.20 – 4.10 (m, 1H), 3.49 – 3.39 (m, 1H), 2.89 – 2.73 (m, 2H), 2.55 – 2.45 (m, 1H), 2.26 (ddd, *J* = 14.8, 7.6, 3.3 Hz, 1H).

**$^{13}$C NMR (101 MHz, $CDCl_3$):** δ 176.9, 149.3, 147.1, 137.6, 137.4, 129.1, 127.5, 127.0, 126.8, 121.5, 82.9, 78.5, 53.6, 42.1, 41.4, 35.0.

**HRMS (*m/z*):** $[M+H]^+$ calcd for $C_{16}H_{16}NO_3^+$ 270.1125, found 270.1129.

**$[\alpha]_D^{20}$** = +51.2 (*c* = 1.0 in $CHCl_3$).

Synthesis of compound **24**:

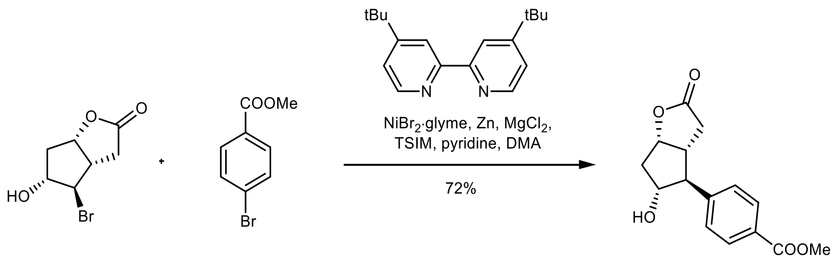


**8** **S24** **24**

To a stirred solution of compound **8** (66.3 mg, 0.3 mmol, 1.0 equiv.) in DMA (0.6 mL) was added TSIM (48 μL, 0.33 mmol, 1.1 equiv.). After stirring at room temperature for 1 hour, the mixture was added to another flask charged with $NiBr_2$·glyme (13.9 mg, 0.045 mmol, 0.15 equiv.), $MgCl_2$ (28.6 mg, 0.3 mmol, 1.0 equiv.), zinc powder (39.5 mg, 0.6 mmol, 2.0 equiv.), 4,4'-di-*t*BuBIPY[3] (12.1 mg, 0.045 mmol, 0.15 equiv.) and compound **S24** (129 mg, 0.6 mmol, 2.0 equiv.) via syringe. Followed by adding DMA (1.4 mL) and pyridine (24 μL, 0.3 mmol, 1.0 equiv.), the mixture stirred at room temperature for 20 hours. After cooling to 0 °C, the mixture was added 1N aq. HCl (2 mL) and stir at room temperature for 15 minutes. Then the mixture was added saturated aq. NaCl (1 mL) and extracted with EtOAc (5 × 10 mL), and the combined organic extracts were dried over anhydrous $Na_2SO_4$, filtered, and concentrated in *vacuo* (*vacuubrand*® RZ 6). After removing most of DMA, the concentrated mixture was diluted with EtOAc (25 mL) and washed with 10% aq. NaCl (3 × 5 mL), the organic phase was dried over anhydrous $Na_2SO_4$, filtered, and concentrated in *vacuo*. The crude material was purified by flash column chromatography (1:1 petroleum ether:acetone) to yield compound **24** (59.6 mg, 72% yield) as a white solid.

**$^1$H NMR (400 MHz, $CDCl_3$):** δ 8.06 – 7.99 (m, 2H), 7.32 – 7.27 (m, 2H), 5.06 (ddd, *J* = 6.9, 6.8, 3.2 Hz, 1H), 4.30 (ddd, *J* = 7.4, 7.3, 7.3 Hz, 1H), 3.92 (s, 3H), 3.06 – 2.92 (m, 2H), 2.76 (dd, *J* = 18.3, 9.1 Hz, 1H), 2.66 (ddd, *J* = 14.5, 7.0, 7.0 Hz, 1H), 2.49 (dd, *J* = 18.3, 1.6 Hz, 1H), 2.09 (ddd, *J* = 14.9, 7.6, 3.2 Hz, 1H).

**$^{13}$C NMR (101 MHz, $CDCl_3$):** δ 176.6, 166.8, 144.7, 130.4, 129.6, 127.8, 82.4, 78.6, 59.1, 52.4, 44.0, 40.3, 34.6.

**HRMS (*m/z*):** $[M+Na]^+$ calcd for $C_{15}H_{16}O_5Na^+$ 299.0890, found 299.0896.

$[\alpha]_D^{20}$ = −9.3 ($c$ = 1.0 in $CHCl_3$).

**HPLC Traces and GC Traces for Measuring Enantiomeric Excess:**

The racemic and optical compound **8** were analyzed by HPLC (CHIRALPAK AD-H column, hexane：*i*-PrOH 70:30, 0.7 mL/min) to determine the retention times and enantiomeric excess.

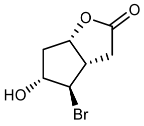


**(±) 8**

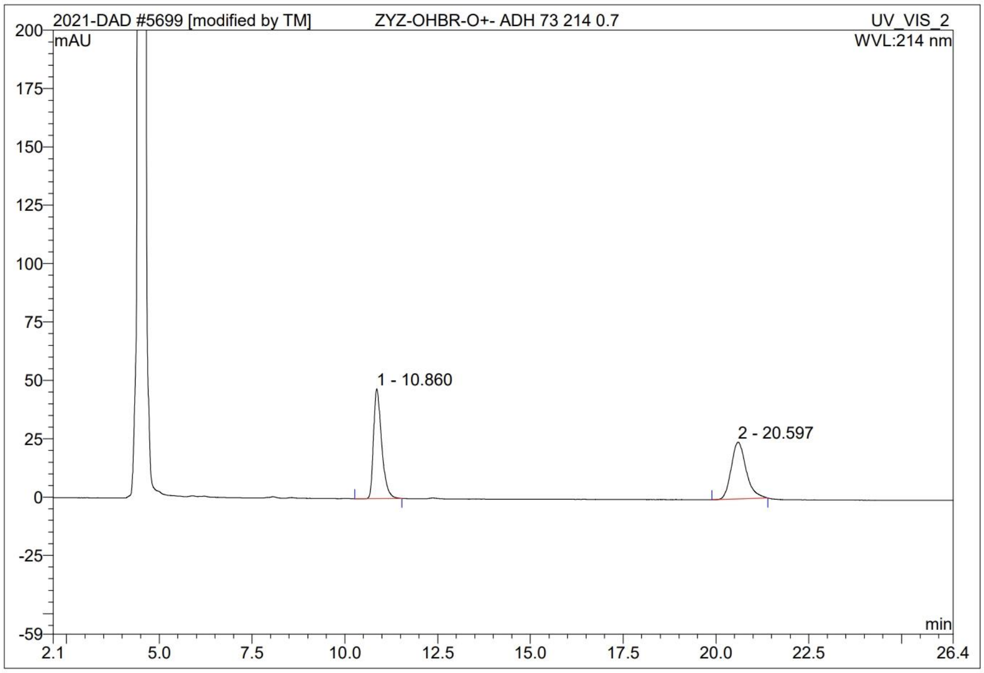


| No. | Ret.Time min | Peak Name | Height mAU | Area mAU*min | Rel.Area % | Amount | Type |
|---|---|---|---|---|---|---|---|
| 1 | 10.86 | n.a. | 46.922 | 11.898 | 51.26 | n.a. | BMB* |
| 2 | 20.60 | n.a. | 24.282 | 11.314 | 48.74 | n.a. | BMB* |
| Total: | | | 71.204 | 23.212 | 100.00 | 0.000 | |

**Supplementary Figure 7.** HPLC traces of racemic compound **8.**

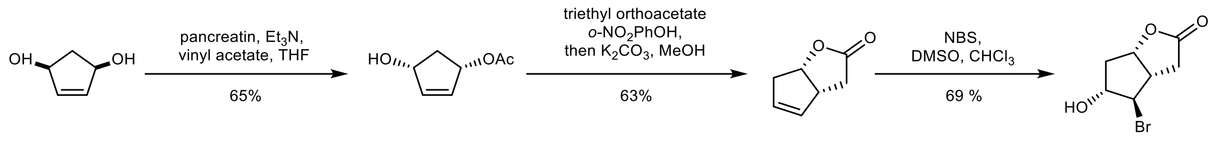


**(±)-12** **11** **9** **8**

Compound **8** prepared from compound **(±)-12**, ee 94.5%.

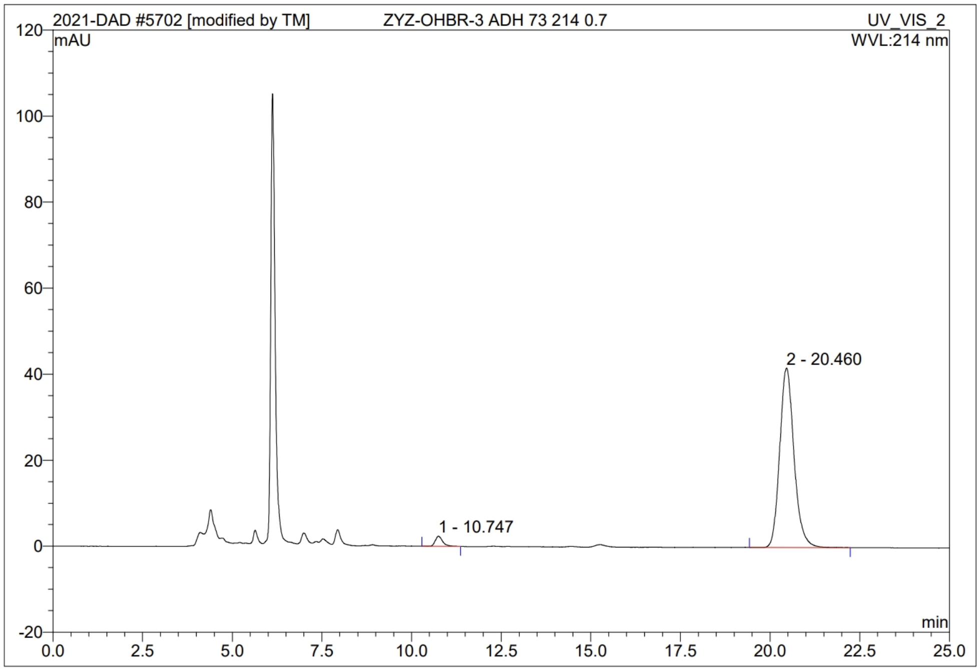


| No. | Ret.Time min | Peak Name | Height mAU | Area mAU*min | Rel.Area % | Amount | Type |
|---|---|---|---|---|---|---|---|
| 1 | 10.75 | n.a. | 2.344 | 0.546 | 2.75 | n.a. | BMB* |
| 2 | 20.46 | n.a. | 41.738 | 19.329 | 97.25 | n.a. | BMB* |
| Total: | | | 44.081 | 19.875 | 100.00 | 0.000 | |

**Supplementary Figure 8.** HPLC traces of optical compound **8** from compound **(±)-12.**

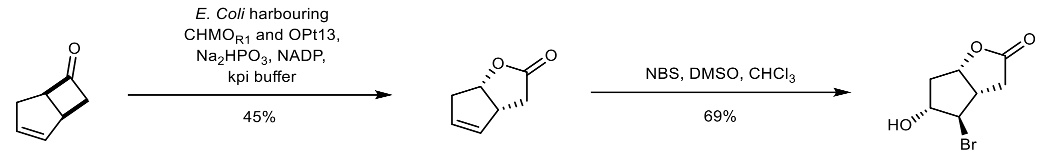


Compound **8** prepared from compound **(±)-10**, ee 95.0%.

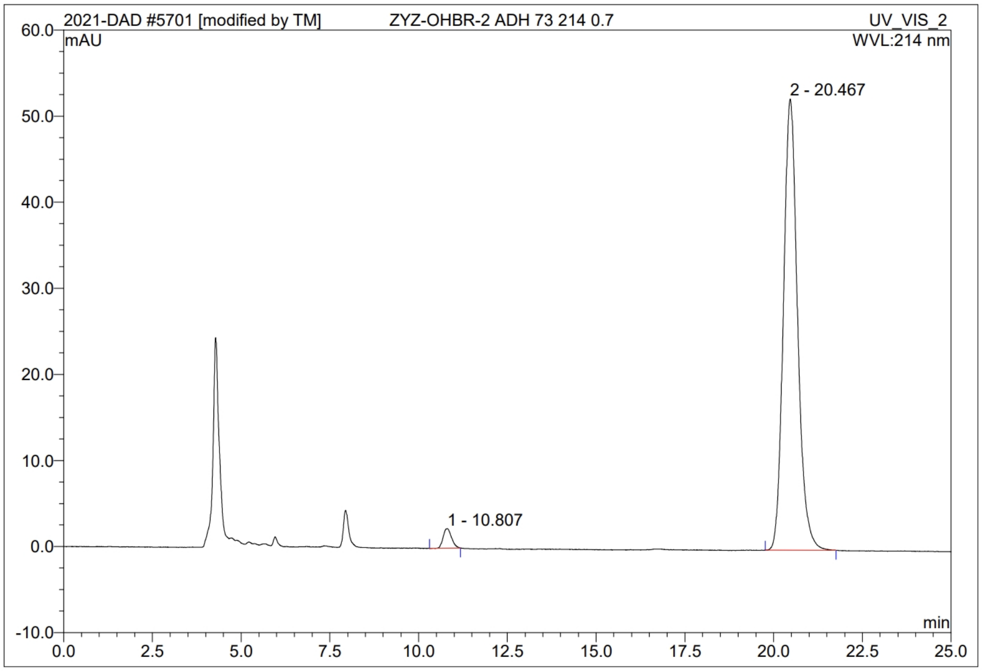


| No. | Ret.Time min | Peak Name | Height mAU | Area mAU*min | Rel.Area % | Amount | Type |
|---|---|---|---|---|---|---|---|
| 1 | 10.81 | n.a. | 2.261 | 0.621 | 2.50 | n.a. | BMB* |
| 2 | 20.47 | n.a. | 52.429 | 24.169 | 97.50 | n.a. | BMB* |
| Total: | | | 54.689 | 24.790 | 100.00 | 0.000 | |

**Supplementary Figure 9.** HPLC traces of optical compound **8** from compound **(±)-10.**

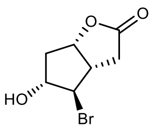


**8**

This sample was further recrystallized from EtOAc. For recrystallized **8**, ee = 99.7%.

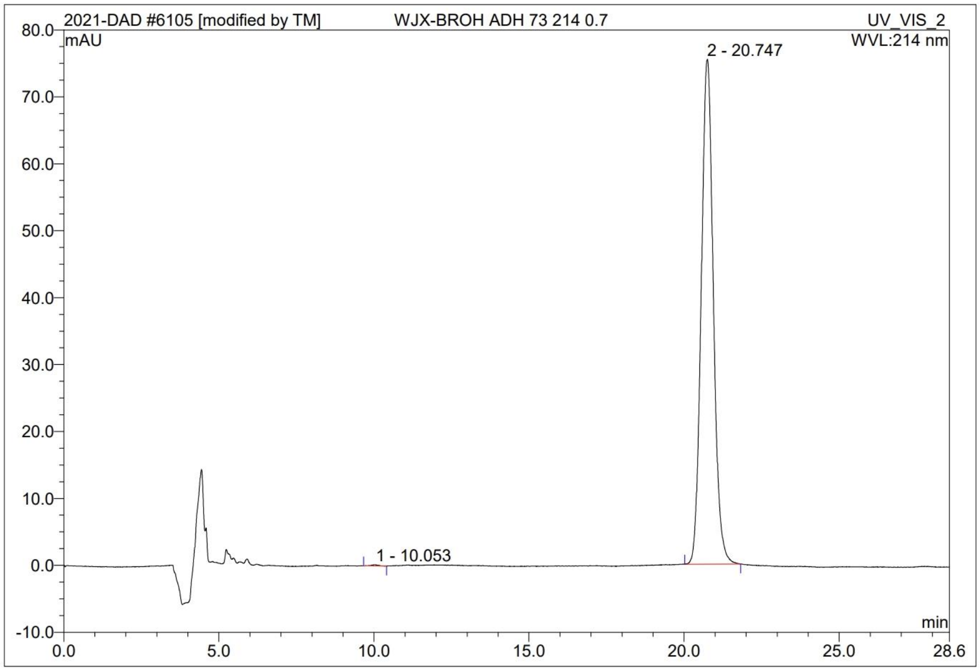


| No. | Ret.Time min | Peak Name | Height mAU | Area mAU*min | Rel.Area % | Amount | Type |
|---|---|---|---|---|---|---|---|
| 1 | 10.05 | n.a. | 0.184 | 0.047 | 0.14 | n.a. | BMB* |
| 2 | 20.75 | n.a. | 75.463 | 34.116 | 99.86 | n.a. | BMB* |
| Total: | | | 75.646 | 34.163 | 100.00 | 0.000 | |

**Supplementary Figure 10.** HPLC traces of recrystallized compound **8.**

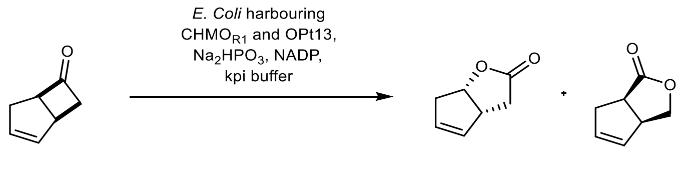


**(±)-10** **9** **14**

The racemic and optical compound **14** were analyzed by GC to determine the retention times and enantiomeric excess. GC analysis was performed on the Agilent 8890 GC System equipped with FID and CP-Chirasil-Dex CB column (25 m × 250 μm × 0.25 μm).

The compound **14** prepared from compound **(±)-10**, 97.0% ee.

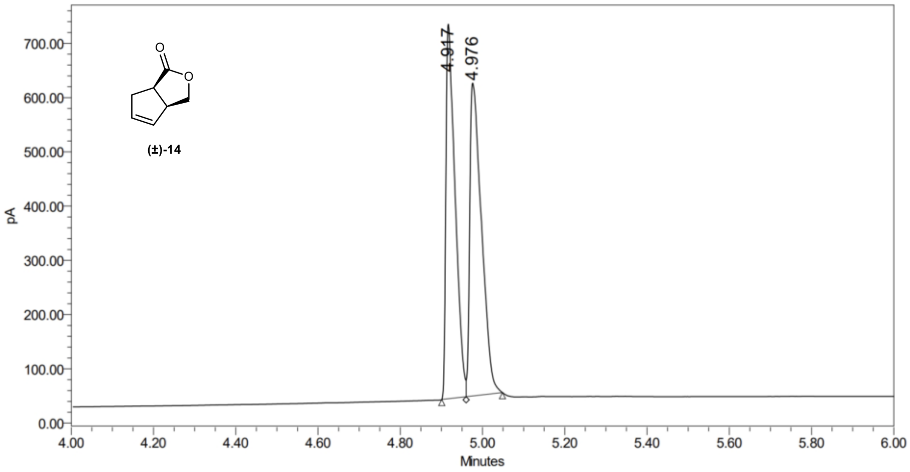


| | RT | Area | % Area | Height |
|---|---|---|---|---|
| 1 | 4.917 | 1076 | 48.52 | 690 |
| 2 | 4.976 | 1141 | 51.48 | 577 |

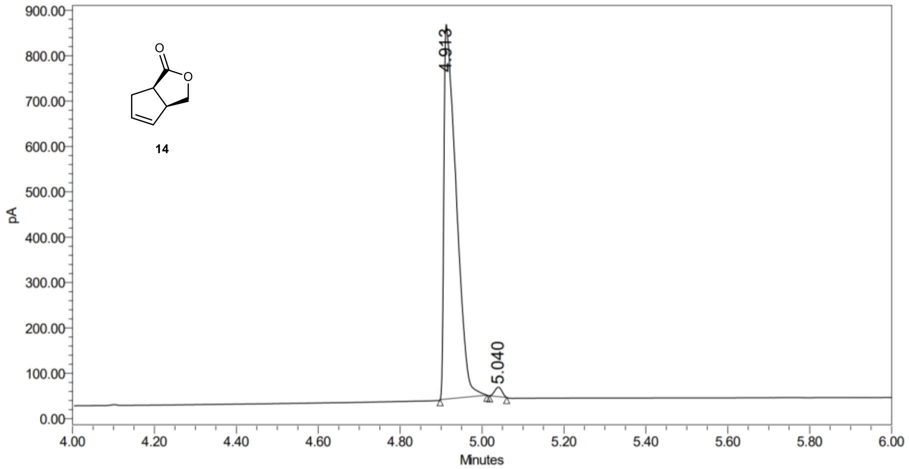


| | RT | Area | % Area | Height |
|---|---|---|---|---|
| 1 | 4.913 | 1712 | 98.52 | 825 |
| 2 | 5.040 | 26 | 1.48 | 21 |

**Supplementary Figure 11.** GC traces of racemic and optical compound **14.**

## IV NMR Spectra of Compounds

**Supplementary Figure 12. $^1H$ NMR Spectrum of compound 11 (500 MHz, $CDCl_3$)**

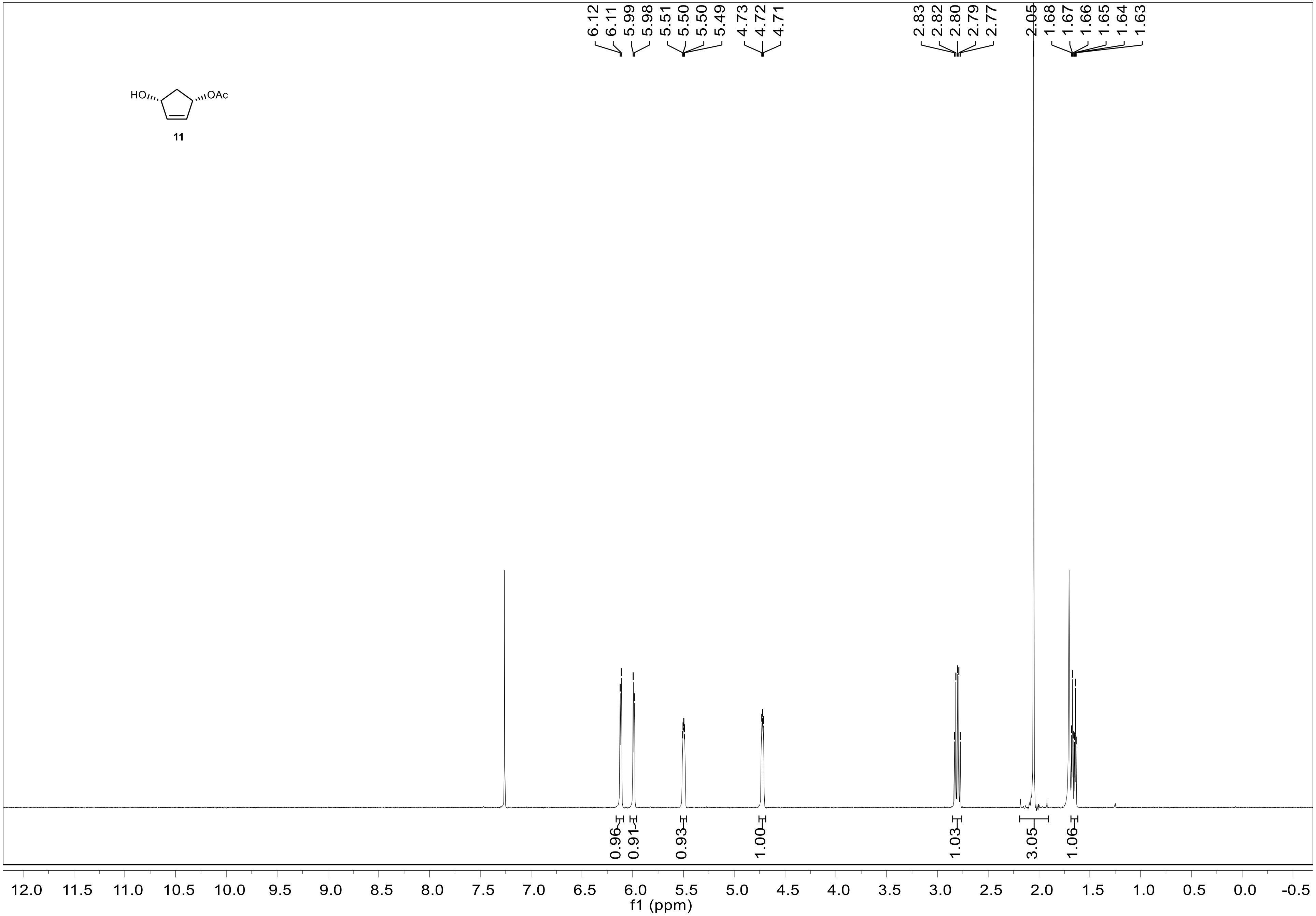

**Supplementary Figure 13. $^{13}C$ NMR Spectrum of compound 11 (126 MHz, $CDCl_3$)**

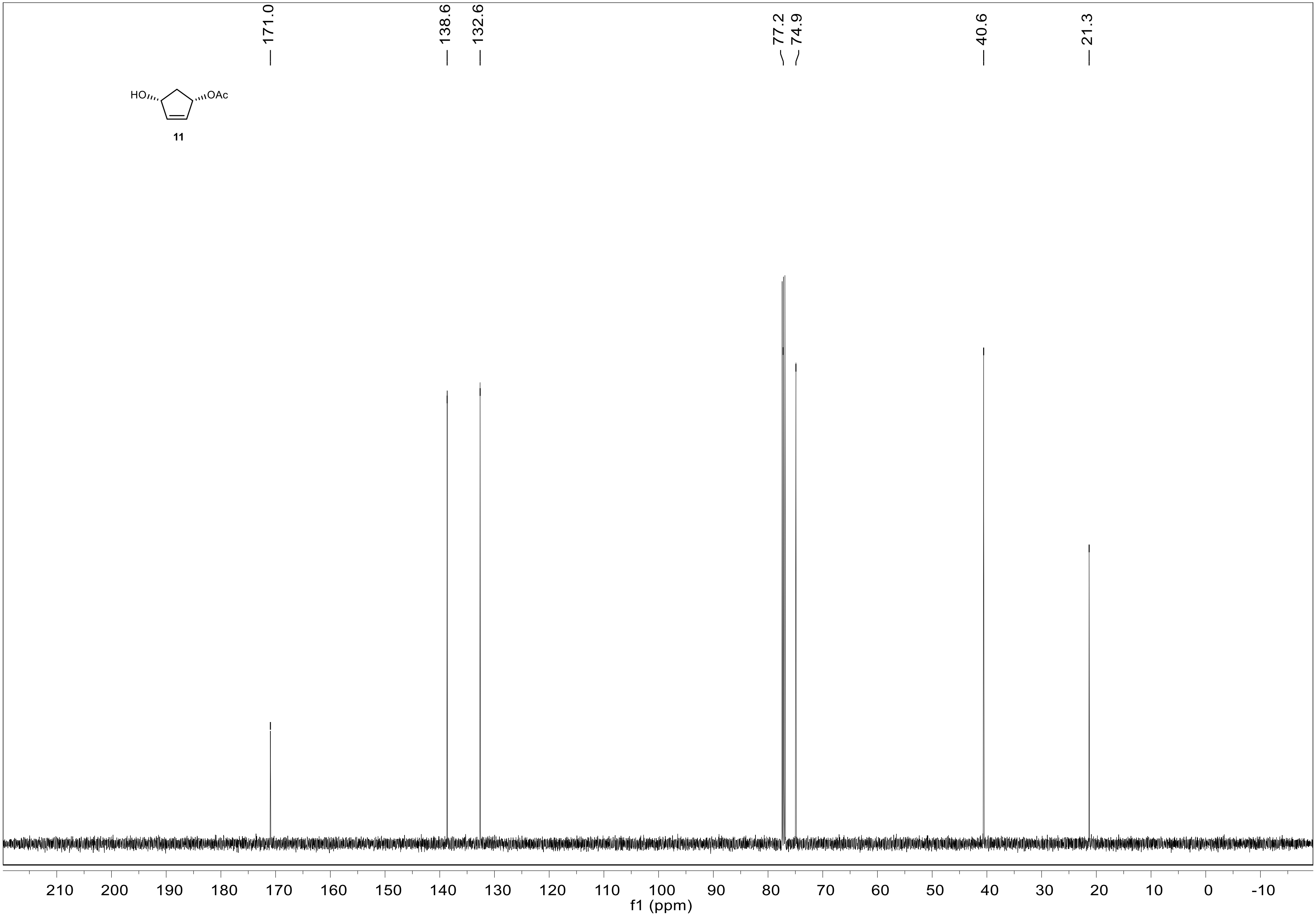

**Supplementary Figure 14. $^{1}$H NMR Spectrum of compound 9 (500 MHz, $CDCl_3$)**

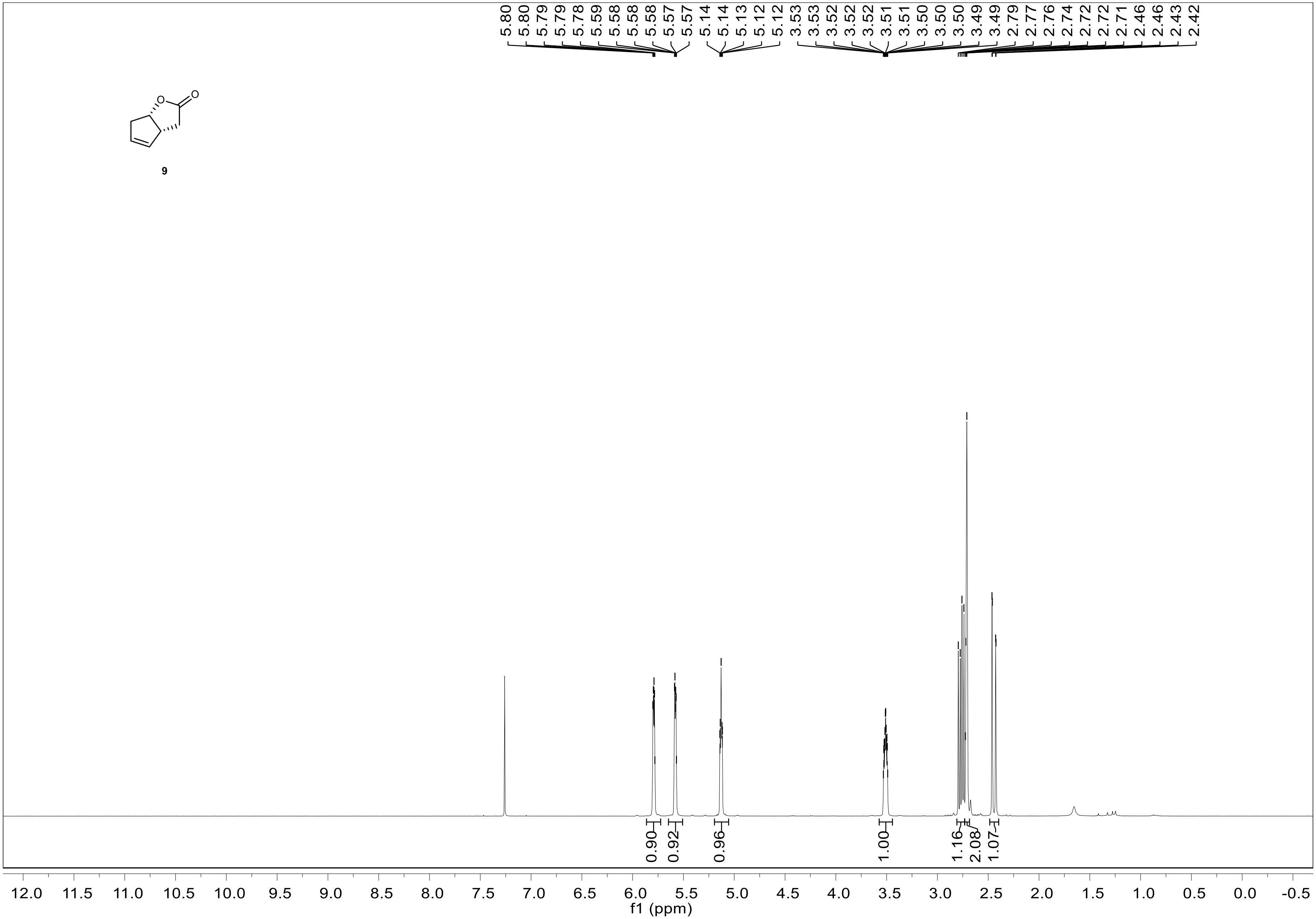

**Supplementary Figure 15. $^{13}C$ NMR Spectrum of compound 9 (126 MHz, $CDCl_3$)**

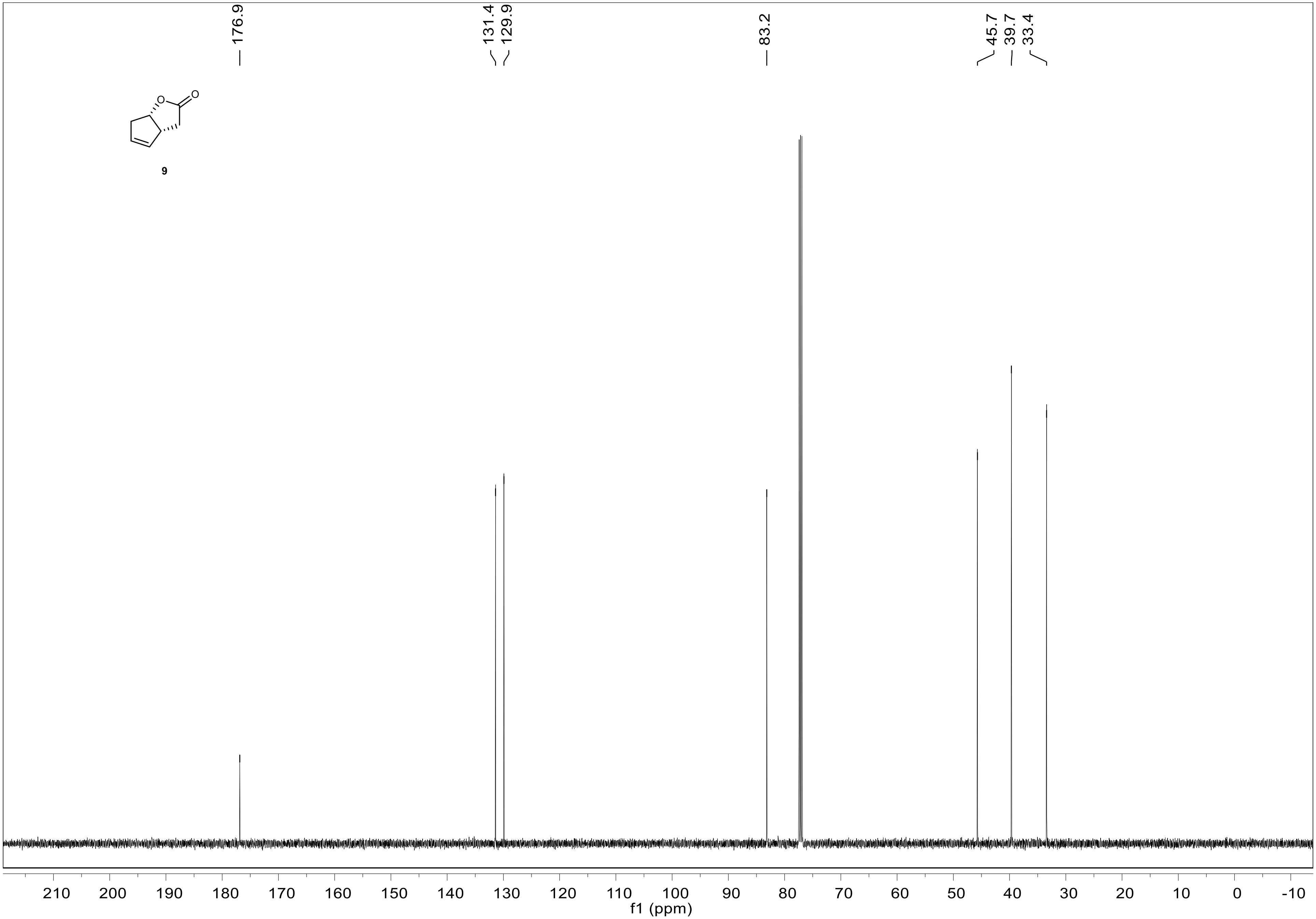

**Supplementary Figure 16. $^{1}$H NMR Spectrum of compound 8 (500 MHz, $CDCl_3$)**

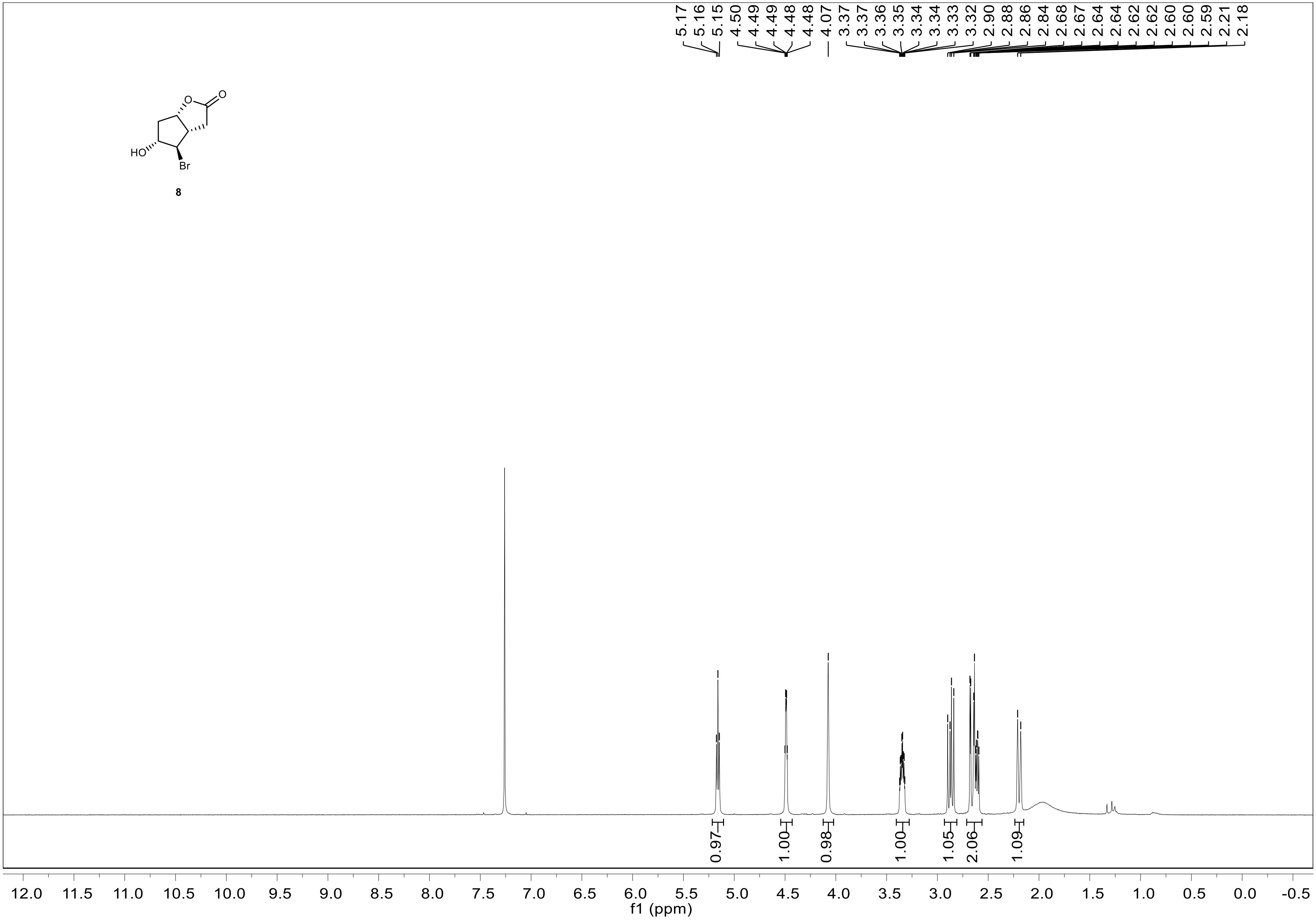

**Supplementary Figure 17. $^{13}$C NMR Spectrum of compound 8 (126 MHz, $CDCl_3$)**

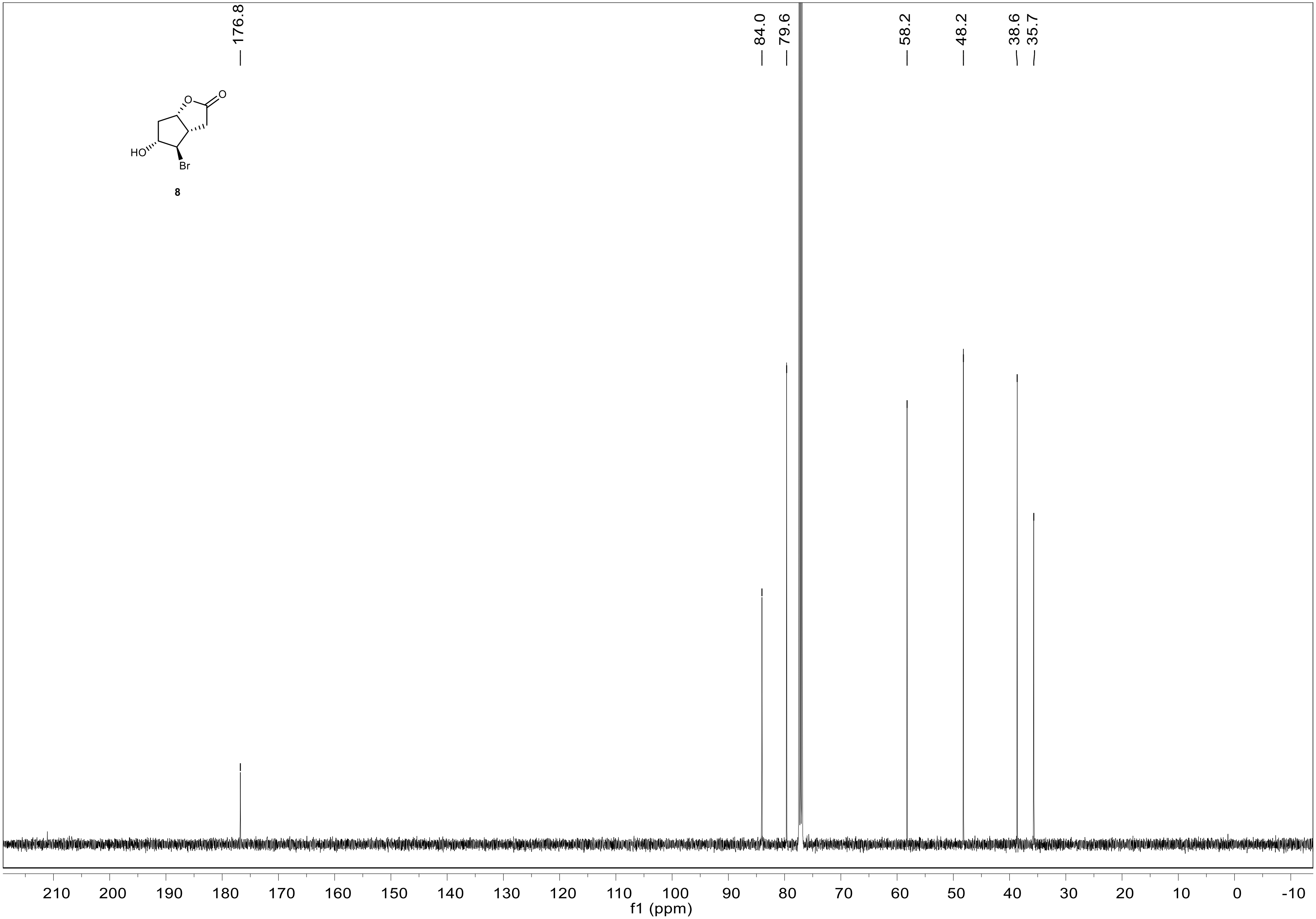

**Supplementary Figure 18. $^1$H NMR Spectrum of compound 16 (500 MHz, $CDCl_3$)**

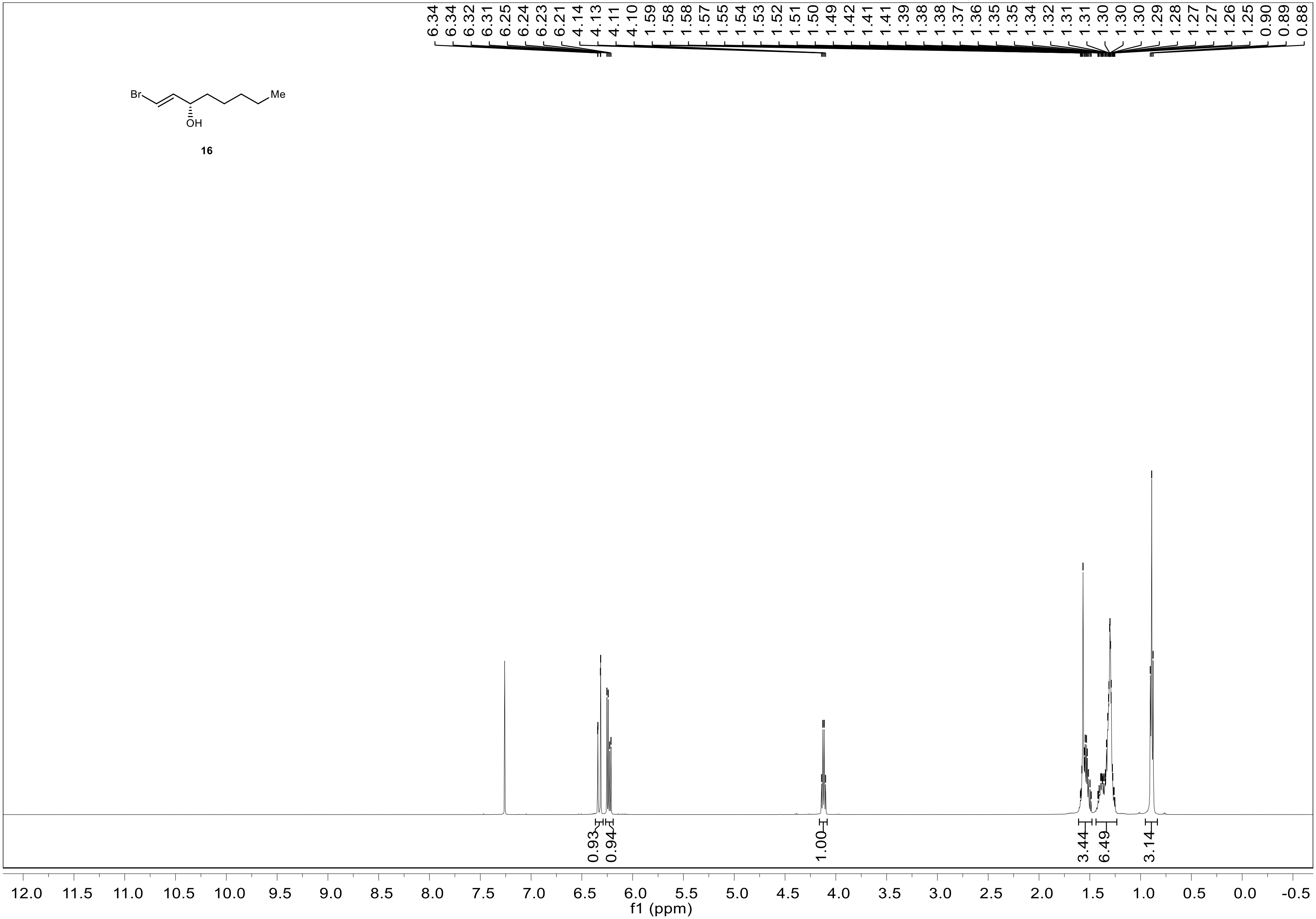

**Supplementary Figure 19. $^{13}C$ NMR Spectrum of compound 16 (126 MHz, $CDCl_3$)**

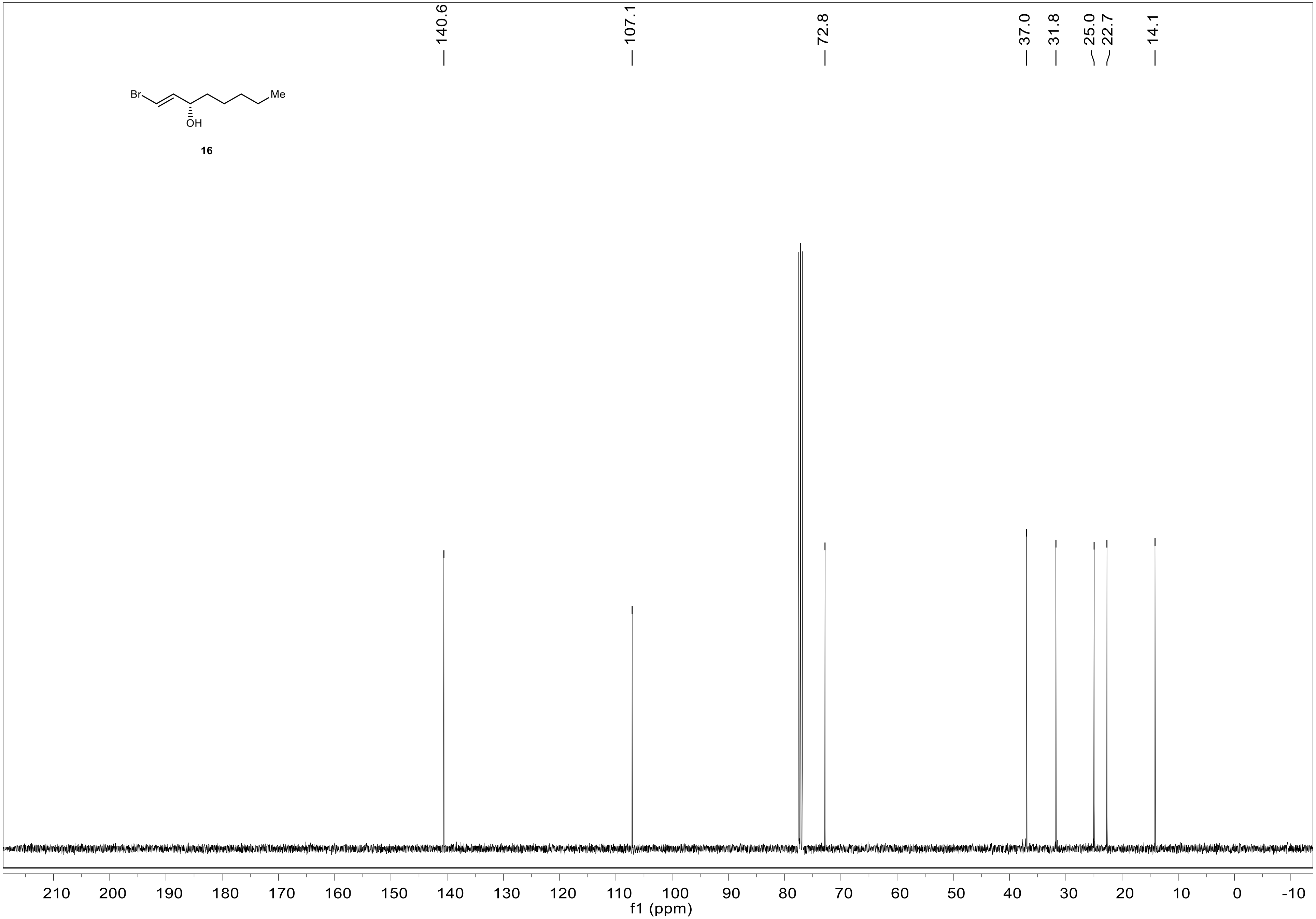

**Supplementary Figure 20. $^1$H NMR Spectrum of compound 16a (500 MHz, $CDCl_3$)**

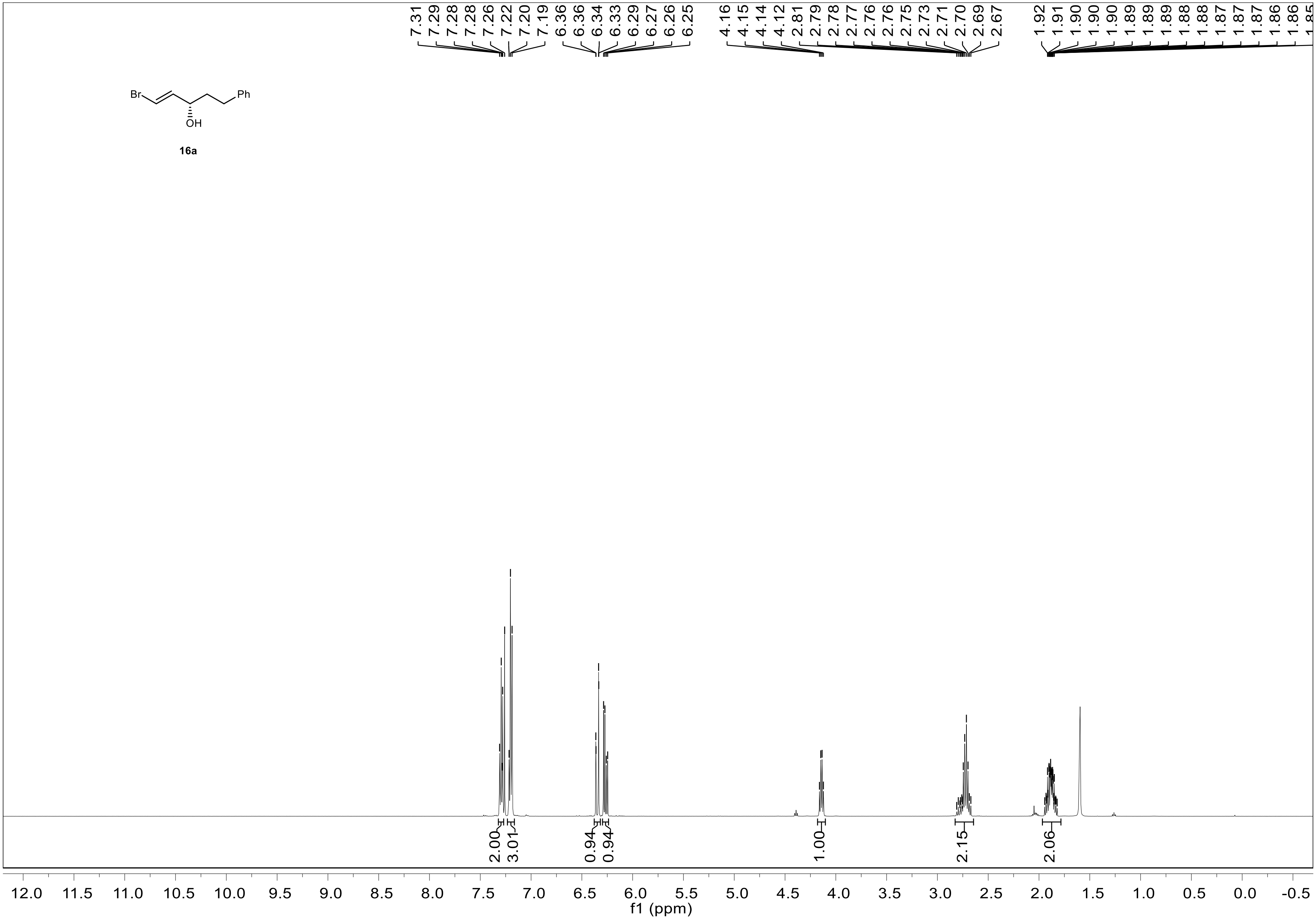

**Supplementary Figure 21. $^{13}$C NMR Spectrum of compound 16a (126 MHz, $CDCl_3$)**

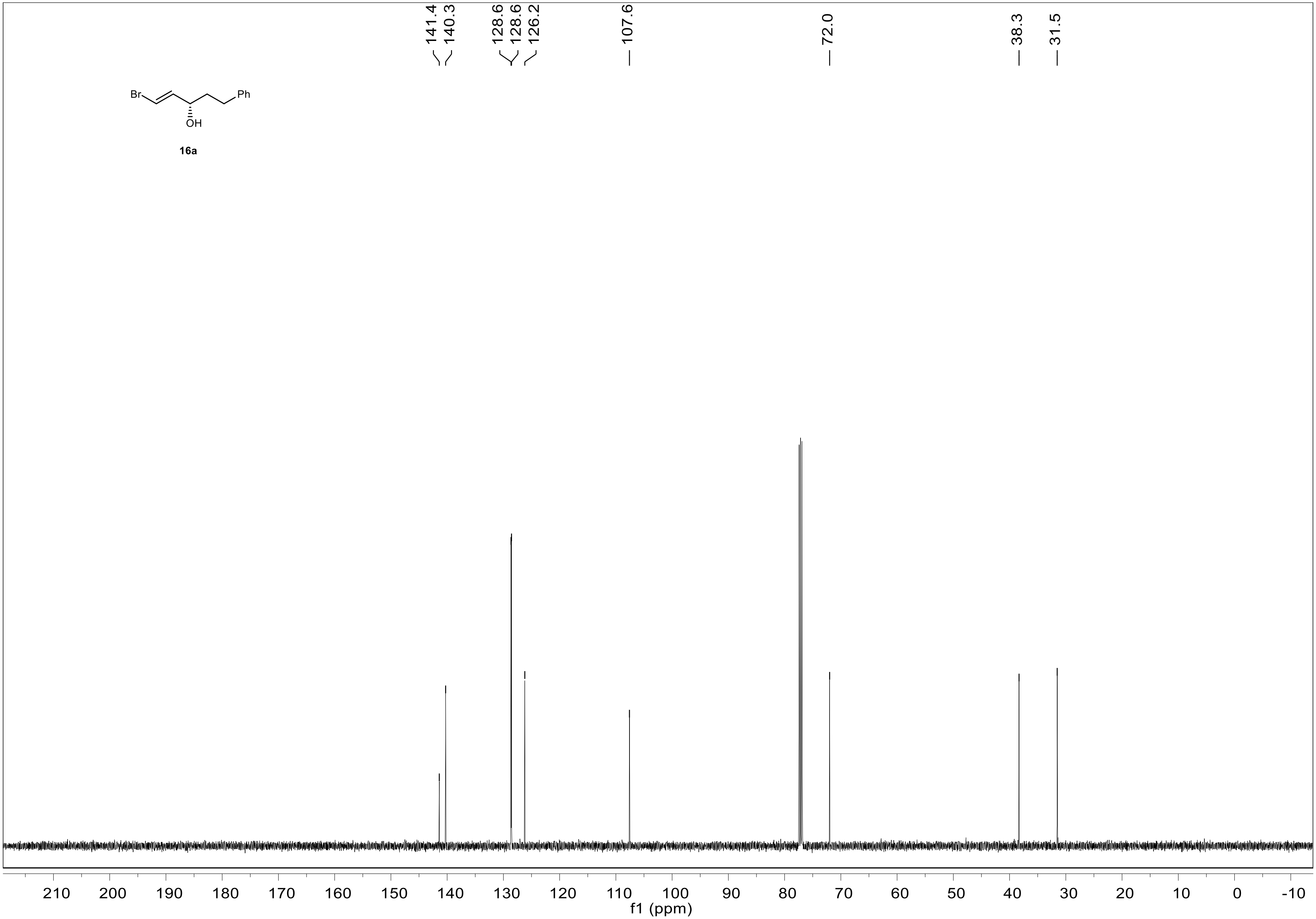

**Supplementary Figure 22. $^{1}$H NMR Spectrum of compound 16b (500 MHz, $CDCl_3$)**

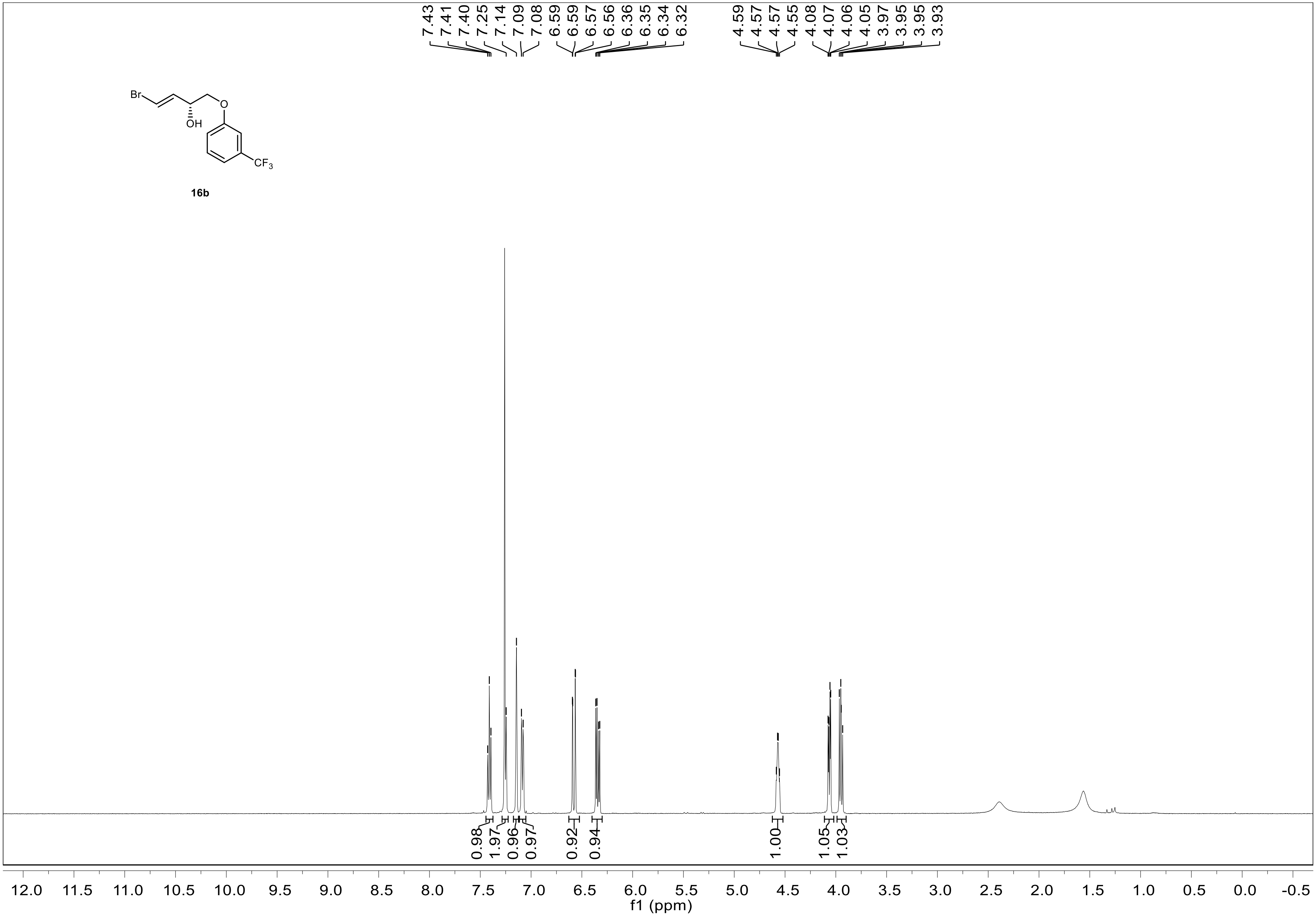

**Supplementary Figure 23. $^{13}$C NMR Spectrum of compound 16b (126 MHz, $CDCl_3$)**

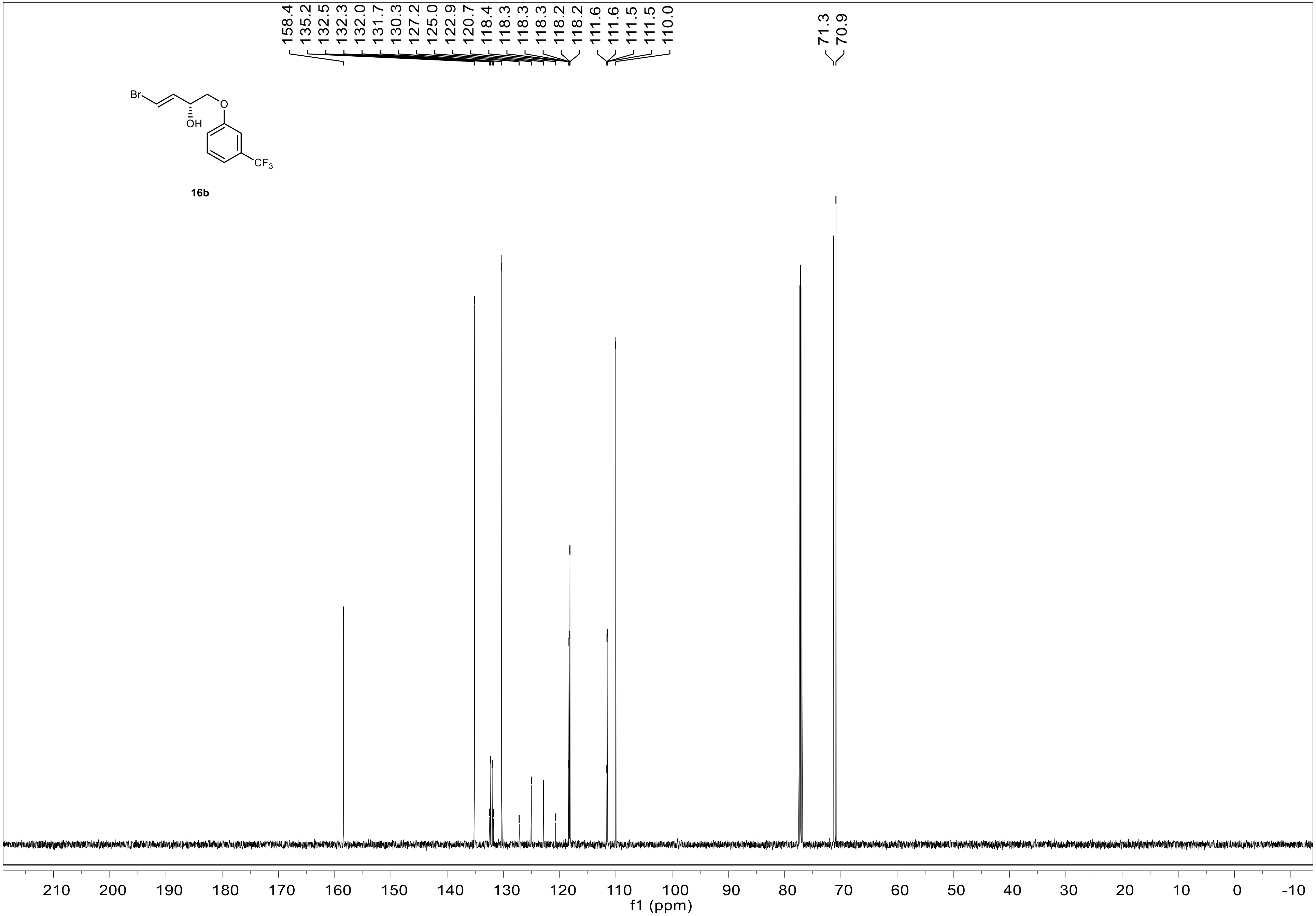

**Supplementary Figure 24. $^{19}$F NMR Spectrum of compound 16b (471 MHz, $CDCl_3$)**

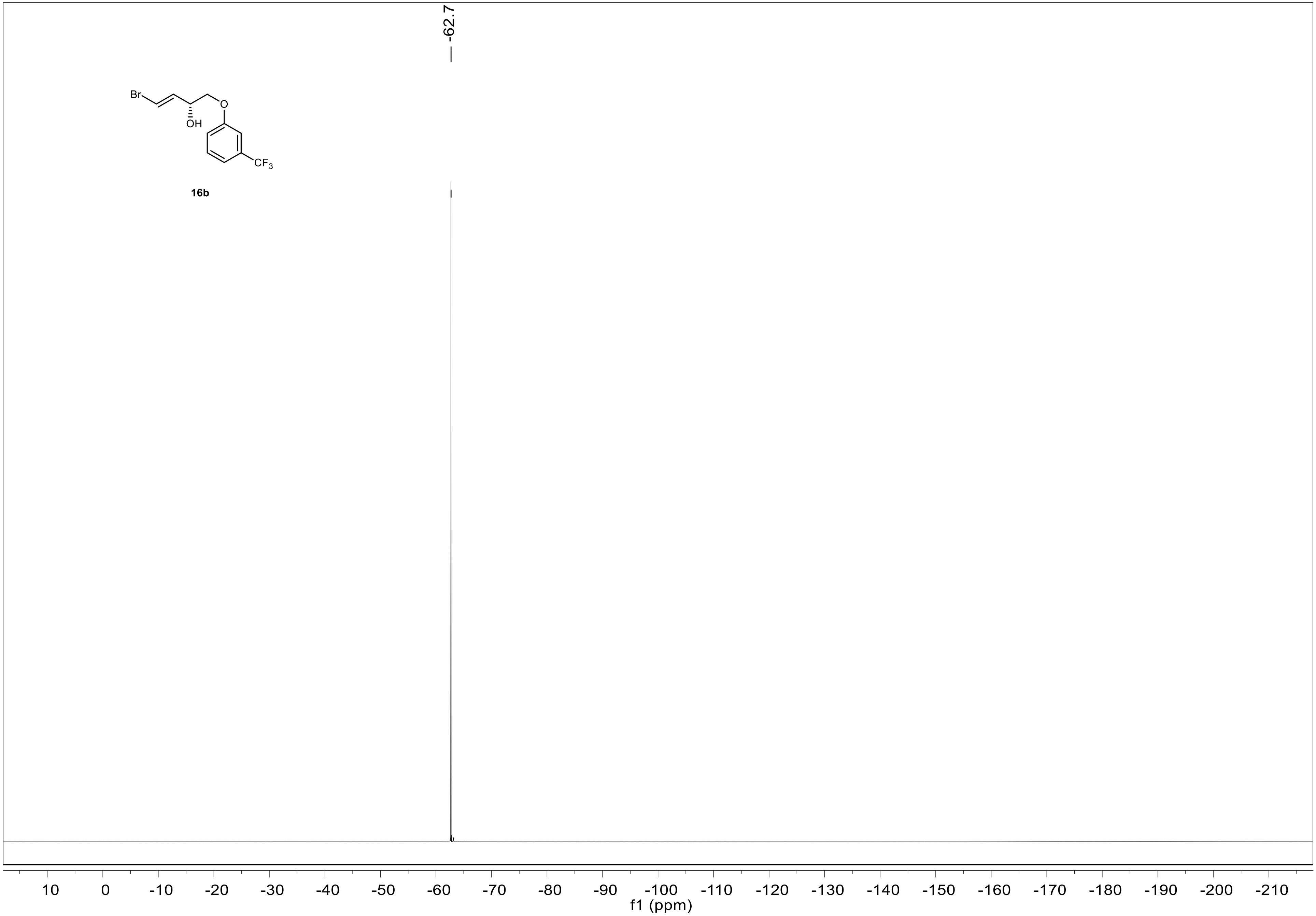

**Supplementary Figure 25. $^{1}$H NMR Spectrum of compound 16c (500 MHz, $CDCl_3$)**

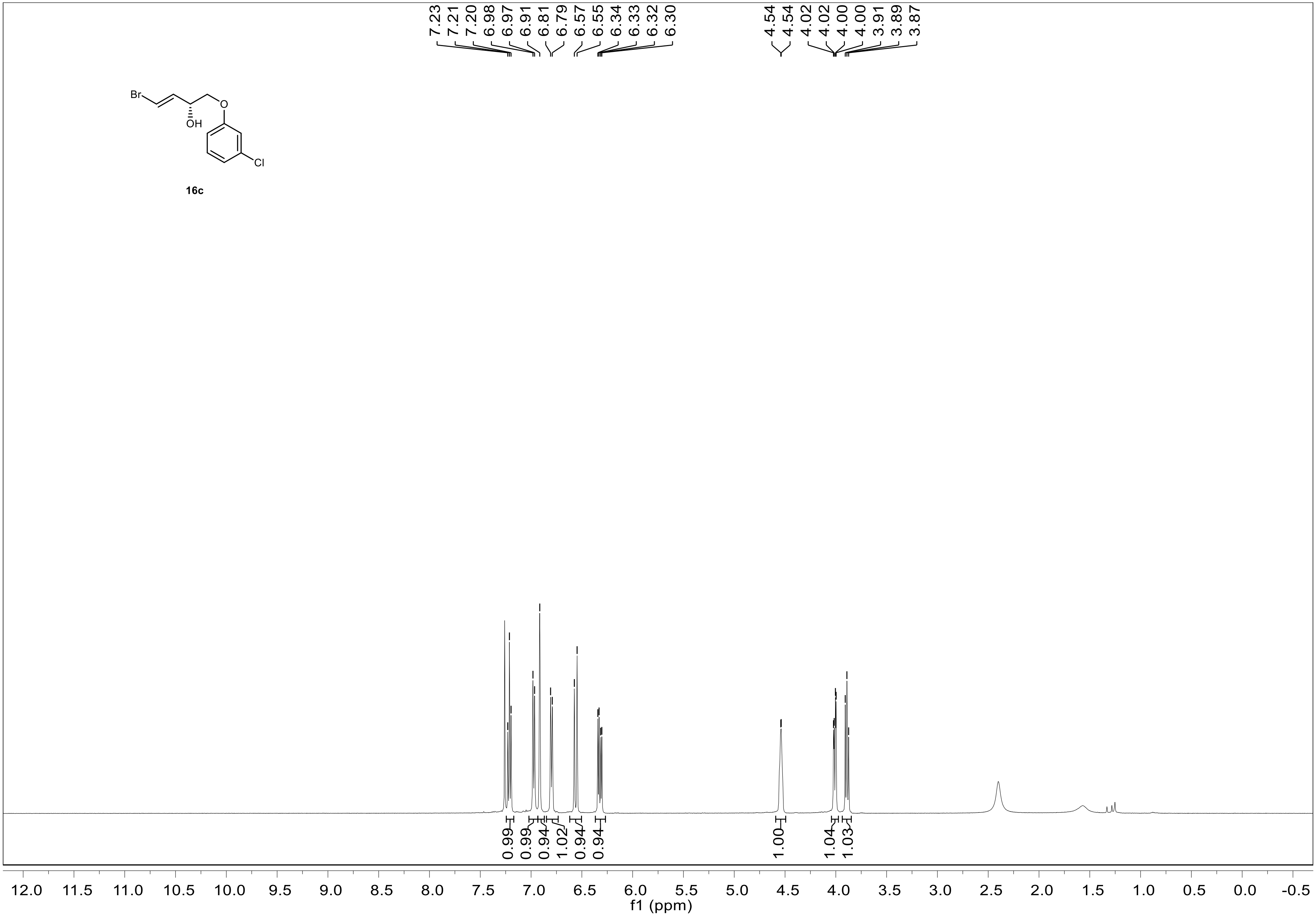

**Supplementary Figure 26. $^{13}$C NMR Spectrum of compound 16c (126 MHz, $CDCl_3$)**

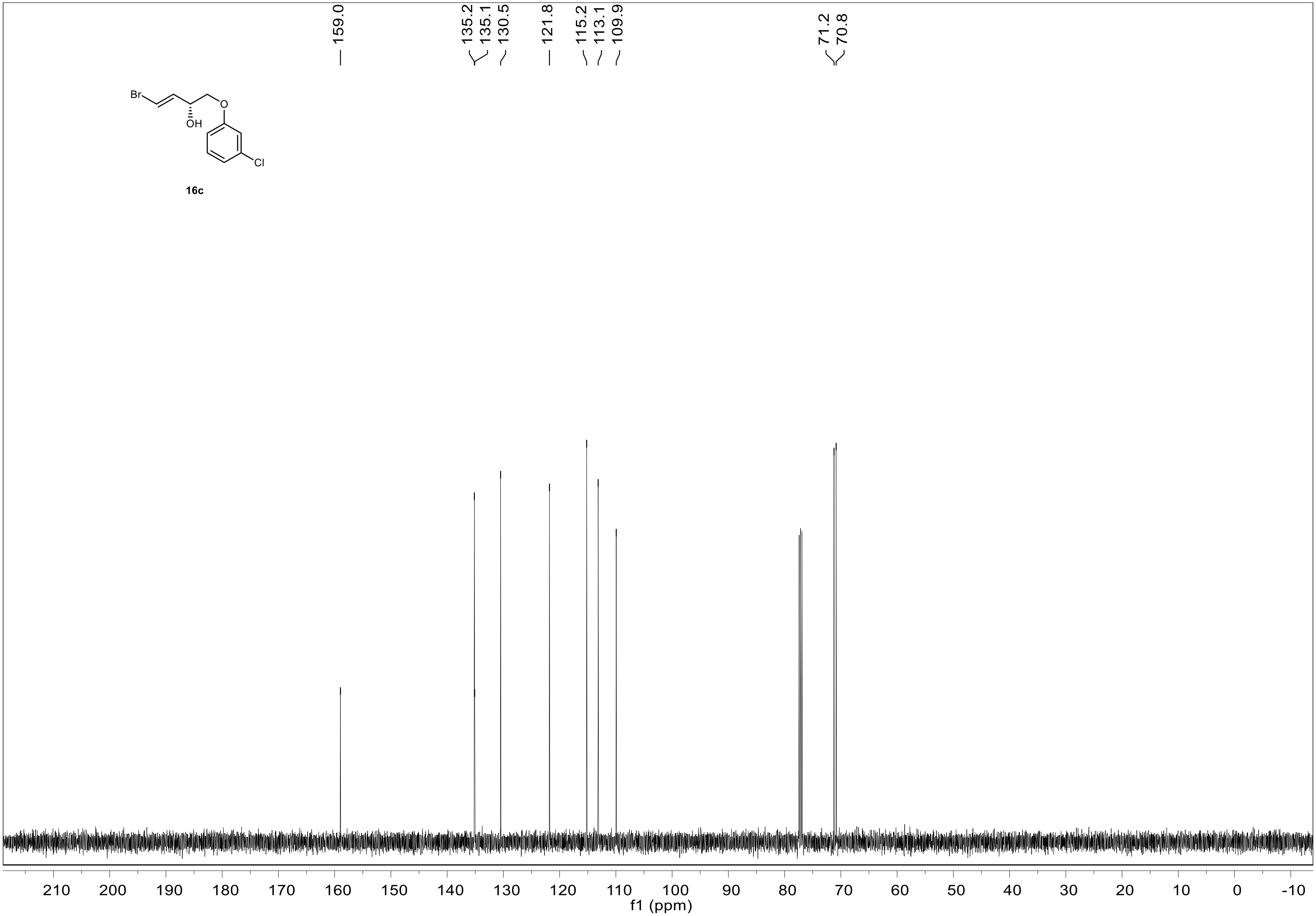

**Supplementary Figure 27. $^{1}$H NMR Spectrum of compound 7 (500 MHz, $CDCl_3$)**

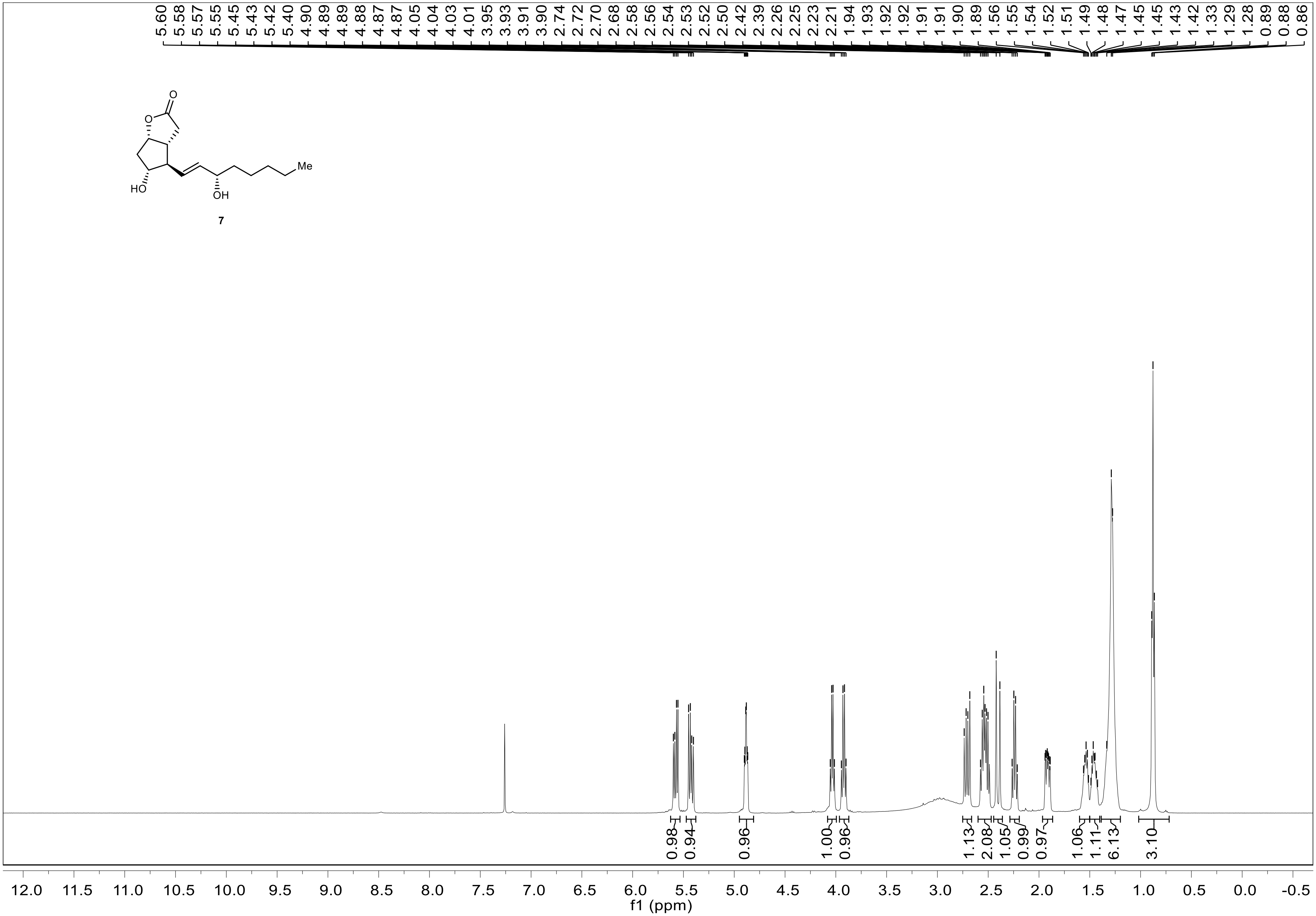

**Supplementary Figure 28.** $^{13}C$ **NMR Spectrum of compound 7 (126 MHz,** $CDCl_3$**)**

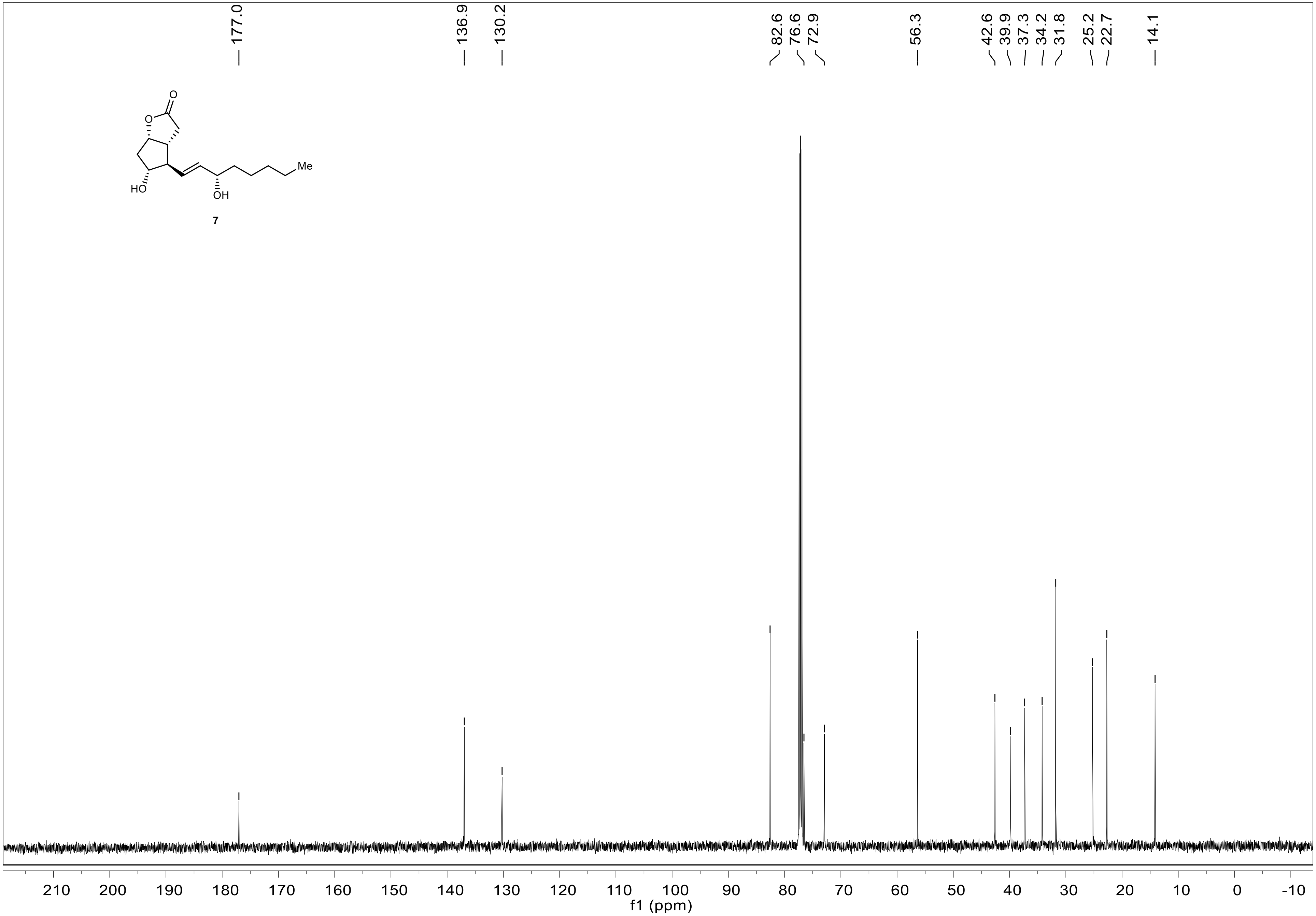

**Supplementary Figure 29. $^{1}H$ NMR Spectrum of $PGF_{2\alpha}$ (1) (700 MHz, $CDCl_3$)**

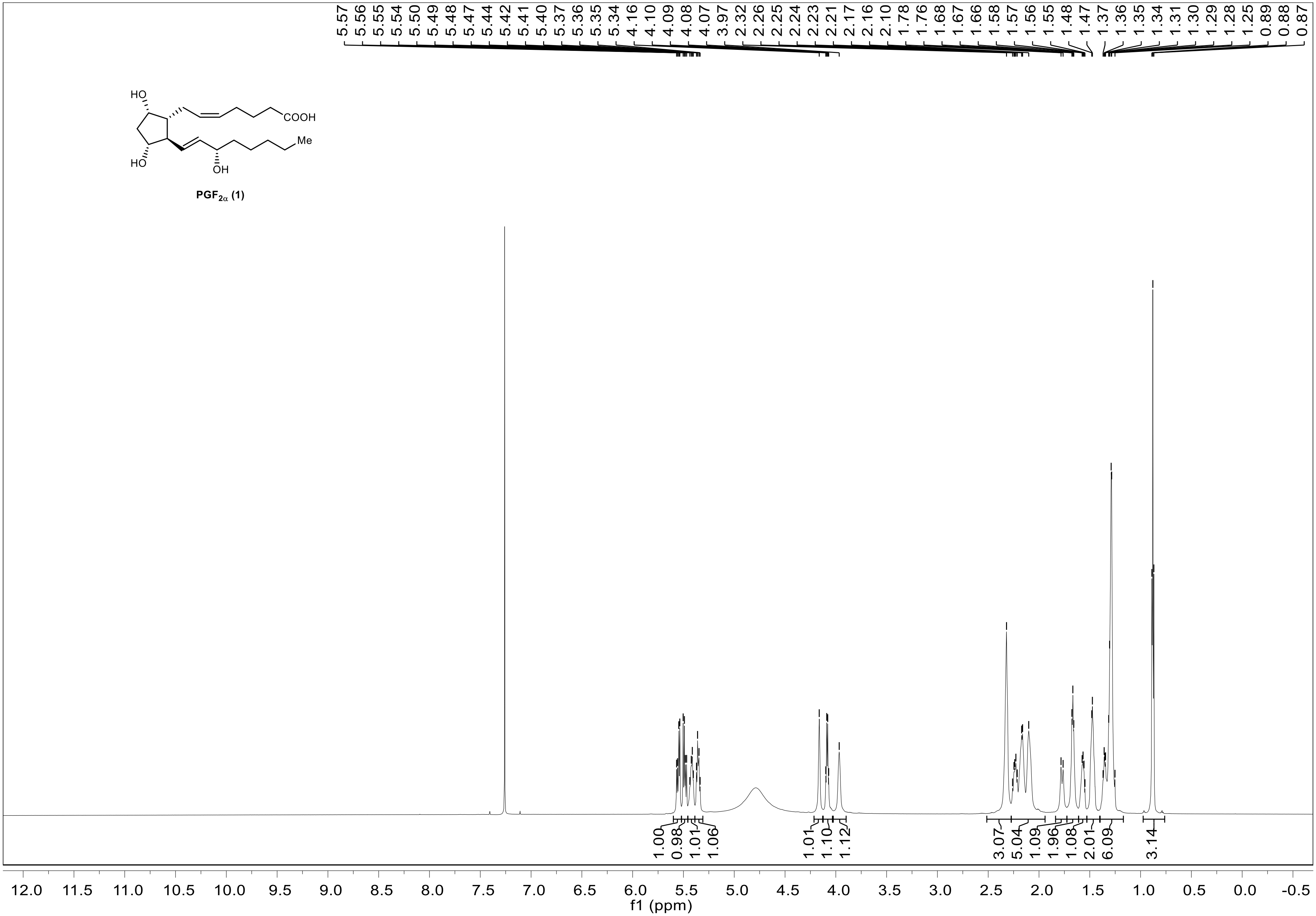

**Supplementary Figure 30.** $^{13}$**C NMR Spectrum of PGF**$_{2\alpha}$ **(1) (176 MHz, CDCl**$_3$**)**

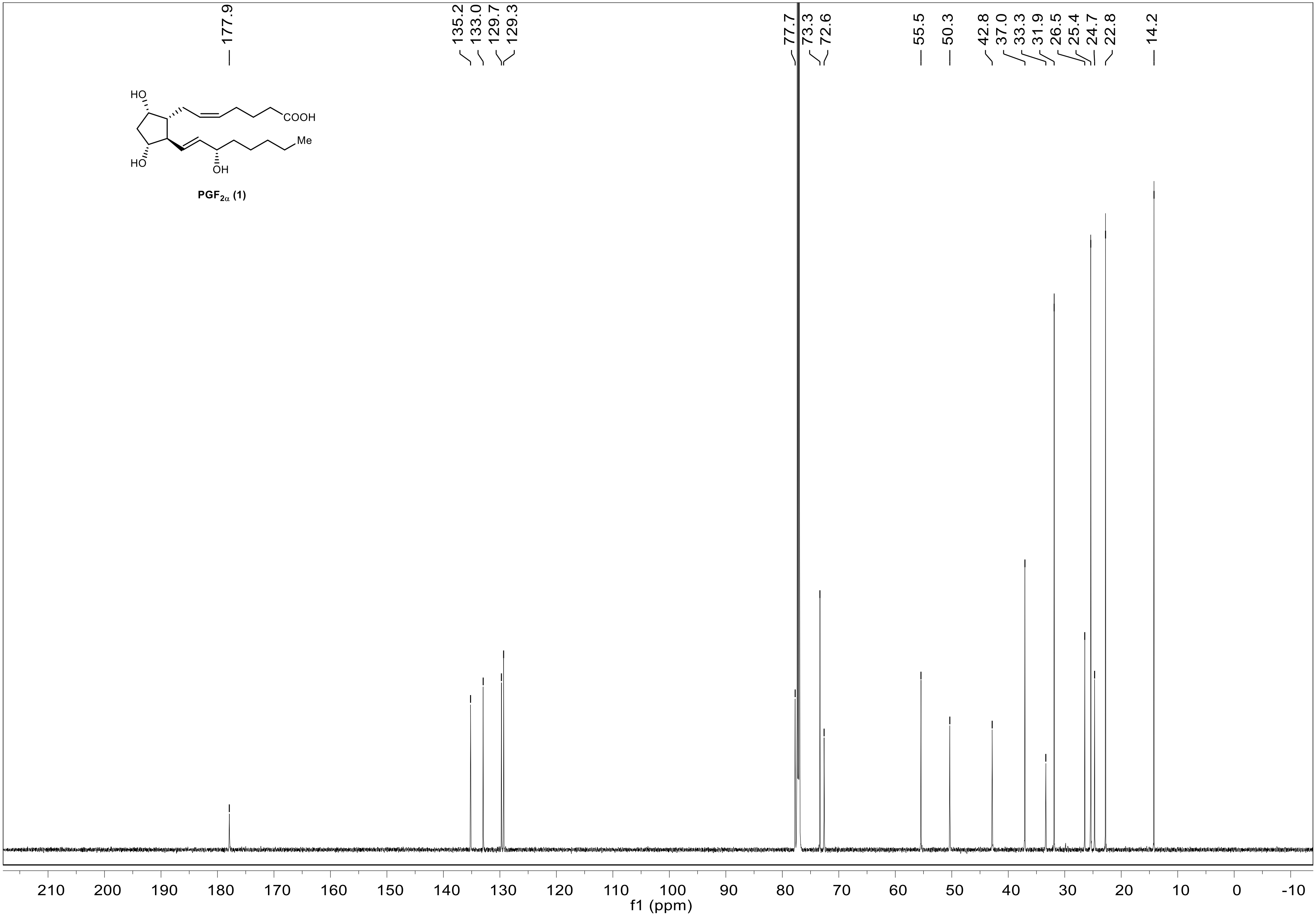

**Supplementary Figure 31. $^1$H NMR Spectrum of compound 20 (500 MHz, $CDCl_3$)**

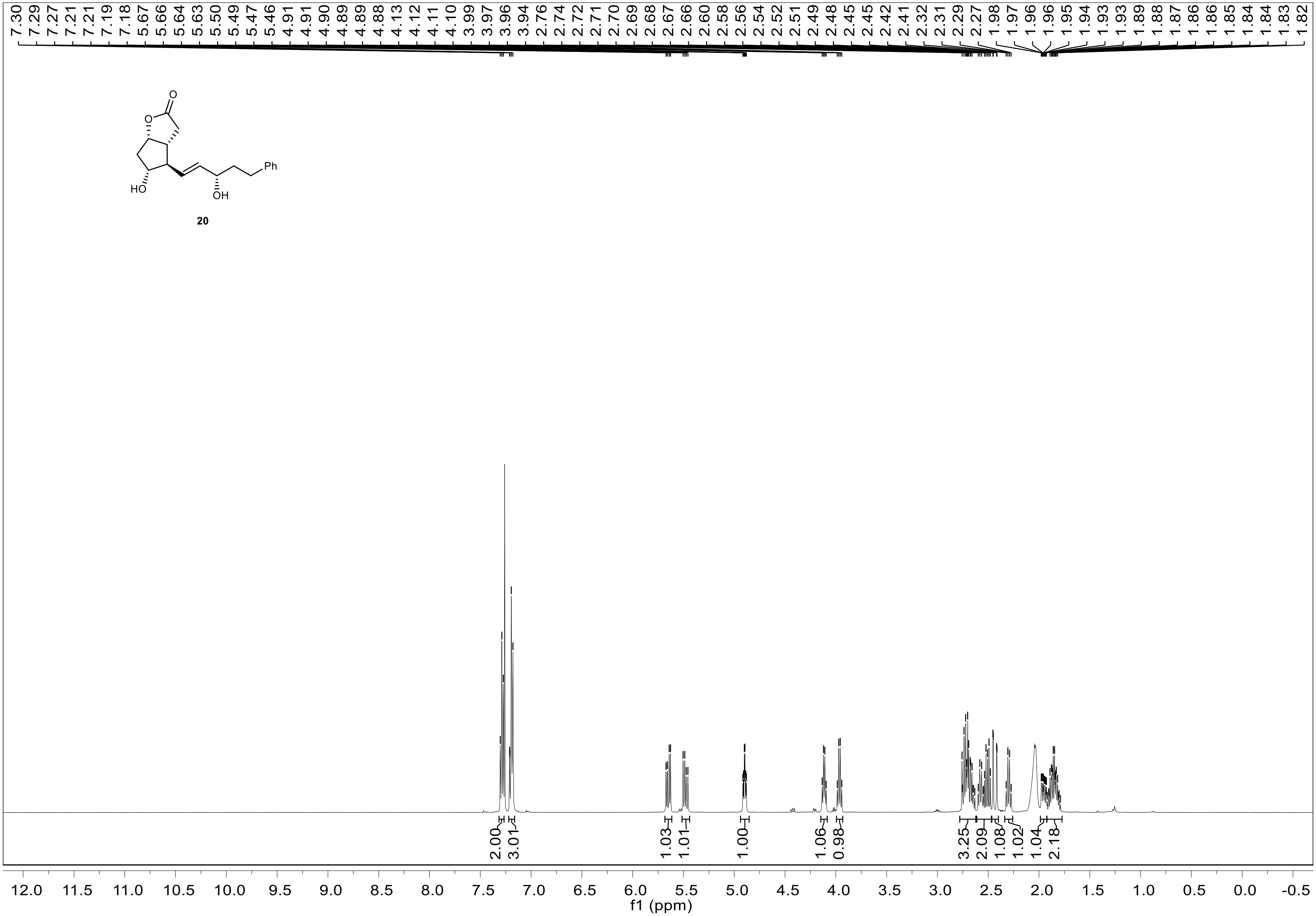

**Supplementary Figure 32. $^{13}$C NMR Spectrum of compound 20 (126 MHz, $CDCl_3$)**

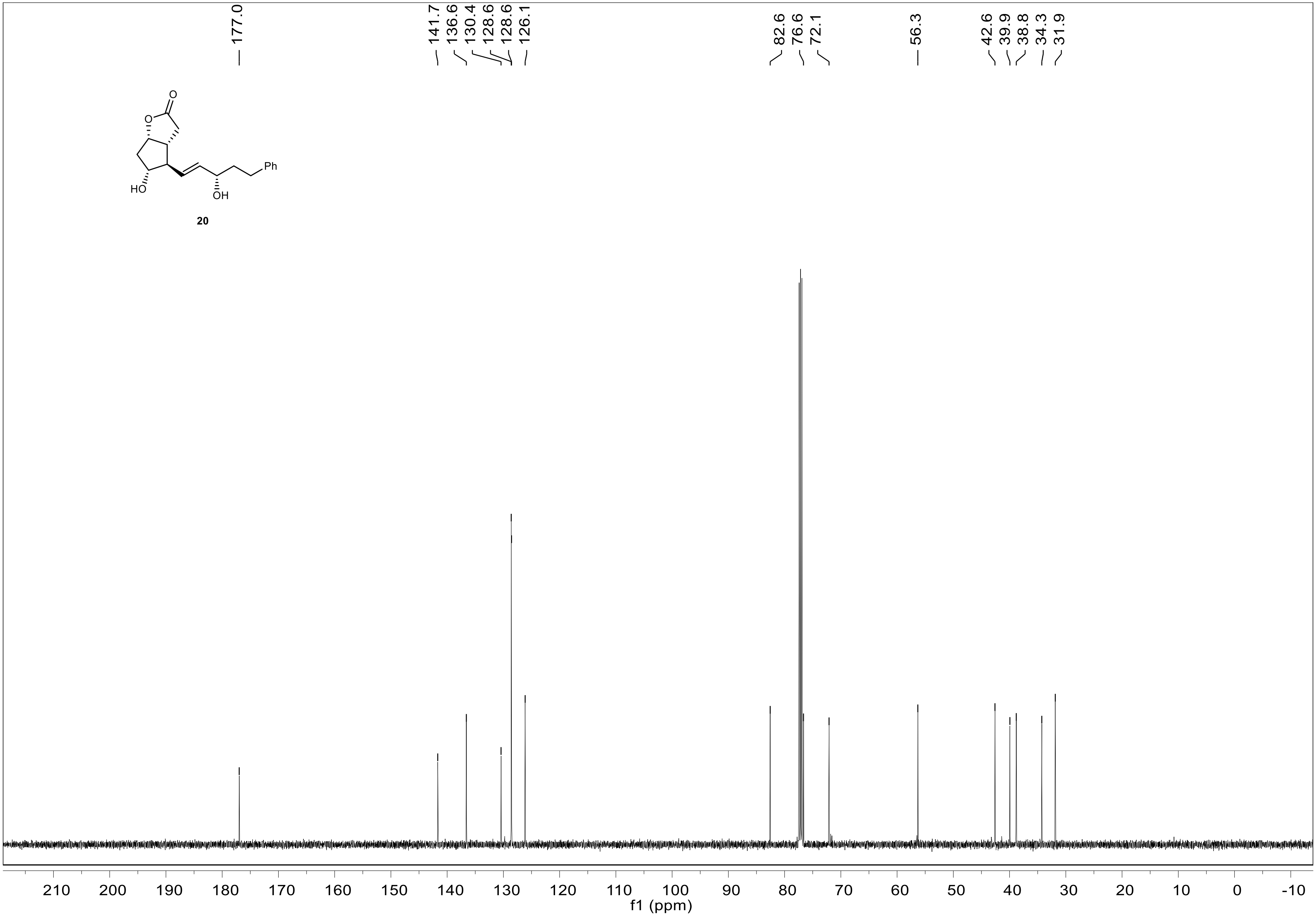

**Supplementary Figure 33. $^{1}H$ NMR Spectrum of Bimatoprost (2) (500 MHz, $CDCl_3$)**

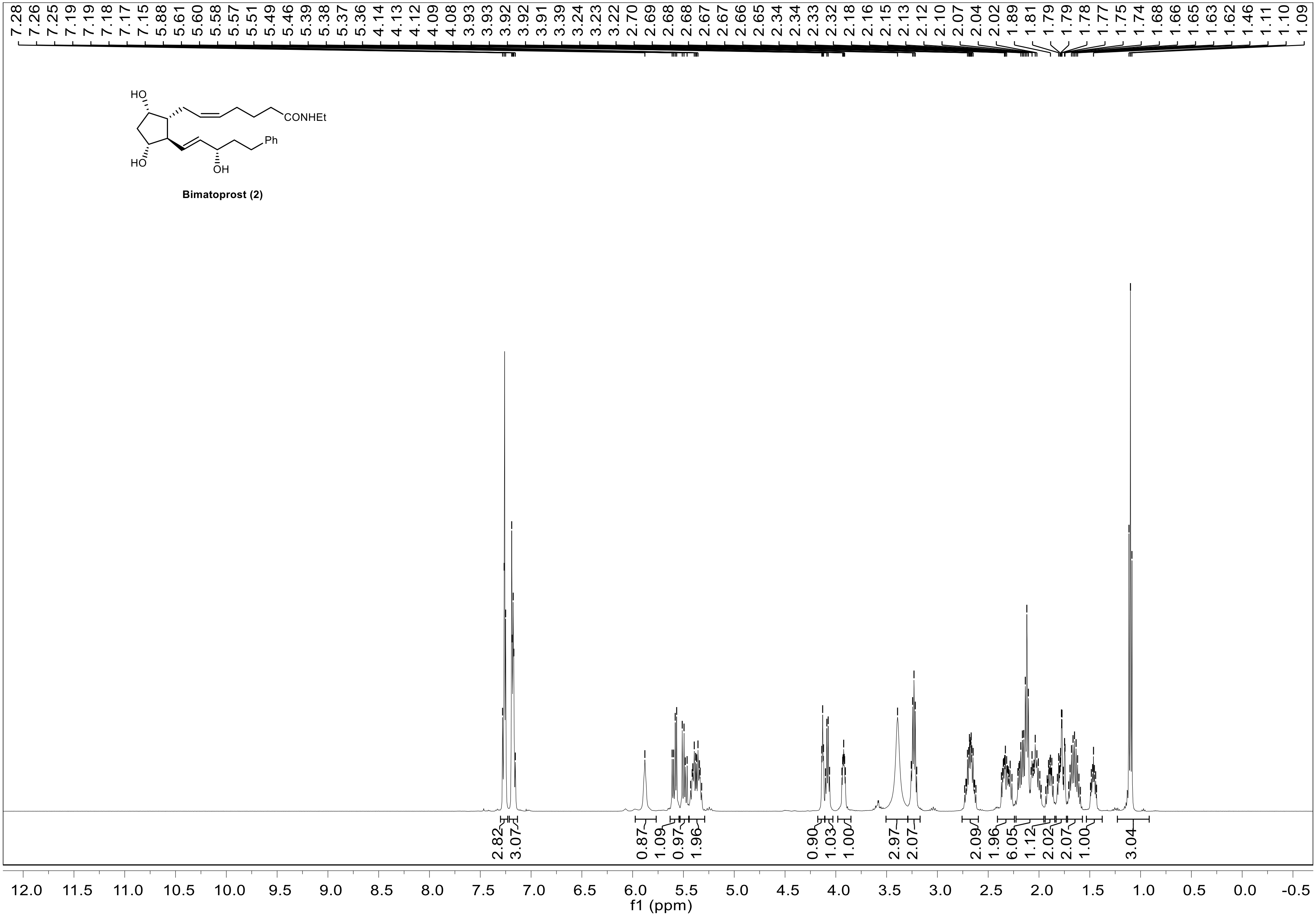

**Supplementary Figure 34. $^{13}C$ NMR Spectrum of Bimatoprost (2) (126 MHz, $CDCl_3$)**

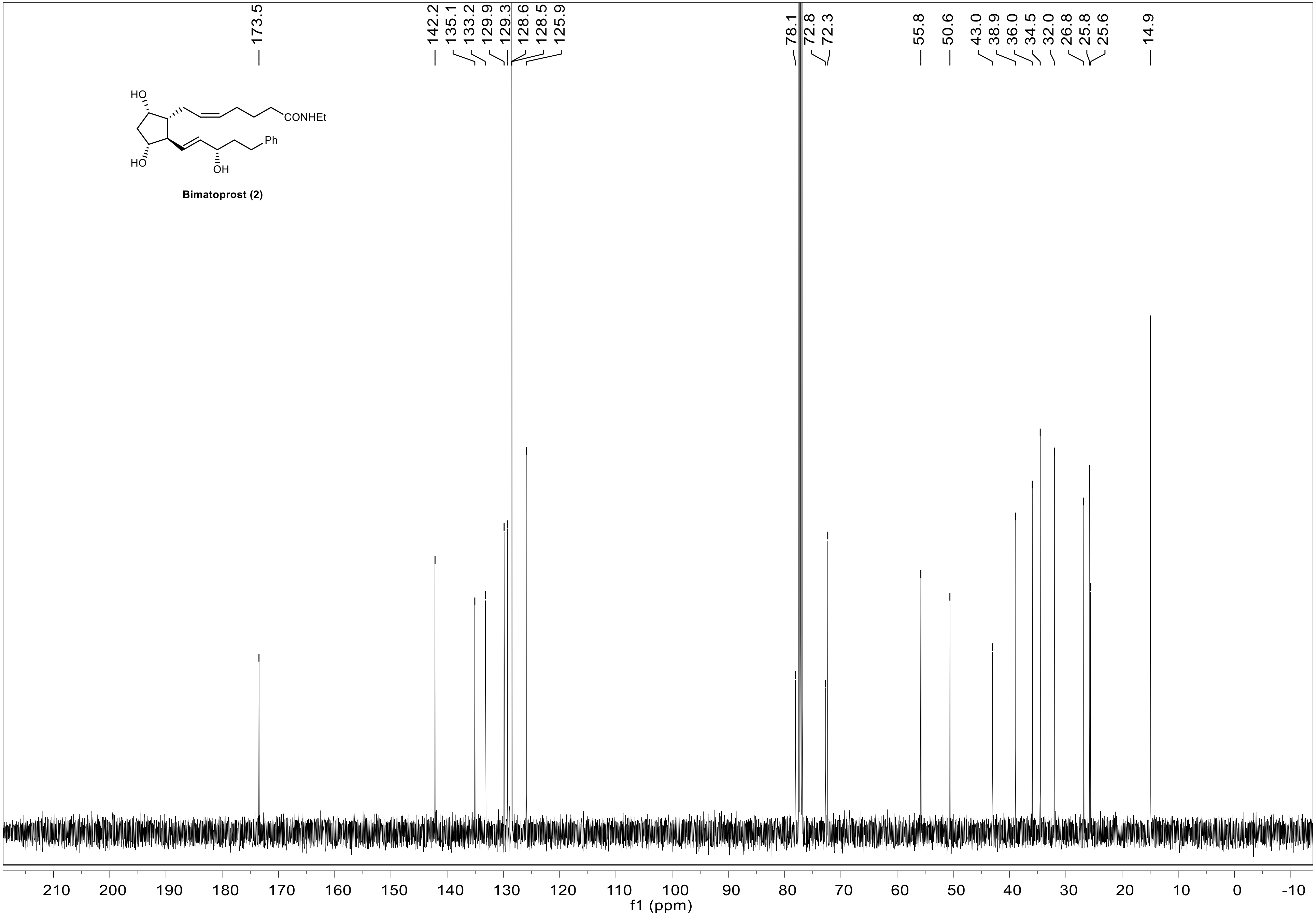

**Supplementary Figure 35. $^{1}$H NMR Spectrum of compound S25 (500 MHz, $CDCl_3$)**

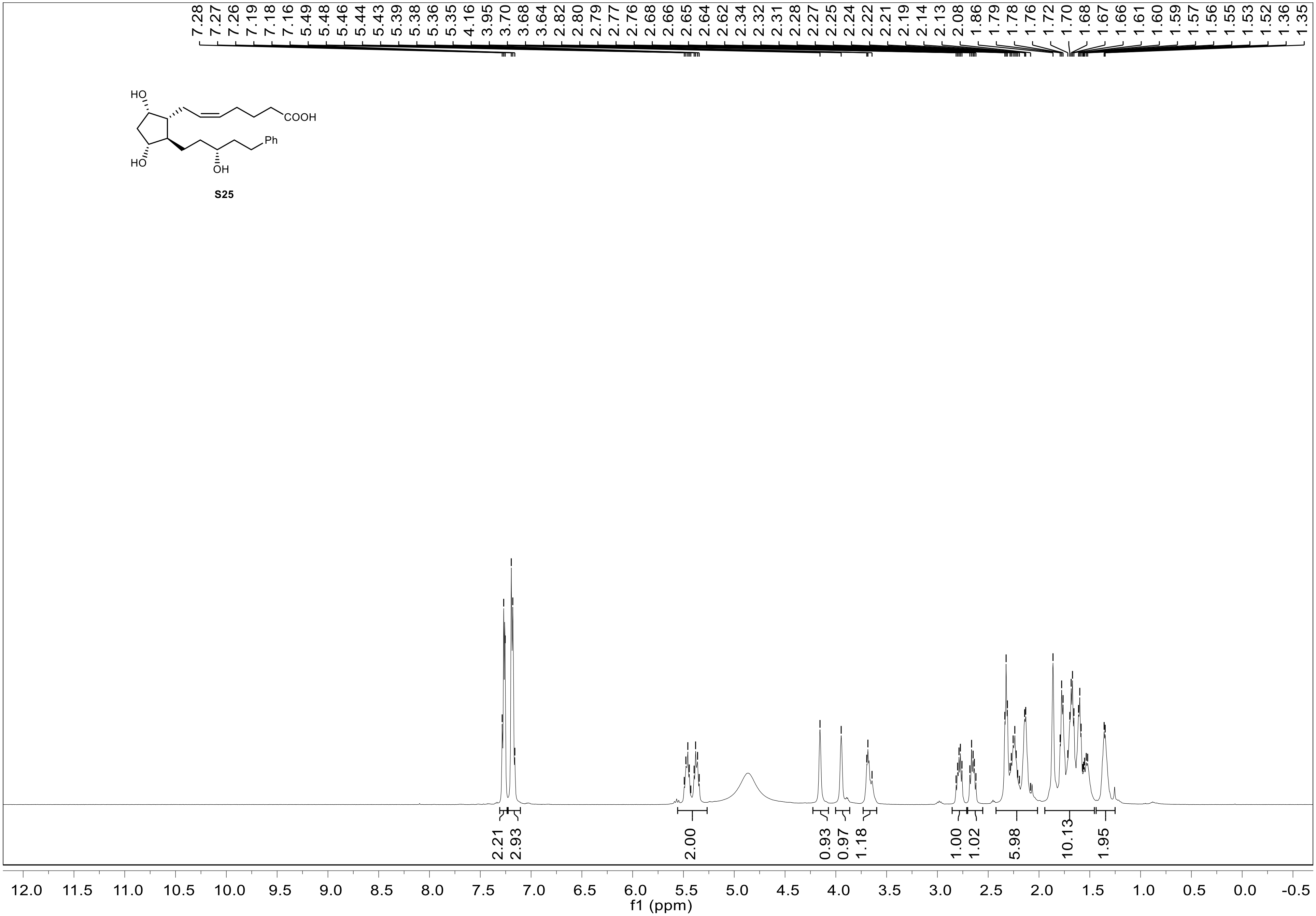

**Supplementary Figure 36. $^{1}$H NMR Spectrum of compound S25 (500 MHz, $CDCl_3$)**

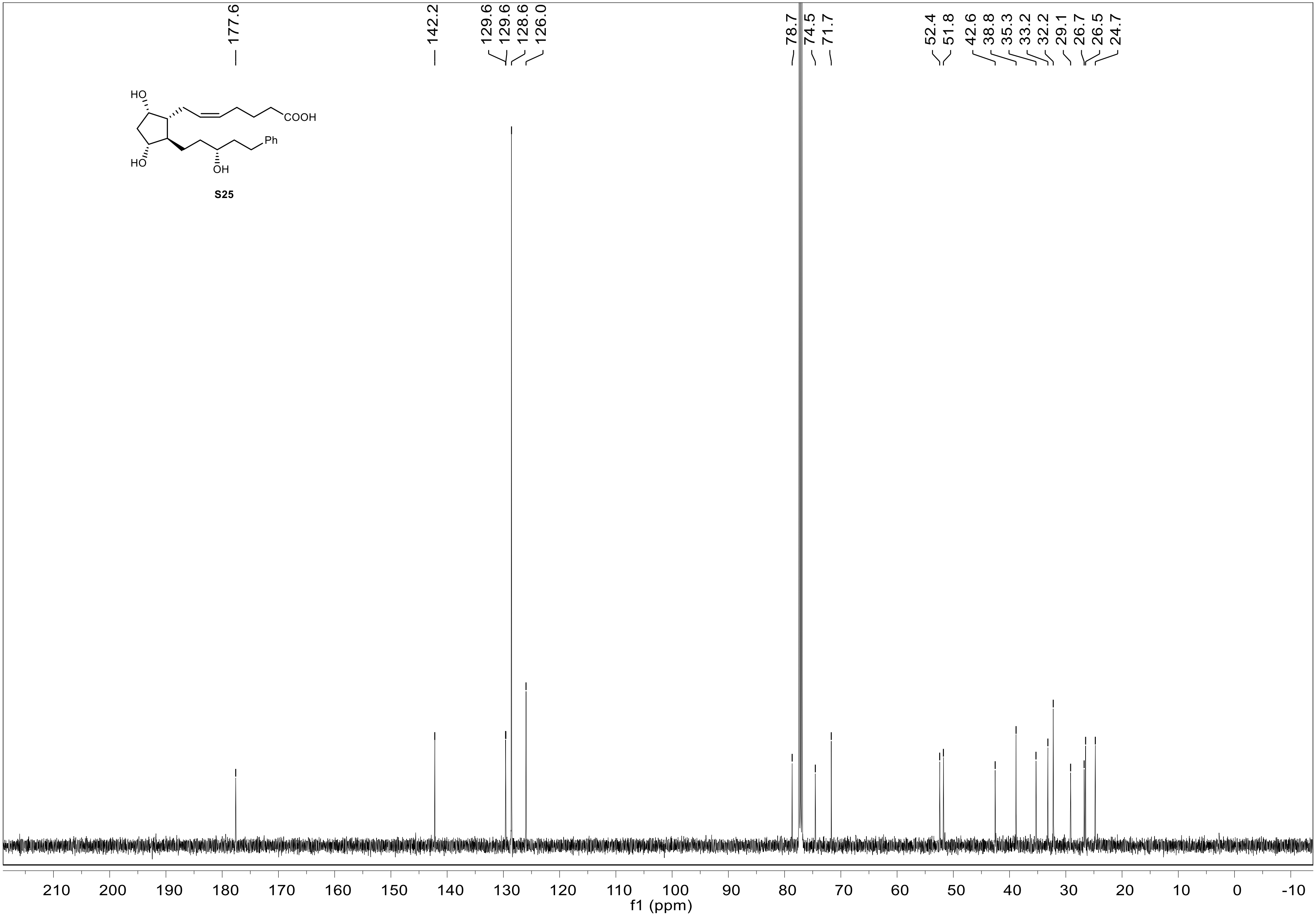

**Supplementary Figure 37. $^{1}$H NMR Spectrum of Latanprost (25) (500 MHz, $CDCl_3$)**

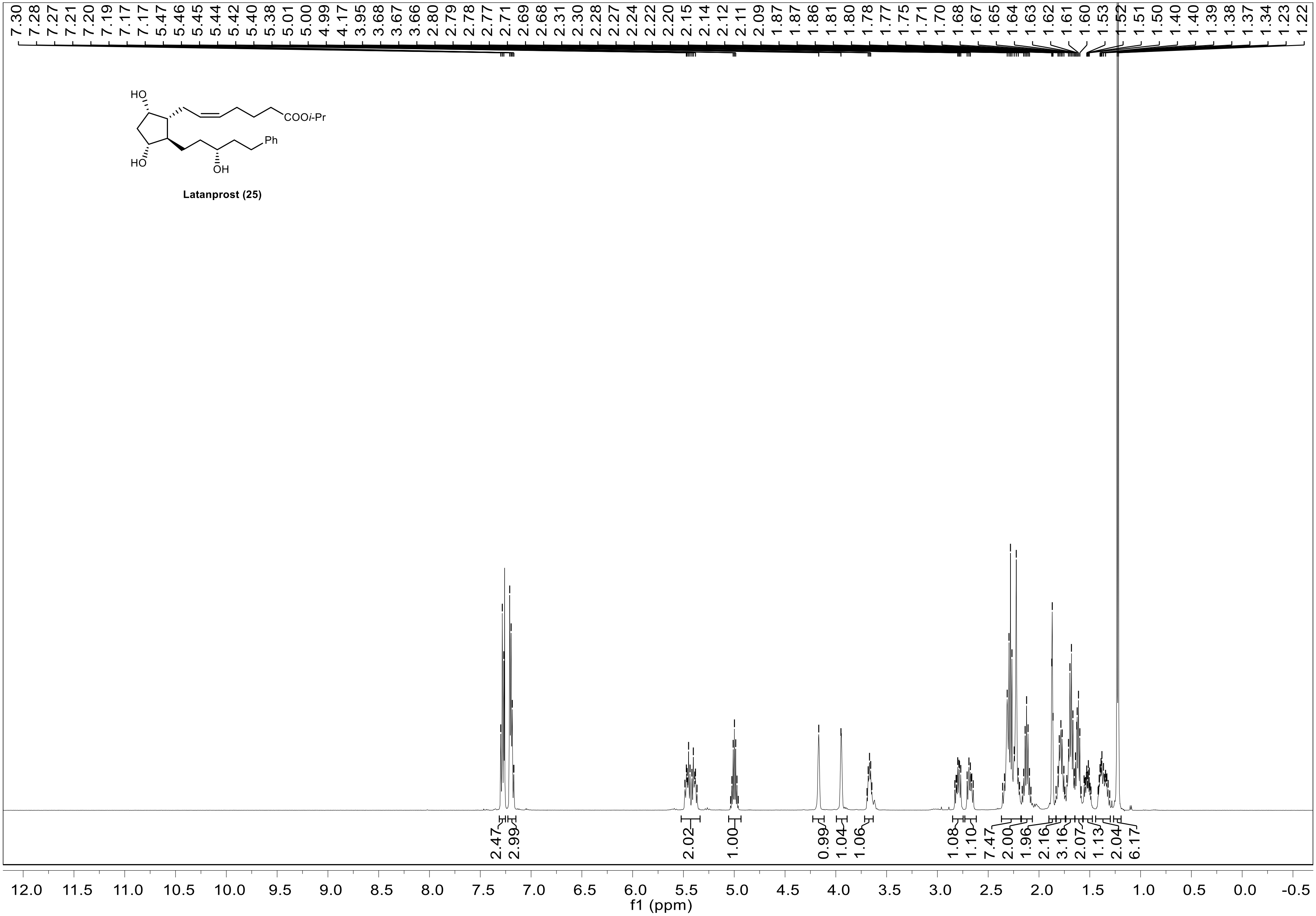

**Supplementary Figure 38. $^{13}$C NMR Spectrum of Latanprost (25) (126 MHz, $CDCl_3$)**

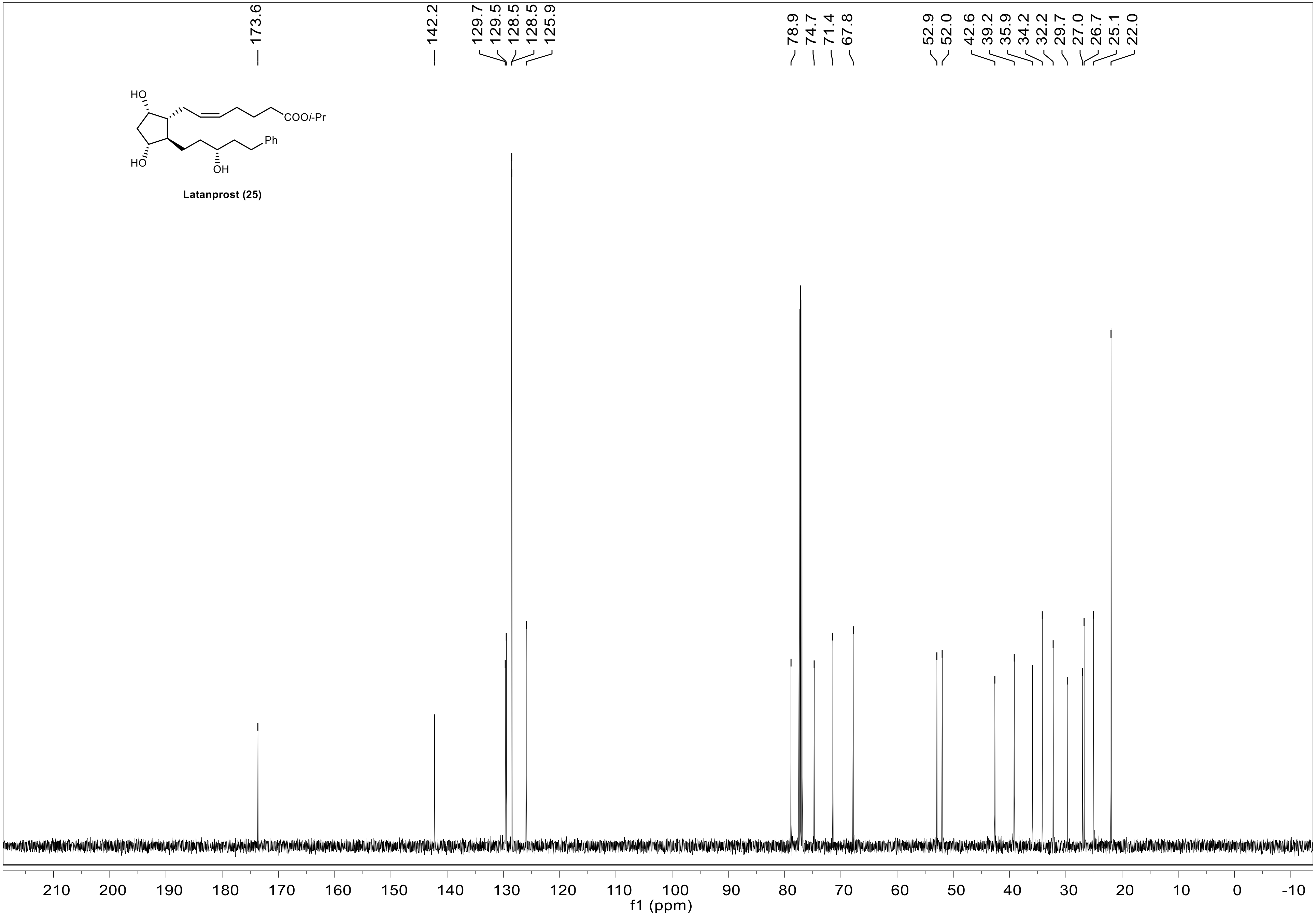

**Supplementary Figure 39. $^1$H NMR Spectrum of compound 21 (500 MHz, $CDCl_3$)**

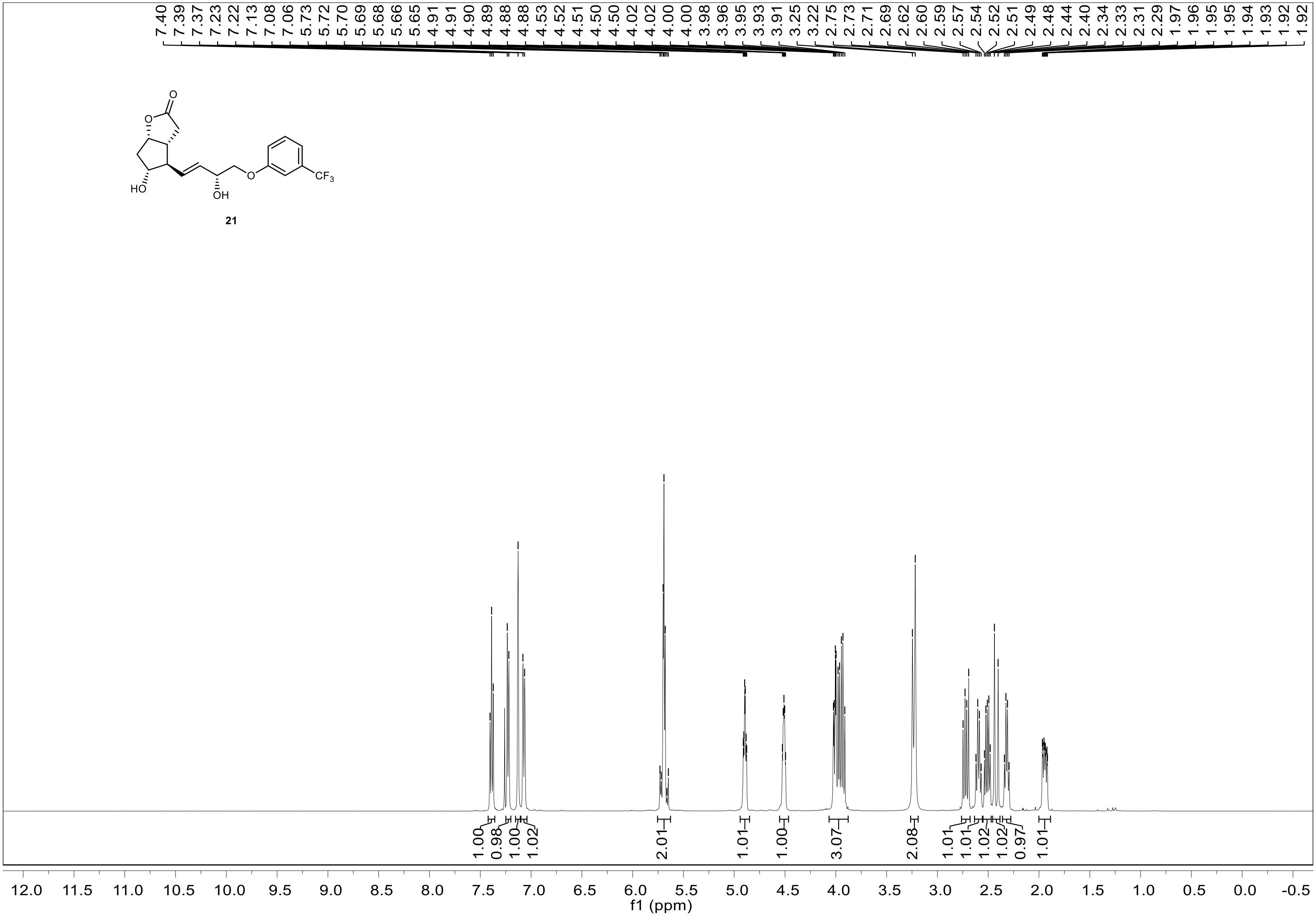

**Supplementary Figure 40. $^{13}C$ NMR Spectrum of compound 21 (126 MHz, $CDCl_3$)**

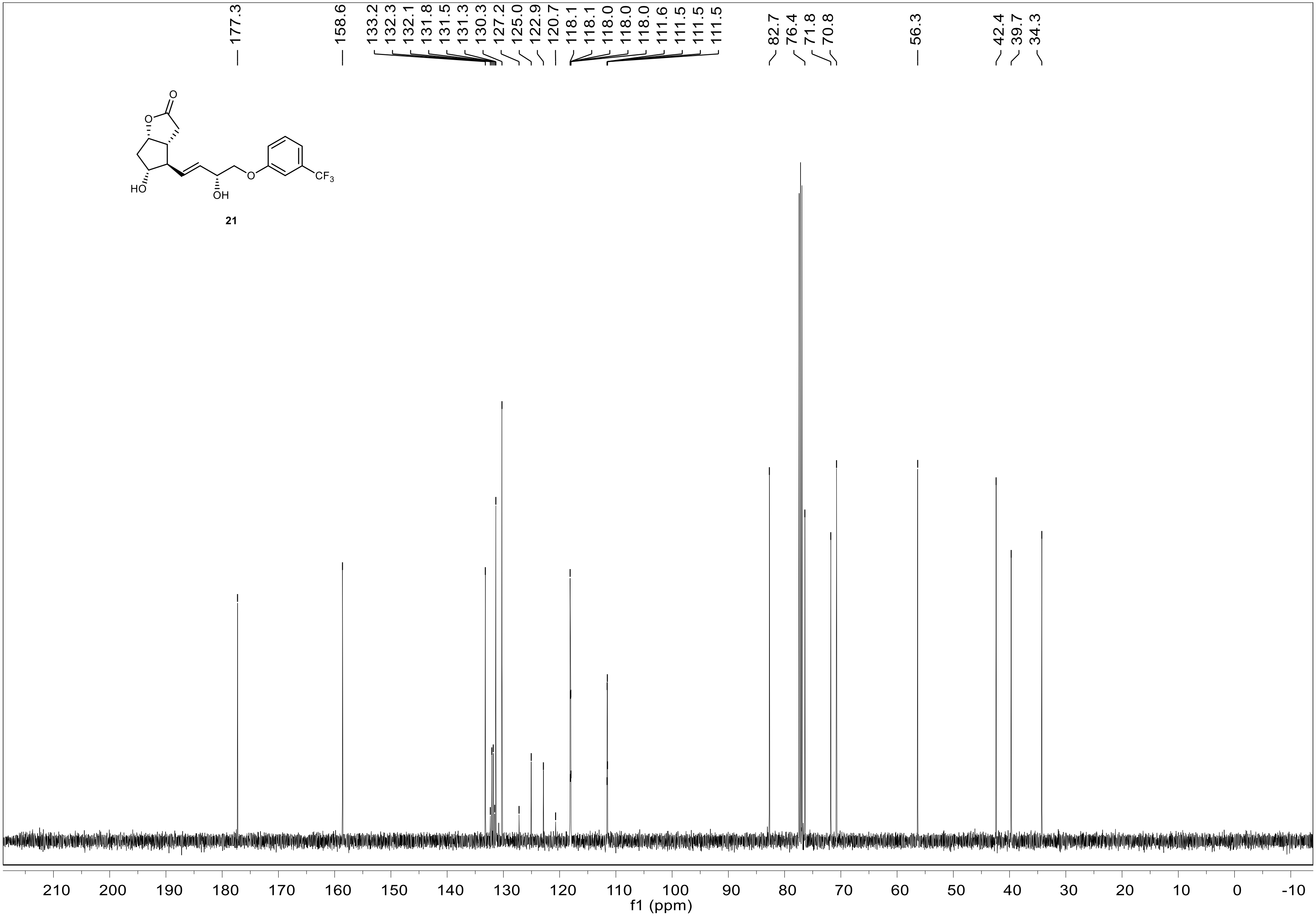

**Supplementary Figure 41. $^{19}$F NMR Spectrum of compound 21 (471 MHz, $CDCl_3$)**

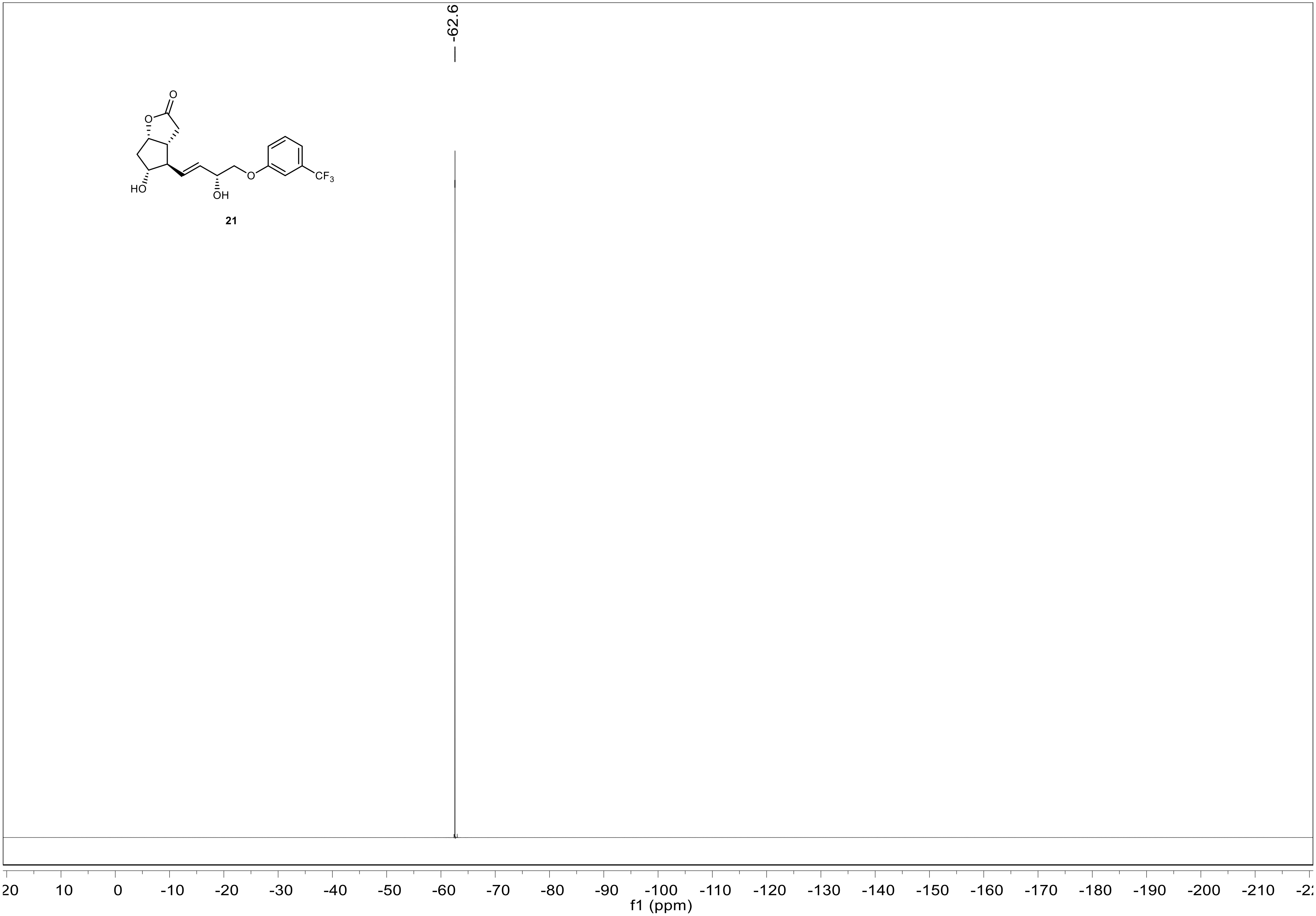

**Supplementary Figure 42. $^1$H NMR Spectrum of Fluprostenol (3) (500 MHz, $CDCl_3$)**

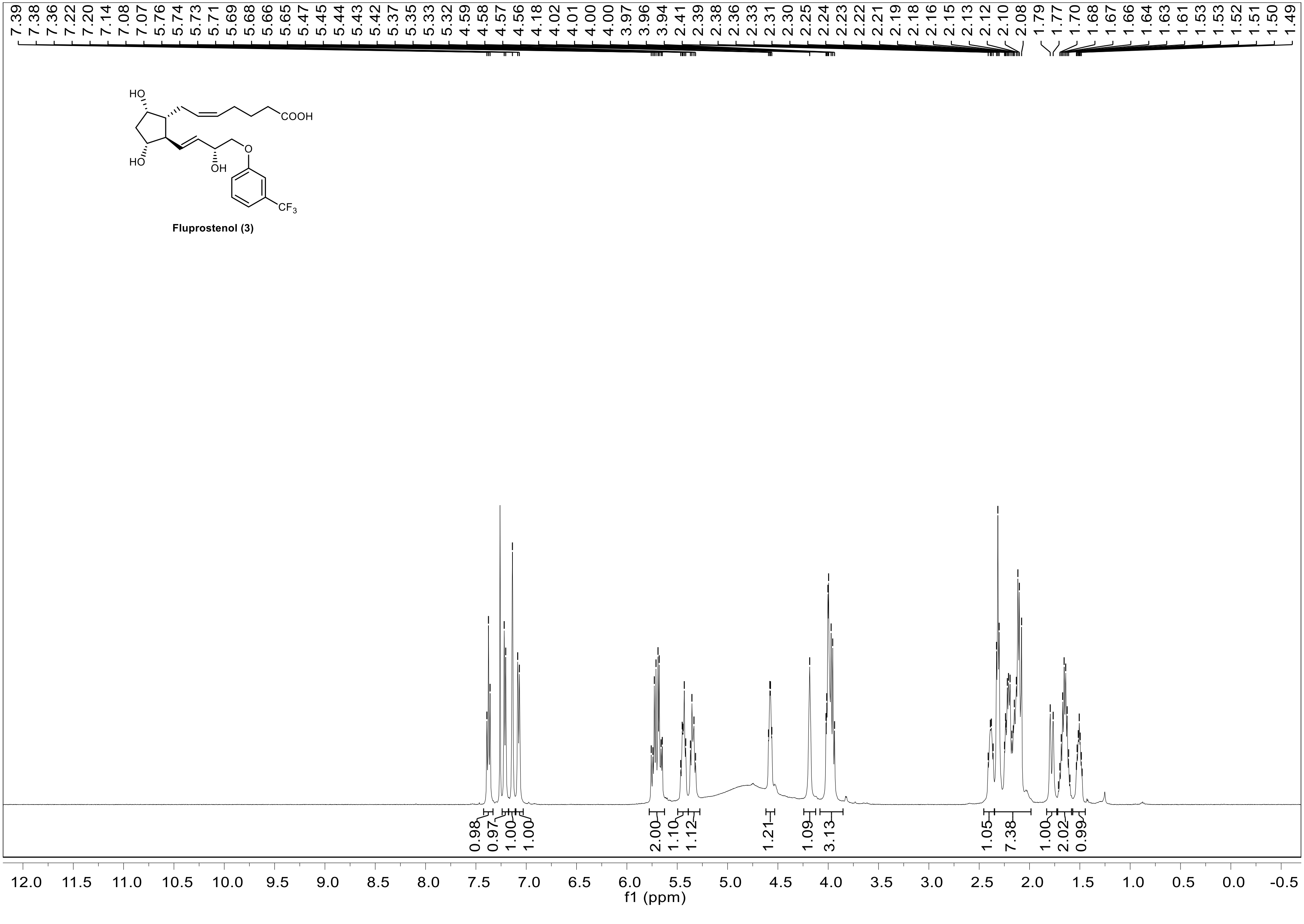

**Supplementary Figure 43. $^{13}$C NMR Spectrum of Fluprostenol (3) (126 MHz, CDCl$_3$)**

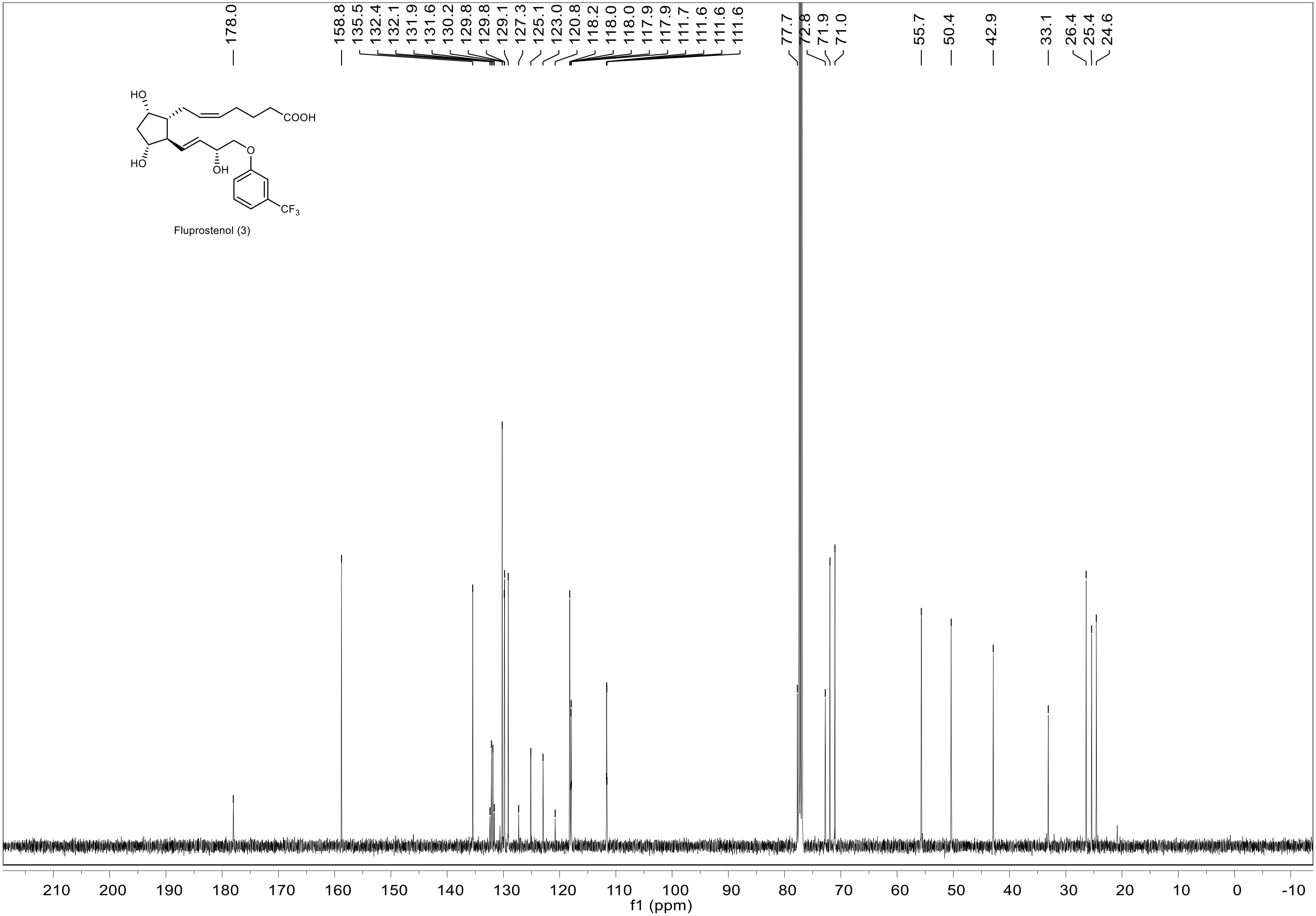

**Supplementary Figure 44. $^{19}$F NMR Spectrum of Fluprostenol (3) (471 MHz, $CDCl_3$)**

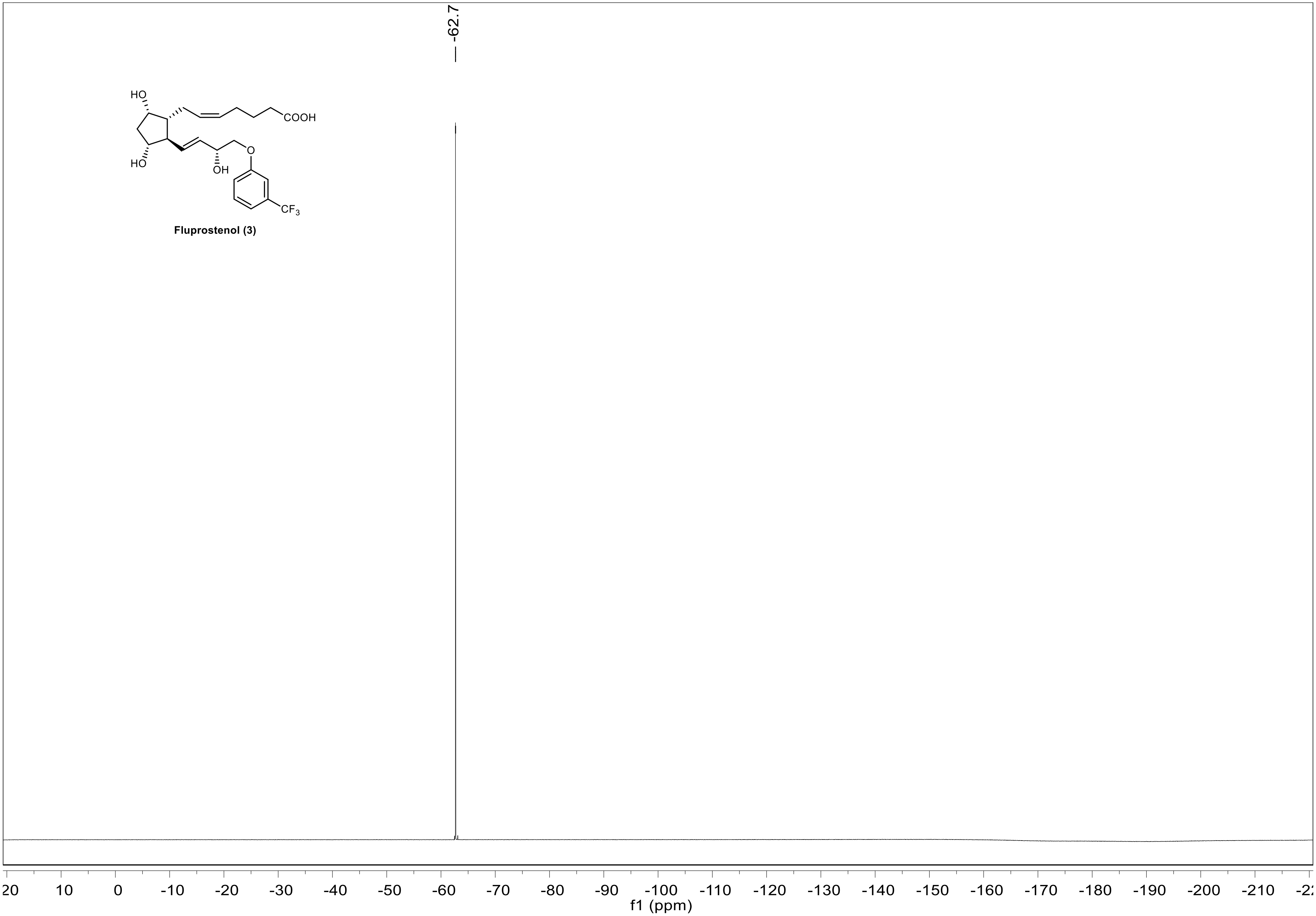

**Supplementary Figure 45. $^{1}$H NMR Spectrum of compound 22 (500 MHz, $CDCl_3$)**

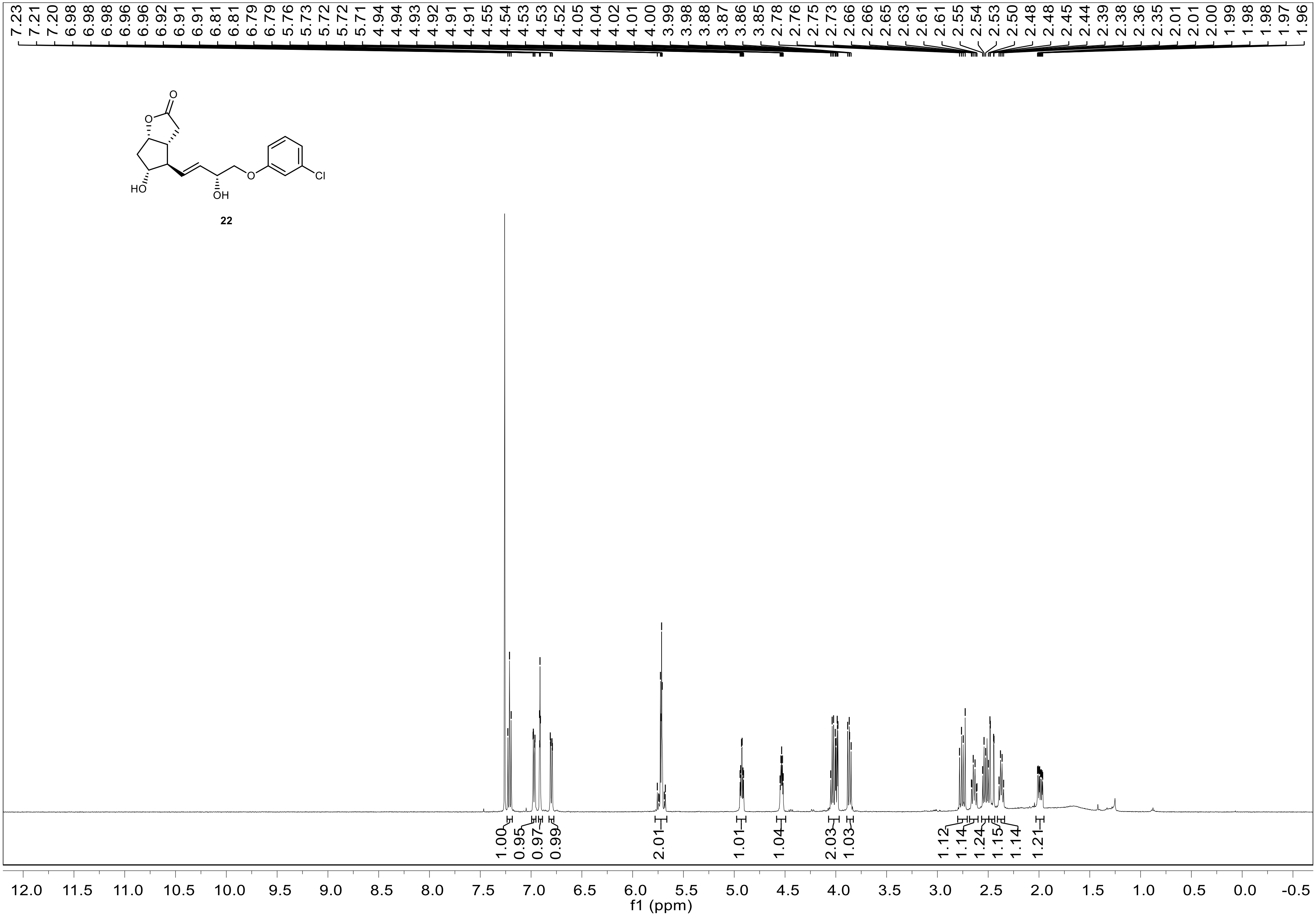

**Supplementary Figure 46. $^{13}$C NMR Spectrum of compound 22 (126 MHz, $CDCl_3$)**

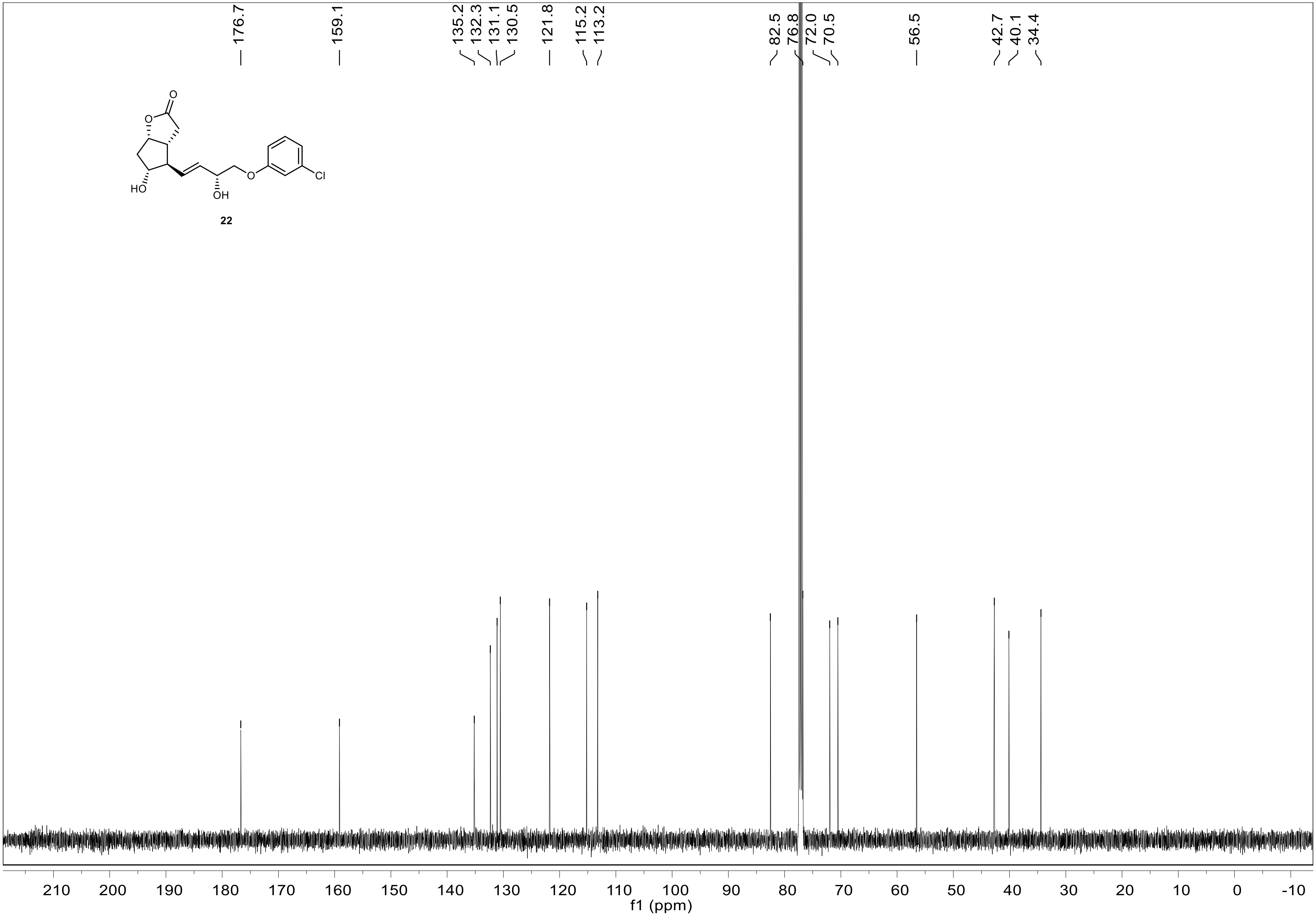

**Supplementary Figure 47. $^1$H NMR Spectrum of Cloprostenol (4) (500 MHz, $CDCl_3$)**

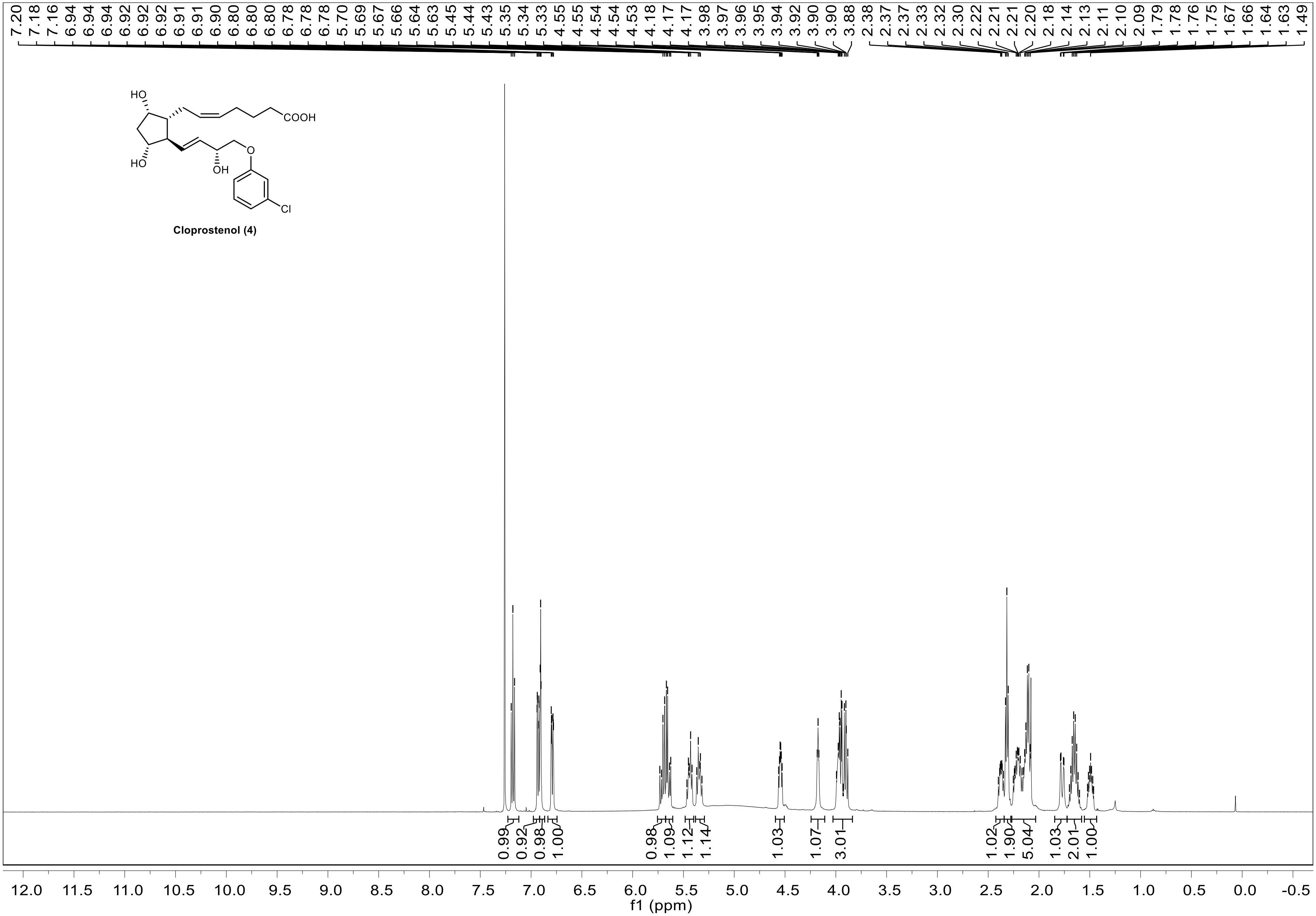

**Supplementary Figure 48. $^{13}C$ NMR Spectrum of Cloprostenol (4) (126 MHz, $CDCl_3$)**

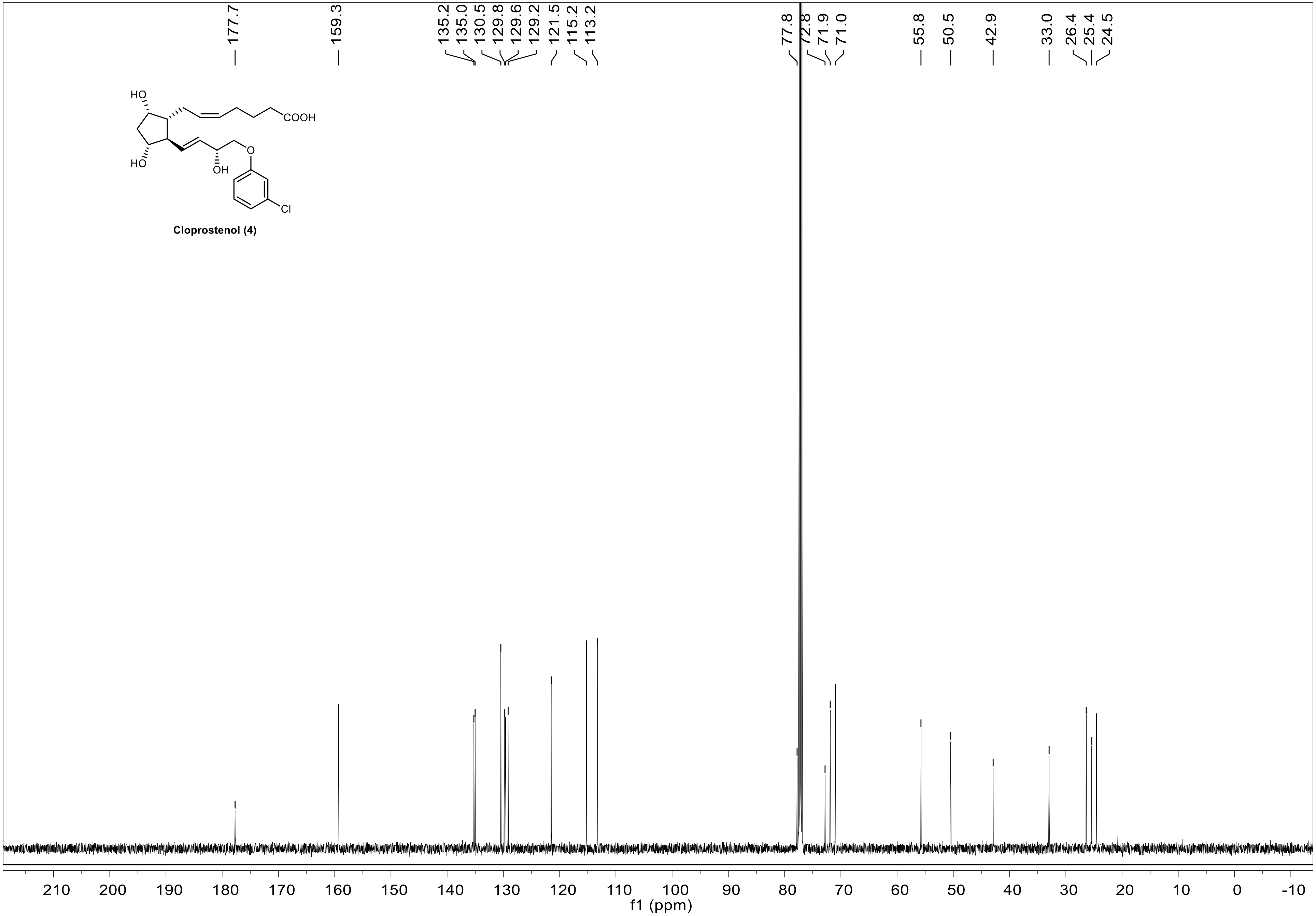

**Supplementary Figure 49. $^{1}$H NMR Spectrum of Compound 23 (400 MHz, $CDCl_3$)**

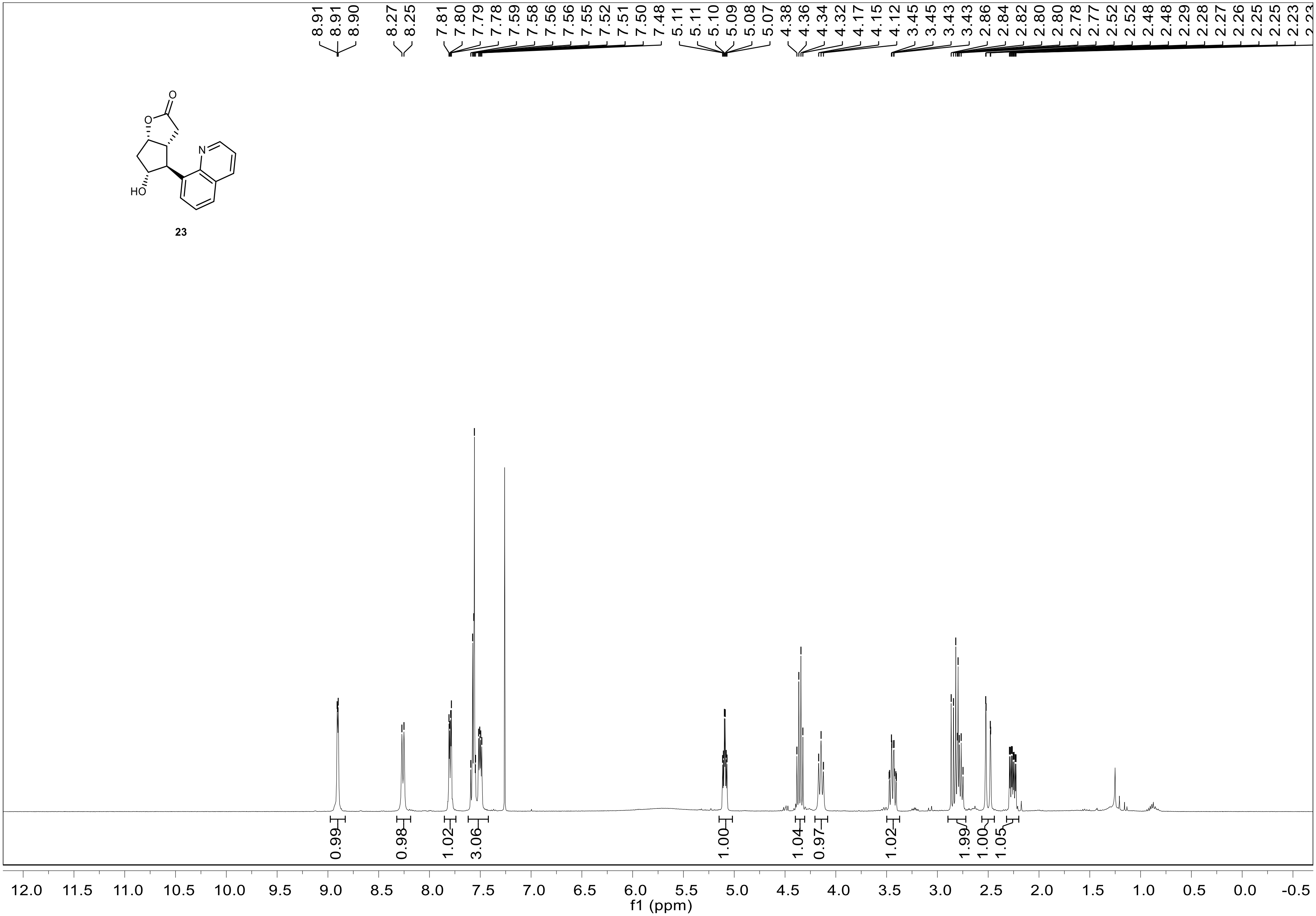

**Supplementary Figure 50. $^{13}$C NMR Spectrum of Compound 23 (101 MHz, $CDCl_3$)**

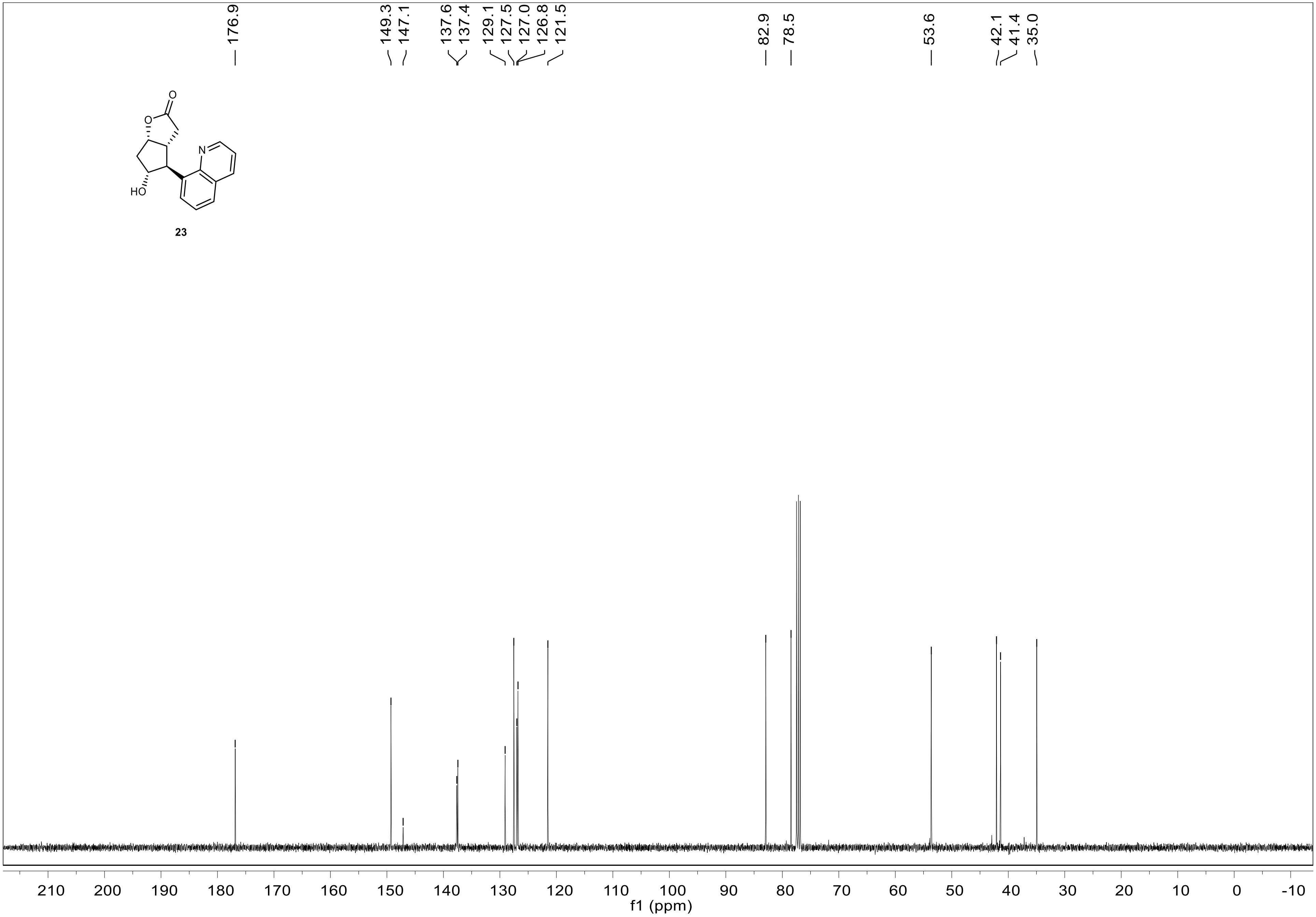

Supplementary Figure 51. $^1$H NMR Spectrum of Compound 24 (400 MHz, $CDCl_3$)

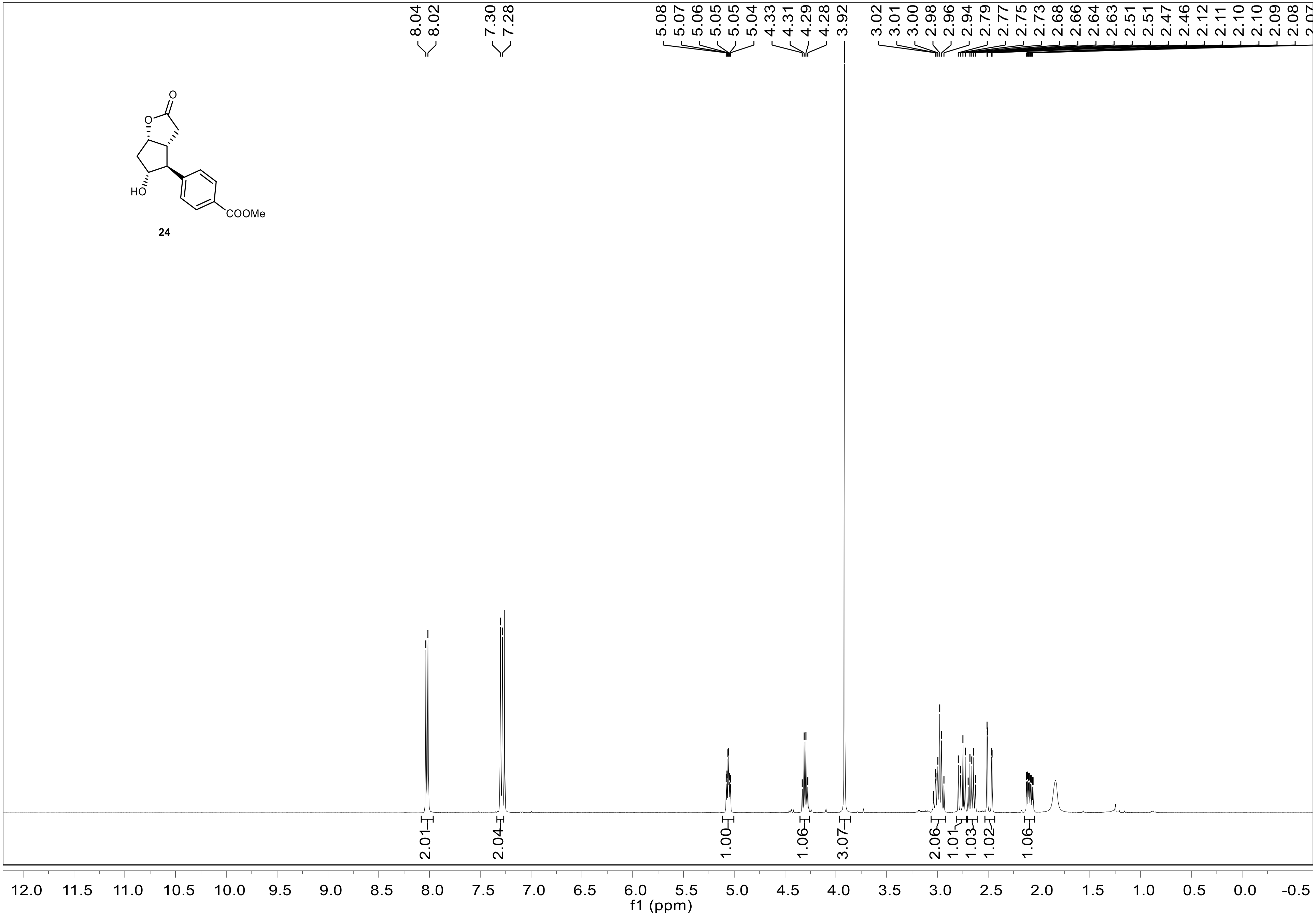

**Supplementary Figure 52. $^{13}C$ NMR Spectrum of Compound 24 (101 MHz, $CDCl_3$)**

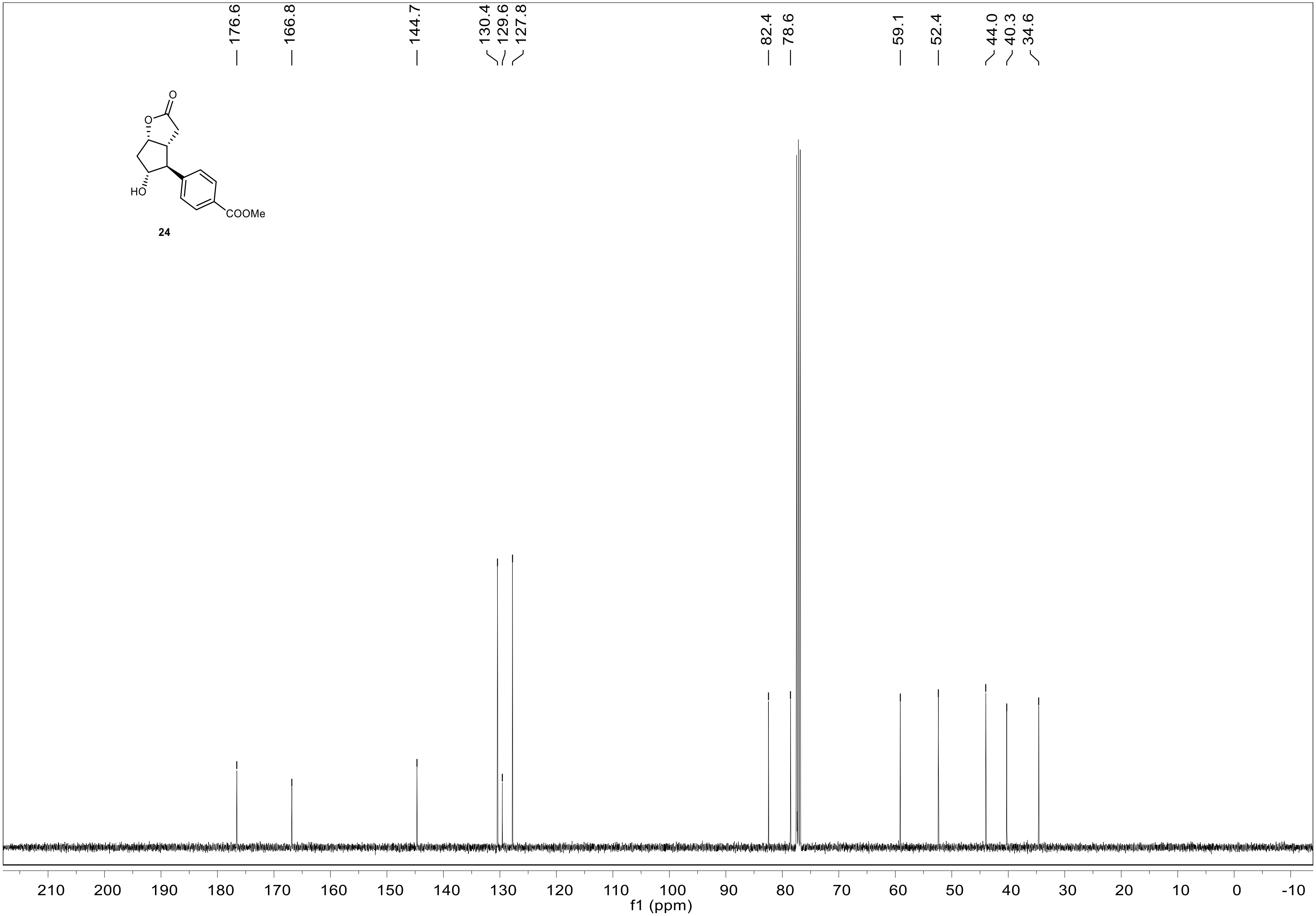

## X-ray Crystallographic Data:

## X-Ray Crystal Structure of compound 8.

Crystallographic data for the structures reported in this Article have been deposited at the Cambridge Crystallographic Data Centre, under deposition numbers CCDC 2312333 (**8**). Copies of the data can be obtained free of charge via https://www.ccdc.cam.ac.uk/structures/.

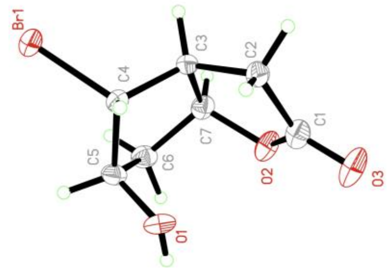


**Supplementary Figure 53.** X-Ray Crystal Structure of compound **8**.

## Crystal data and structure refinement for t_a. (8)

| | | |
|---|---|---|
| Identification code | t_a | |
| Empirical formula | C7 H9 Br O3 | |
| Formula weight | 221.05 | |
| Temperature | 173(2) K | |
| Wavelength | 1.54178 Å | |
| Crystal system | Orthorhombic | |
| Space group | $P2_12_12_1$ | |
| Unit cell dimensions | a = 6.91370(10) Å | $\alpha$= 90°. |
| | b = 9.2109(2) Å | $\beta$= 90°. |
| | c = 24.4619(5) Å | $\gamma$ = 90°. |
| Volume | 1557.77(5) Å$^3$ | |
| Z | 8 | |
| Density (calculated) | 1.885 Mg/m$^3$ | |
| Absorption coefficient | 6.855 mm$^{-1}$ | |
| F(000) | 880 | |
| Crystal size | 0.180 x 0.160 x 0.150 mm$^3$ | |
| Theta range for data collection | 5.131 to 68.313°. | |
| Index ranges | -8<=h<=8, -11<=k<=10, -28<=l<=29 | |
| Reflections collected | 14715 | |
| Independent reflections | 2841 [R(int) = 0.0331] | |
| Completeness to theta = 67.679° | 99.9 % | |
| Absorption correction | Semi-empirical from equivalents | |

| | |
|---|---|
| Max. and min. transmission | 0.7531 and 0.5882 |
| Refinement method | Full-matrix least-squares on $F^2$ |
| Data / restraints / parameters | 2841 / 1 / 205 |
| Goodness-of-fit on $F^2$ | 1.093 |
| Final R indices [I>2sigma(I)] | R1 = 0.0181, wR2 = 0.0468 |
| R indices (all data) | R1 = 0.0192, wR2 = 0.0470 |
| Absolute structure parameter | 0.012(6) |
| Extinction coefficient | n/a |
| Largest diff. peak and hole | 0.330 and -0.653 e.Å$^{-3}$ |

**Supplementary Table 1. Atomic coordinates ( x $10^4$) and equivalent isotropic displacement parameters (Å$^2$x $10^3$) for t_a. U(eq) is defined as one third of the trace of the orthogonalized $U^{ij}$ tensor.**

| | x | y | z | U(eq) |
|---|---|---|---|---|
| Br(1) | 7521(1) | 12218(1) | 3428(1) | 27(1) |
| Br(2) | 2152(1) | 1846(1) | 4518(1) | 26(1) |
| O(1) | 6859(3) | 8040(2) | 3929(1) | 26(1) |
| O(2) | 5789(3) | 7298(2) | 2780(1) | 27(1) |
| O(3) | 8090(4) | 5605(2) | 2803(1) | 37(1) |
| O(4) | 4642(3) | 5920(2) | 4492(1) | 23(1) |
| O(5) | 1342(3) | 6852(2) | 3852(1) | 21(1) |
| O(6) | 1016(3) | 8632(2) | 4453(1) | 24(1) |
| C(1) | 7652(5) | 6879(3) | 2787(1) | 24(1) |
| C(10) | 645(5) | 4756(3) | 4410(1) | 18(1) |
| C(11) | 2420(5) | 3949(3) | 4636(1) | 18(1) |
| C(12) | 4143(5) | 4499(4) | 4302(1) | 19(1) |
| C(13) | 3289(5) | 4678(3) | 3732(1) | 21(1) |
| C(14) | 1269(5) | 5265(3) | 3828(1) | 20(1) |
| C(2) | 8985(5) | 8157(4) | 2760(1) | 23(1) |
| C(3) | 7692(5) | 9482(3) | 2853(1) | 19(1) |
| C(4) | 7843(5) | 10101(3) | 3439(1) | 19(1) |
| C(5) | 6154(5) | 9428(3) | 3755(1) | 21(1) |
| C(6) | 4563(5) | 9309(4) | 3328(1) | 24(1) |
| C(7) | 5605(5) | 8882(3) | 2807(1) | 22(1) |
| C(8) | 865(4) | 7360(3) | 4349(1) | 18(1) |
| C(9) | 188(5) | 6159(3) | 4712(1) | 22(1) |

## Supplementary Table 2. Bond lengths [Å] and angles [°] for t_a.

| | |
|---|---|
| Br(1)-C(4) | 1.963(3) |
| Br(2)-C(11) | 1.966(3) |
| O(1)-C(5) | 1.433(4) |
| O(2)-C(1) | 1.344(4) |
| O(2)-C(7) | 1.466(4) |
| O(3)-C(1) | 1.212(4) |
| O(4)-C(12) | 1.431(4) |
| O(5)-C(8) | 1.342(3) |
| O(5)-C(14) | 1.464(4) |
| O(6)-C(8) | 1.204(4) |
| C(1)-C(2) | 1.497(5) |
| C(10)-C(9) | 1.521(4) |
| C(10)-C(11) | 1.538(4) |
| C(10)-C(14) | 1.559(4) |
| C(11)-C(12) | 1.531(4) |
| C(12)-C(13) | 1.525(4) |
| C(13)-C(14) | 1.516(4) |
| C(2)-C(3) | 1.530(4) |
| C(3)-C(4) | 1.546(3) |
| C(3)-C(7) | 1.549(5) |
| C(4)-C(5) | 1.531(4) |
| C(5)-C(6) | 1.521(4) |
| C(6)-C(7) | 1.517(4) |
| C(8)-C(9) | 1.494(4) |
| | |
| C(1)-O(2)-C(7) | 111.6(3) |
| C(8)-O(5)-C(14) | 112.0(2) |
| O(3)-C(1)-O(2) | 121.2(3) |
| O(3)-C(1)-C(2) | 127.5(3) |
| O(2)-C(1)-C(2) | 111.3(3) |
| C(9)-C(10)-C(11) | 113.7(2) |
| C(9)-C(10)-C(14) | 104.2(2) |
| C(11)-C(10)-C(14) | 104.6(2) |
| C(12)-C(11)-C(10) | 105.6(2) |
| C(12)-C(11)-Br(2) | 108.7(2) |
| C(10)-C(11)-Br(2) | 110.4(2) |
| O(4)-C(12)-C(13) | 106.9(2) |

| | |
|---|---|
| O(4)-C(12)-C(11) | 108.5(2) |
| C(13)-C(12)-C(11) | 102.9(2) |
| C(14)-C(13)-C(12) | 104.6(2) |
| O(5)-C(14)-C(13) | 109.3(2) |
| O(5)-C(14)-C(10) | 105.9(2) |
| C(13)-C(14)-C(10) | 106.9(3) |
| C(1)-C(2)-C(3) | 105.1(3) |
| C(2)-C(3)-C(4) | 113.2(3) |
| C(2)-C(3)-C(7) | 104.4(2) |
| C(4)-C(3)-C(7) | 105.2(2) |
| C(5)-C(4)-C(3) | 105.5(2) |
| C(5)-C(4)-Br(1) | 108.8(2) |
| C(3)-C(4)-Br(1) | 110.29(17) |
| O(1)-C(5)-C(6) | 112.7(3) |
| O(1)-C(5)-C(4) | 104.6(2) |
| C(6)-C(5)-C(4) | 103.5(2) |
| C(7)-C(6)-C(5) | 104.6(3) |
| O(2)-C(7)-C(6) | 109.7(3) |
| O(2)-C(7)-C(3) | 106.1(3) |
| C(6)-C(7)-C(3) | 106.8(2) |
| O(6)-C(8)-O(5) | 120.6(3) |
| O(6)-C(8)-C(9) | 128.4(3) |
| O(5)-C(8)-C(9) | 110.9(2) |
| C(8)-C(9)-C(10) | 106.0(2) |

Symmetry transformations used to generate equivalent atoms:

**Supplementary Table 3. Anisotropic displacement parameters ($Å^2$x $10^3$) for t_a. The anisotropic displacement factor exponent takes the form: $-2p^2[ h^2 a^{*2}U^{11} + ... + 2 h k a^* b^* U^{12} ]$**

| | $U^{11}$ | $U^{22}$ | $U^{33}$ | $U^{23}$ | $U^{13}$ | $U^{12}$ |
|---|---|---|---|---|---|---|
| Br(1) | 34(1) | 17(1) | 31(1) | -3(1) | 4(1) | -2(1) |
| Br(2) | 32(1) | 11(1) | 34(1) | 1(1) | 2(1) | 0(1) |
| O(1) | 26(1) | 25(1) | 25(1) | 11(1) | 1(1) | -4(1) |
| O(2) | 26(1) | 17(1) | 37(1) | -3(1) | -9(1) | -1(1) |
| O(3) | 40(2) | 18(1) | 52(1) | -5(1) | -16(1) | 5(1) |
| O(4) | 26(1) | 19(1) | 22(1) | -1(1) | -4(1) | -8(1) |
| O(5) | 31(1) | 14(1) | 18(1) | 1(1) | 2(1) | 1(1) |

| | | | | | | |
|---|---|---|---|---|---|---|
| O(6) | 32(1) | 14(1) | 28(1) | -1(1) | -1(1) | -1(1) |
| C(1) | 26(2) | 23(1) | 24(1) | -2(1) | -9(1) | 1(2) |
| C(10) | 19(2) | 15(2) | 20(2) | 1(1) | 2(1) | -3(1) |
| C(11) | 23(2) | 12(1) | 18(1) | -2(1) | 0(1) | -1(1) |
| C(12) | 21(2) | 16(2) | 21(1) | -3(1) | 1(1) | 0(1) |
| C(13) | 29(2) | 18(1) | 16(1) | -3(1) | 4(1) | -1(1) |
| C(14) | 28(2) | 12(1) | 19(1) | -3(1) | -4(1) | -2(1) |
| C(2) | 23(2) | 19(2) | 26(1) | -1(1) | 6(1) | 3(2) |
| C(3) | 24(2) | 16(1) | 16(1) | 2(1) | 3(1) | 1(1) |
| C(4) | 22(2) | 15(1) | 19(1) | 1(1) | 0(1) | 0(1) |
| C(5) | 21(2) | 23(2) | 20(1) | 3(1) | 4(1) | 0(1) |
| C(6) | 20(2) | 25(2) | 27(2) | 4(1) | 3(1) | 1(1) |
| C(7) | 25(2) | 19(2) | 21(1) | 2(1) | -5(1) | 3(1) |
| C(8) | 17(1) | 19(2) | 18(1) | 0(1) | -2(1) | 1(1) |
| C(9) | 25(2) | 17(2) | 23(1) | 1(1) | 7(1) | 4(1) |

**Supplementary Table 4. Hydrogen coordinates ( x $10^4$) and isotropic displacement parameters (Å$^2$x 10 $^3$) for t_a.**

| | x | y | z | U(eq) |
|---|---|---|---|---|
| H(1) | 6070(70) | 7540(50) | 4056(16) | 38 |
| H(2) | 5020(60) | 5970(40) | 4784(16) | 34 |
| H(10) | -512 | 4106 | 4393 | 21 |
| H(11) | 2603 | 4167 | 5033 | 21 |
| H(12) | 5264 | 3814 | 4310 | 23 |
| H(13A) | 4066 | 5367 | 3512 | 25 |
| H(13B) | 3238 | 3734 | 3539 | 25 |
| H(14) | 344 | 4921 | 3542 | 24 |
| H(2A) | 9623 | 8211 | 2398 | 27 |
| H(2B) | 9993 | 8092 | 3046 | 27 |
| H(3) | 7941 | 10251 | 2573 | 23 |
| H(4) | 9106 | 9829 | 3610 | 22 |
| H(5) | 5753 | 10046 | 4071 | 25 |
| H(6A) | 3605 | 8561 | 3433 | 29 |
| H(6B) | 3889 | 10250 | 3282 | 29 |
| H(7) | 4932 | 9274 | 2476 | 26 |
| H(9A) | -1219 | 6241 | 4780 | 26 |

| | | | | |
|---|---|---|---|---|
| H(9B) | 874 | 6190 | 5067 | 26 |

**Supplementary Table 5. Torsion angles [°] for t_a.**

| | |
|---|---|
| C(7)-O(2)-C(1)-O(3) | 175.2(3) |
| C(7)-O(2)-C(1)-C(2) | -6.0(3) |
| C(9)-C(10)-C(11)-C(12) | -92.0(3) |
| C(14)-C(10)-C(11)-C(12) | 21.0(3) |
| C(9)-C(10)-C(11)-Br(2) | 150.6(2) |
| C(14)-C(10)-C(11)-Br(2) | -96.3(2) |
| C(10)-C(11)-C(12)-O(4) | 76.1(3) |
| Br(2)-C(11)-C(12)-O(4) | -165.43(19) |
| C(10)-C(11)-C(12)-C(13) | -36.9(3) |
| Br(2)-C(11)-C(12)-C(13) | 81.5(2) |
| O(4)-C(12)-C(13)-C(14) | -75.7(3) |
| C(11)-C(12)-C(13)-C(14) | 38.5(3) |
| C(8)-O(5)-C(14)-C(13) | -114.9(3) |
| C(8)-O(5)-C(14)-C(10) | 0.0(3) |
| C(12)-C(13)-C(14)-O(5) | 88.4(3) |
| C(12)-C(13)-C(14)-C(10) | -25.7(3) |
| C(9)-C(10)-C(14)-O(5) | 6.0(3) |
| C(11)-C(10)-C(14)-O(5) | -113.6(3) |
| C(9)-C(10)-C(14)-C(13) | 122.5(3) |
| C(11)-C(10)-C(14)-C(13) | 2.9(3) |
| O(3)-C(1)-C(2)-C(3) | -170.0(3) |
| O(2)-C(1)-C(2)-C(3) | 11.3(3) |
| C(1)-C(2)-C(3)-C(4) | 102.2(3) |
| C(1)-C(2)-C(3)-C(7) | -11.6(3) |
| C(2)-C(3)-C(4)-C(5) | -96.0(3) |
| C(7)-C(3)-C(4)-C(5) | 17.3(3) |
| C(2)-C(3)-C(4)-Br(1) | 146.7(2) |
| C(7)-C(3)-C(4)-Br(1) | -100.0(2) |
| C(3)-C(4)-C(5)-O(1) | 84.0(3) |
| Br(1)-C(4)-C(5)-O(1) | -157.68(18) |
| C(3)-C(4)-C(5)-C(6) | -34.3(3) |
| Br(1)-C(4)-C(5)-C(6) | 84.1(2) |
| O(1)-C(5)-C(6)-C(7) | -74.4(3) |
| C(4)-C(5)-C(6)-C(7) | 38.1(3) |
| C(1)-O(2)-C(7)-C(6) | -116.9(3) |

| | |
|---|---|
| C(1)-O(2)-C(7)-C(3) | -1.9(3) |
| C(5)-C(6)-C(7)-O(2) | 87.1(3) |
| C(5)-C(6)-C(7)-C(3) | -27.4(3) |
| C(2)-C(3)-C(7)-O(2) | 8.6(3) |
| C(4)-C(3)-C(7)-O(2) | -110.8(2) |
| C(2)-C(3)-C(7)-C(6) | 125.5(2) |
| C(4)-C(3)-C(7)-C(6) | 6.1(3) |
| C(14)-O(5)-C(8)-O(6) | 173.4(3) |
| C(14)-O(5)-C(8)-C(9) | -6.3(3) |
| O(6)-C(8)-C(9)-C(10) | -169.6(3) |
| O(5)-C(8)-C(9)-C(10) | 10.1(4) |
| C(11)-C(10)-C(9)-C(8) | 103.9(3) |
| C(14)-C(10)-C(9)-C(8) | -9.4(3) |

Symmetry transformations used to generate equivalent atoms:

**Supplementary Table 6. Hydrogen bonds for t_a [Å and °].**

| D-H...A | d(D-H) | d(H...A) | d(D...A) | <(DHA) |
|---|---|---|---|---|

**X-Ray Crystal Structure of compound 21**

Crystallographic data for the structures reported in this Article have been deposited at the Cambridge Crystallographic Data Centre, under deposition numbers CCDC 2319091 (**21**). Copies of the data can be obtained free of charge via https://www.ccdc.cam.ac.uk/structures/.

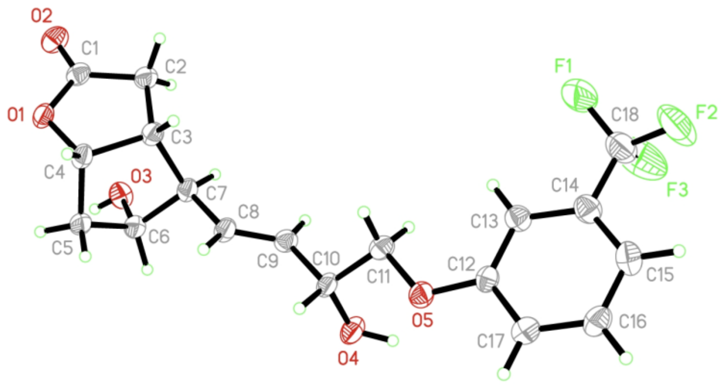


**Supplementary Figure 54.** X-Ray Crystal Structure of compound **21**.

## Crystal data and structure refinement for t_a (21).

| | | |
|---|---|---|
| Identification code | t_a | |
| Empirical formula | C18 H19 F3 O5 | |
| Formula weight | 372.33 | |
| Temperature | 173(2) K | |
| Wavelength | 1.54178 Å | |
| Crystal system | Monoclinic | |
| Space group | $P2_1$ | |
| Unit cell dimensions | a = 6.0146(3) Å | $\alpha$= 90°. |
| | b = 9.8573(5) Å | $\beta$= 92.462(3)°. |
| | c = 14.6641(8) Å | $\gamma$ = 90°. |
| Volume | 868.60(8) $Å^3$ | |
| Z | 2 | |
| Density (calculated) | 1.424 $Mg/m^3$ | |
| Absorption coefficient | 1.069 $mm^{-1}$ | |
| F(000) | 388 | |
| Crystal size | 0.160 x 0.150 x 0.140 $mm^3$ | |
| Theta range for data collection | 3.016 to 68.283°. | |
| Index ranges | -7<=h<=7, -11<=k<=11, -17<=l<=17 | |
| Reflections collected | 10631 | |
| Independent reflections | 3140 [R(int) = 0.0553] | |
| Completeness to theta = 67.679° | 100.0 % | |
| Absorption correction | Semi-empirical from equivalents | |
| Max. and min. transmission | 0.7531 and 0.7167 | |
| Refinement method | Full-matrix least-squares on $F^2$ | |
| Data / restraints / parameters | 3140 / 1 / 242 | |
| Goodness-of-fit on $F^2$ | 1.082 | |
| Final R indices [I>2sigma(I)] | R1 = 0.0428, wR2 = 0.1112 | |
| R indices (all data) | R1 = 0.0474, wR2 = 0.1151 | |
| Absolute structure parameter | 0.00(10) | |
| Extinction coefficient | 0.012(2) | |
| Largest diff. peak and hole | 0.230 and -0.199 e.$Å^{-3}$ | |

**Supplementary Table 7. Atomic coordinates ( x $10^4$) and equivalent isotropic displacement parameters (Å$^2$x $10^3$) for t_a. U(eq) is defined as one third of the trace of the orthogonalized $U^{ij}$ tensor.**

| | x | y | z | U(eq) |
|---|---|---|---|---|
| F(1) | 6789(6) | 8096(4) | 5871(2) | 82(1) |
| F(2) | 9524(5) | 9341(4) | 6305(2) | 75(1) |
| F(3) | 6731(6) | 10182(4) | 5562(2) | 88(1) |
| O(1) | -1212(3) | 42(2) | 1295(2) | 27(1) |
| O(2) | -4269(3) | -148(3) | 2075(2) | 34(1) |
| O(3) | -2769(4) | 2782(3) | 546(2) | 31(1) |
| O(4) | 4638(4) | 7349(3) | 1203(2) | 33(1) |
| O(5) | 8163(3) | 7096(2) | 2534(2) | 28(1) |
| C(1) | -2551(5) | 447(3) | 1947(2) | 26(1) |
| C(2) | -1579(5) | 1633(4) | 2468(2) | 28(1) |
| C(3) | 549(5) | 2014(3) | 1990(2) | 25(1) |
| C(4) | 769(5) | 902(3) | 1248(2) | 26(1) |
| C(5) | 762(5) | 1643(3) | 337(2) | 27(1) |
| C(6) | -423(5) | 2973(4) | 499(2) | 27(1) |
| C(7) | 422(5) | 3386(3) | 1467(2) | 24(1) |
| C(8) | 2637(5) | 4099(3) | 1448(2) | 26(1) |
| C(9) | 2965(5) | 5347(3) | 1749(2) | 25(1) |
| C(10) | 5107(5) | 6107(3) | 1696(2) | 26(1) |
| C(11) | 6087(5) | 6413(4) | 2640(2) | 27(1) |
| C(12) | 9056(5) | 7851(3) | 3236(2) | 24(1) |
| C(13) | 8094(5) | 8018(4) | 4081(2) | 28(1) |
| C(14) | 9162(5) | 8850(4) | 4731(2) | 32(1) |
| C(15) | 11147(6) | 9494(4) | 4567(3) | 38(1) |
| C(16) | 12112(5) | 9301(4) | 3726(3) | 32(1) |
| C(17) | 11062(5) | 8489(4) | 3067(2) | 28(1) |
| C(18) | 8082(7) | 9115(5) | 5614(3) | 45(1) |

**Supplementary Table 8. Bond lengths [Å] and angles [°] for t_a.**

| | |
|---|---|
| F(1)-C(18) | 1.335(5) |
| F(2)-C(18) | 1.325(5) |
| F(3)-C(18) | 1.329(6) |
| O(1)-C(1) | 1.338(4) |
| O(1)-C(4) | 1.466(4) |
| O(2)-C(1) | 1.210(4) |
| O(3)-C(6) | 1.428(4) |
| O(4)-C(10) | 1.443(4) |
| O(5)-C(12) | 1.363(4) |
| O(5)-C(11) | 1.433(4) |
| C(1)-C(2) | 1.501(5) |
| C(2)-C(3) | 1.532(4) |
| C(3)-C(4) | 1.555(5) |
| C(3)-C(7) | 1.556(5) |
| C(4)-C(5) | 1.523(5) |
| C(5)-C(6) | 1.516(5) |
| C(6)-C(7) | 1.541(4) |
| C(7)-C(8) | 1.507(4) |
| C(8)-C(9) | 1.319(5) |
| C(9)-C(10) | 1.496(4) |
| C(10)-C(11) | 1.513(5) |
| C(12)-C(17) | 1.392(4) |
| C(12)-C(13) | 1.400(4) |
| C(13)-C(14) | 1.393(5) |
| C(14)-C(15) | 1.382(5) |
| C(14)-C(18) | 1.495(5) |
| C(15)-C(16) | 1.399(5) |
| C(16)-C(17) | 1.386(5) |
| | |
| C(1)-O(1)-C(4) | 112.1(2) |
| C(12)-O(5)-C(11) | 119.4(2) |
| O(2)-C(1)-O(1) | 120.7(3) |
| O(2)-C(1)-C(2) | 128.0(3) |
| O(1)-C(1)-C(2) | 111.3(3) |
| C(1)-C(2)-C(3) | 105.8(3) |
| C(2)-C(3)-C(4) | 104.2(3) |
| C(2)-C(3)-C(7) | 114.5(3) |

| | |
|---|---|
| C(4)-C(3)-C(7) | 105.8(2) |
| O(1)-C(4)-C(5) | 110.3(3) |
| O(1)-C(4)-C(3) | 106.3(2) |
| C(5)-C(4)-C(3) | 106.2(3) |
| C(6)-C(5)-C(4) | 105.1(3) |
| O(3)-C(6)-C(5) | 111.5(3) |
| O(3)-C(6)-C(7) | 106.1(2) |
| C(5)-C(6)-C(7) | 103.6(3) |
| C(8)-C(7)-C(6) | 111.3(3) |
| C(8)-C(7)-C(3) | 112.9(3) |
| C(6)-C(7)-C(3) | 103.4(3) |
| C(9)-C(8)-C(7) | 123.2(3) |
| C(8)-C(9)-C(10) | 124.5(3) |
| O(4)-C(10)-C(9) | 107.5(3) |
| O(4)-C(10)-C(11) | 110.4(3) |
| C(9)-C(10)-C(11) | 110.8(3) |
| O(5)-C(11)-C(10) | 107.5(2) |
| O(5)-C(12)-C(17) | 115.3(3) |
| O(5)-C(12)-C(13) | 124.7(3) |
| C(17)-C(12)-C(13) | 120.0(3) |
| C(14)-C(13)-C(12) | 118.6(3) |
| C(15)-C(14)-C(13) | 121.9(3) |
| C(15)-C(14)-C(18) | 118.8(3) |
| C(13)-C(14)-C(18) | 119.3(3) |
| C(14)-C(15)-C(16) | 119.0(3) |
| C(17)-C(16)-C(15) | 120.0(3) |
| C(16)-C(17)-C(12) | 120.6(3) |
| F(2)-C(18)-F(3) | 106.8(4) |
| F(2)-C(18)-F(1) | 106.3(4) |
| F(3)-C(18)-F(1) | 104.4(3) |
| F(2)-C(18)-C(14) | 113.4(3) |
| F(3)-C(18)-C(14) | 112.1(4) |
| F(1)-C(18)-C(14) | 113.2(3) |

Symmetry transformations used to generate equivalent atoms:

**Supplementary Table 9. Anisotropic displacement parameters ($Å^2 x 10^3$) for t_a. The anisotropic displacement factor exponent takes the form: $-2\pi^2[ h^2 a^{*2}U^{11} + ... + 2 h k a^* b^* U^{12} ]$**

| | $U^{11}$ | $U^{22}$ | $U^{33}$ | $U^{23}$ | $U^{13}$ | $U^{12}$ |
|---|---|---|---|---|---|---|
| F(1) | 100(2) | 104(3) | 44(2) | -18(2) | 30(1) | -49(2) |
| F(2) | 71(2) | 121(3) | 32(1) | -20(2) | 0(1) | -15(2) |
| F(3) | 97(2) | 110(3) | 58(2) | -11(2) | 28(2) | 46(2) |
| O(1) | 29(1) | 19(1) | 35(1) | -3(1) | 7(1) | -4(1) |
| O(2) | 24(1) | 33(1) | 44(1) | 2(1) | 4(1) | -6(1) |
| O(3) | 26(1) | 35(1) | 32(1) | -5(1) | -2(1) | 2(1) |
| O(4) | 39(1) | 24(1) | 36(1) | 6(1) | -8(1) | -8(1) |
| O(5) | 23(1) | 30(1) | 31(1) | -4(1) | 3(1) | -4(1) |
| C(1) | 26(2) | 21(2) | 32(2) | 5(1) | 3(1) | 4(1) |
| C(2) | 28(2) | 26(2) | 28(2) | 1(1) | 6(1) | -1(1) |
| C(3) | 24(1) | 23(2) | 29(2) | -1(1) | 3(1) | -2(1) |
| C(4) | 26(2) | 19(2) | 32(2) | -2(1) | 4(1) | 0(1) |
| C(5) | 28(1) | 24(2) | 30(2) | -3(1) | 10(1) | -6(1) |
| C(6) | 29(2) | 23(2) | 28(2) | -1(1) | 3(1) | 0(1) |
| C(7) | 22(1) | 20(2) | 31(2) | -2(1) | 4(1) | -1(1) |
| C(8) | 25(1) | 20(2) | 32(2) | -2(1) | 4(1) | -1(1) |
| C(9) | 24(1) | 25(2) | 28(2) | 0(1) | 4(1) | -1(1) |
| C(10) | 29(2) | 16(2) | 32(2) | -1(1) | 4(1) | -4(1) |
| C(11) | 22(1) | 27(2) | 31(2) | 1(1) | 3(1) | -4(1) |
| C(12) | 23(1) | 19(2) | 28(2) | 0(1) | -1(1) | 2(1) |
| C(13) | 25(1) | 31(2) | 28(2) | -1(1) | 2(1) | 1(1) |
| C(14) | 31(2) | 35(2) | 30(2) | -3(2) | 2(1) | 2(2) |
| C(15) | 38(2) | 39(2) | 35(2) | -8(2) | -4(1) | -3(2) |
| C(16) | 25(1) | 30(2) | 42(2) | -1(2) | 4(1) | -4(1) |
| C(17) | 25(2) | 25(2) | 34(2) | 2(2) | 4(1) | 2(1) |
| C(18) | 43(2) | 59(3) | 32(2) | -7(2) | 5(2) | -6(2) |

**Supplementary Table 10. Hydrogen coordinates ( x $10^4$) and isotropic displacement parameters (Å$^2$x 10 $^3$) for t_a.**

| | x | y | z | U(eq) |
|---|---|---|---|---|
| H(2) | -3300(70) | 2520(50) | 100(30) | 46 |
| H(1) | 5370(80) | 8070(60) | 1420(30) | 50 |
| H(2A) | -1232 | 1381 | 3112 | 33 |
| H(2B) | -2637 | 2403 | 2454 | 33 |
| H(3) | 1864 | 1999 | 2432 | 30 |
| H(4) | 2164 | 361 | 1354 | 31 |
| H(5A) | 2301 | 1807 | 150 | 32 |
| H(5B) | -41 | 1109 | -145 | 32 |
| H(6) | -57 | 3672 | 35 | 32 |
| H(7) | -691 | 3994 | 1748 | 29 |
| H(8) | 3857 | 3625 | 1207 | 31 |
| H(9) | 1756 | 5794 | 2018 | 30 |
| H(10) | 6188 | 5552 | 1354 | 31 |
| H(11A) | 5063 | 6998 | 2976 | 32 |
| H(11B) | 6328 | 5562 | 2988 | 32 |
| H(13) | 6741 | 7573 | 4209 | 34 |
| H(15) | 11846 | 10058 | 5020 | 45 |
| H(16) | 13486 | 9727 | 3606 | 39 |
| H(17) | 11716 | 8367 | 2494 | 33 |

## Supplementary Table 11. Torsion angles [°] for t_a.

| | |
|---|---|
| C(4)-O(1)-C(1)-O(2) | 179.6(3) |
| C(4)-O(1)-C(1)-C(2) | -2.4(4) |
| O(2)-C(1)-C(2)-C(3) | -177.6(3) |
| O(1)-C(1)-C(2)-C(3) | 4.6(4) |
| C(1)-C(2)-C(3)-C(4) | -4.8(3) |
| C(1)-C(2)-C(3)-C(7) | 110.2(3) |
| C(1)-O(1)-C(4)-C(5) | -115.6(3) |
| C(1)-O(1)-C(4)-C(3) | -0.8(3) |
| C(2)-C(3)-C(4)-O(1) | 3.5(3) |
| C(7)-C(3)-C(4)-O(1) | -117.5(3) |
| C(2)-C(3)-C(4)-C(5) | 121.0(3) |
| C(7)-C(3)-C(4)-C(5) | 0.0(3) |
| O(1)-C(4)-C(5)-C(6) | 90.9(3) |
| C(3)-C(4)-C(5)-C(6) | -23.9(3) |
| C(4)-C(5)-C(6)-O(3) | -74.8(3) |
| C(4)-C(5)-C(6)-C(7) | 38.9(3) |
| O(3)-C(6)-C(7)-C(8) | -159.3(3) |
| C(5)-C(6)-C(7)-C(8) | 83.2(3) |
| O(3)-C(6)-C(7)-C(3) | 79.2(3) |
| C(5)-C(6)-C(7)-C(3) | -38.3(3) |
| C(2)-C(3)-C(7)-C(8) | 148.9(3) |
| C(4)-C(3)-C(7)-C(8) | -97.0(3) |
| C(2)-C(3)-C(7)-C(6) | -90.7(3) |
| C(4)-C(3)-C(7)-C(6) | 23.4(3) |
| C(6)-C(7)-C(8)-C(9) | 120.4(4) |
| C(3)-C(7)-C(8)-C(9) | -123.8(3) |
| C(7)-C(8)-C(9)-C(10) | -176.7(3) |
| C(8)-C(9)-C(10)-O(4) | 123.2(3) |
| C(8)-C(9)-C(10)-C(11) | -116.1(4) |
| C(12)-O(5)-C(11)-C(10) | 159.7(3) |
| O(4)-C(10)-C(11)-O(5) | -63.0(3) |
| C(9)-C(10)-C(11)-O(5) | 178.0(3) |
| C(11)-O(5)-C(12)-C(17) | -179.3(3) |
| C(11)-O(5)-C(12)-C(13) | 0.3(5) |
| O(5)-C(12)-C(13)-C(14) | -178.3(3) |
| C(17)-C(12)-C(13)-C(14) | 1.3(5) |
| C(12)-C(13)-C(14)-C(15) | -0.9(5) |

| | |
|---|---|
| C(12)-C(13)-C(14)-C(18) | 175.9(3) |
| C(13)-C(14)-C(15)-C(16) | -0.1(6) |
| C(18)-C(14)-C(15)-C(16) | -177.0(4) |
| C(14)-C(15)-C(16)-C(17) | 0.8(6) |
| C(15)-C(16)-C(17)-C(12) | -0.5(5) |
| O(5)-C(12)-C(17)-C(16) | 179.1(3) |
| C(13)-C(12)-C(17)-C(16) | -0.6(5) |
| C(15)-C(14)-C(18)-F(2) | -32.6(6) |
| C(13)-C(14)-C(18)-F(2) | 150.4(4) |
| C(15)-C(14)-C(18)-F(3) | 88.4(5) |
| C(13)-C(14)-C(18)-F(3) | -88.6(5) |
| C(15)-C(14)-C(18)-F(1) | -153.8(4) |
| C(13)-C(14)-C(18)-F(1) | 29.2(5) |

Symmetry transformations used to generate equivalent atoms:

## Supplementary Table 12. Hydrogen bonds for t_a [Å and °].

| D-H...A | d(D-H) | d(H...A) | d(D...A) | <(DHA) |
|---|---|---|---|---|

## III Supplementary References

1. Jing, C. & Aggarwal, V. K. Total synthesis of thromboxane $B_2$ via a key bicyclic enal intermediate. *Org. Lett.* **22**, 6505–6509 (2020).
2. Prévost, S. et al. Synthesis of prostaglandin analogues, latanoprost and bimatoprost, using organocatalysis via a key bicyclic enal intermediate. *Org. Lett.* **17**, 504–507 (2015).
3. Wang, S., Qian, Q. & Gong, H. Nickel-Catalyzed Reductive Coupling of Aryl Halides with Secondary Alkyl Bromides and Allylic Acetate. *Org. Lett.* **14**, 3352–3355 (2012).
